# Supplementary material for: First Trifluoromethylated Phenanthrolinediamides: Synthesis, Structure, Stereodynamics and Complexation with Ln(III)
Source: Molecules. 2022 May 12;27(10):3114. doi: 10.3390/molecules27103114 (PMC9143926; doi:10.3390/molecules27103114)
Supplement: Supplementary file 1 [file molecules-27-03114-s001.zip › molecules-1693201-supplementary.pdf]

Article

First Trifluoromethylated Phenanthrolinediamides: Synthesis, Structure, Stereodynamics and Complexation with Ln(III)

Yuri A. Ustynyuk [1], Pavel S. Lemport [1], Vitaly A. Roznyatovsky [1], Konstantin A. Lyssenko [1], Alexey O. Gudovannyy [1], Petr I. Matveev [1], Ennie K. Khult [2], Mariia V. Evsiunina [1], Vladimir G. Petrov [1], Igor P. Gloriozov [1], Anton S. Pozdeev [1], Valentine S. Petrov [1], Nane A. Avagyan [1], Alexander S. Aldoshin [1], Stepan N. Kalmykov [1] and Valentine G. Nenajdenko [1,*]

[1] Department of Chemistry, Lomonosov Moscow State University, Leninskie gory 1 bld. 3, Moscow, Russia; nenajdenko@gmail.com (V.N.)
[2] Department of Materials Science, Lomonosov Moscow State University, Leninskie gory 1 bld. 73, Moscow, Russia; jennie.hult@gmail.com (E.K.)
***** Correspondence: nenajdenko@gmail.com (V.N.)

Supplementary Materials

Table of contents

1. NMR, IR and HRMS spectra of synthesized compounds……………………………………S3-S50
2. HPLC chromatogram of ligand **1** isomers………………………………..………………...S51
3. Spectrophotometric titration data…………………………………………..………….S52-S54
4. X-ray analysis data……………………………………………………………………….S55
5. Theoretical computations………………………………………………………………S56-S71

1. NMR, IR and HRMS spectra of synthesized compounds

(2-(trifluoromethyl)pyrrolidin-1-yl)(9-((2-(trifluoromethyl)pyrrolidin-1-yl)carbonyl)-1,10-phenanthrolin-2-yl)methanone (1)

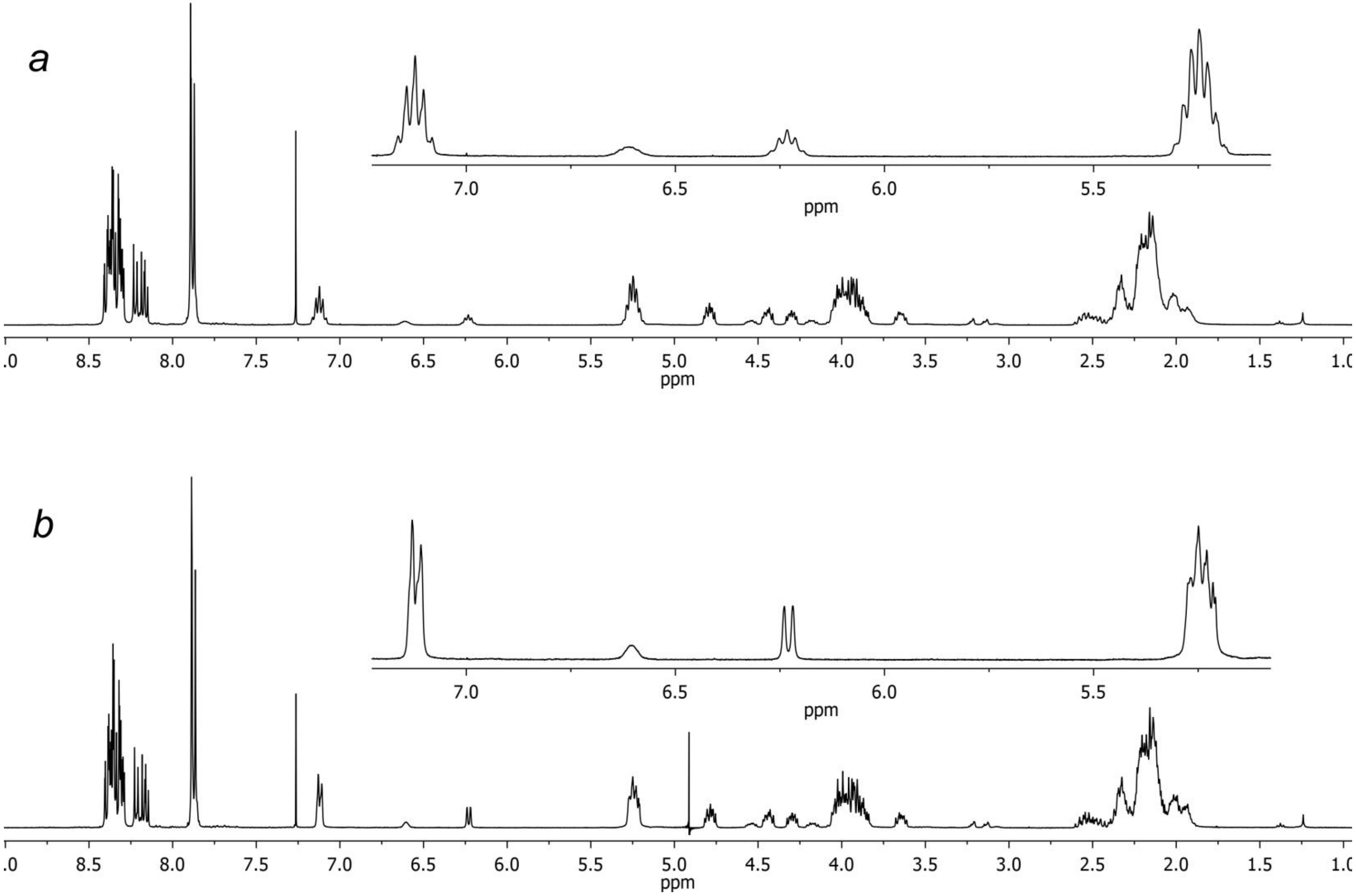

Figure S1. (a) ^{1}H and (b) ^{1}H-{^{19}F} NMR spectra in $CDCl_3$ at 25ºC

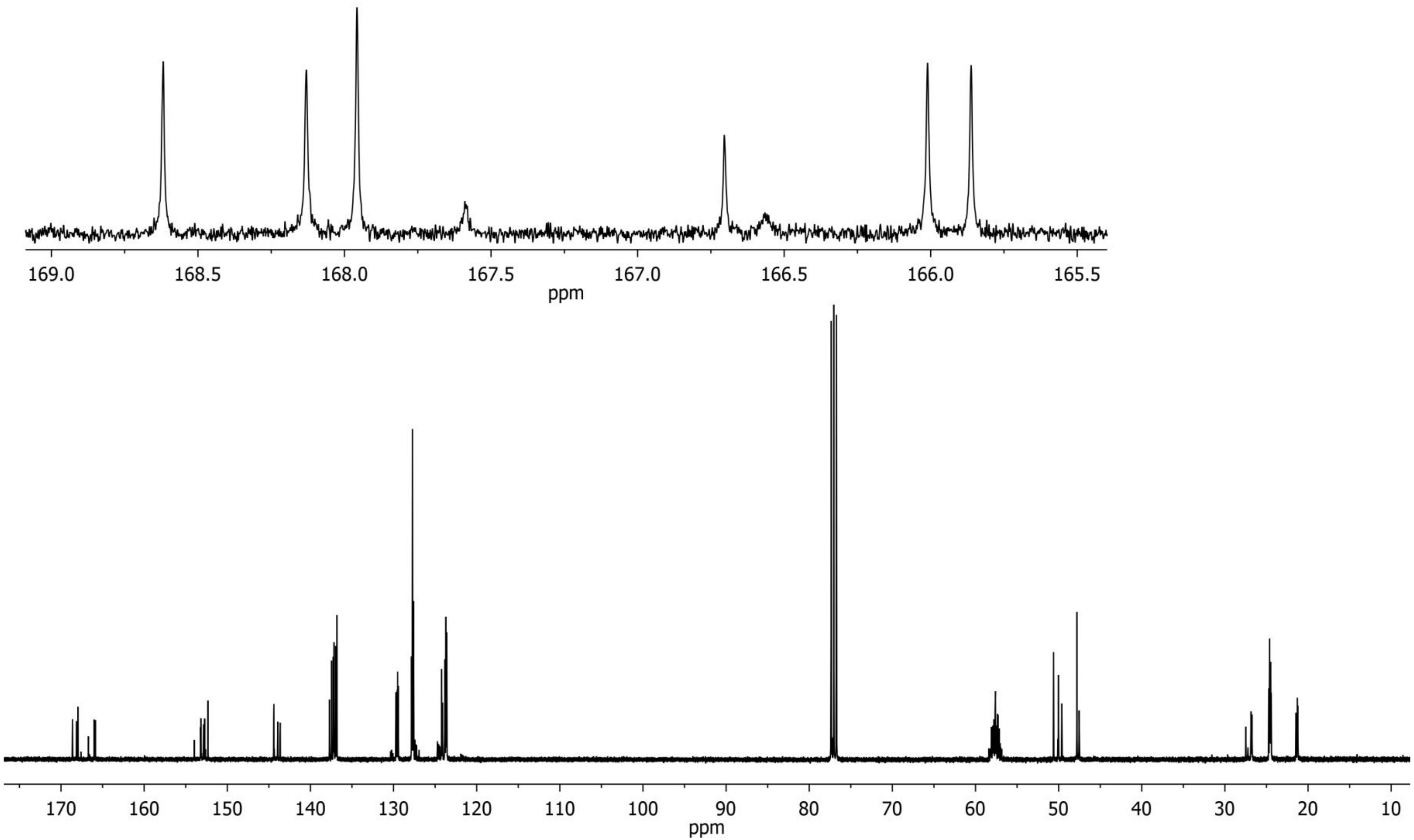

Figure S2. ^{13}C NMR spectra in $CDCl_3$ at 25ºC

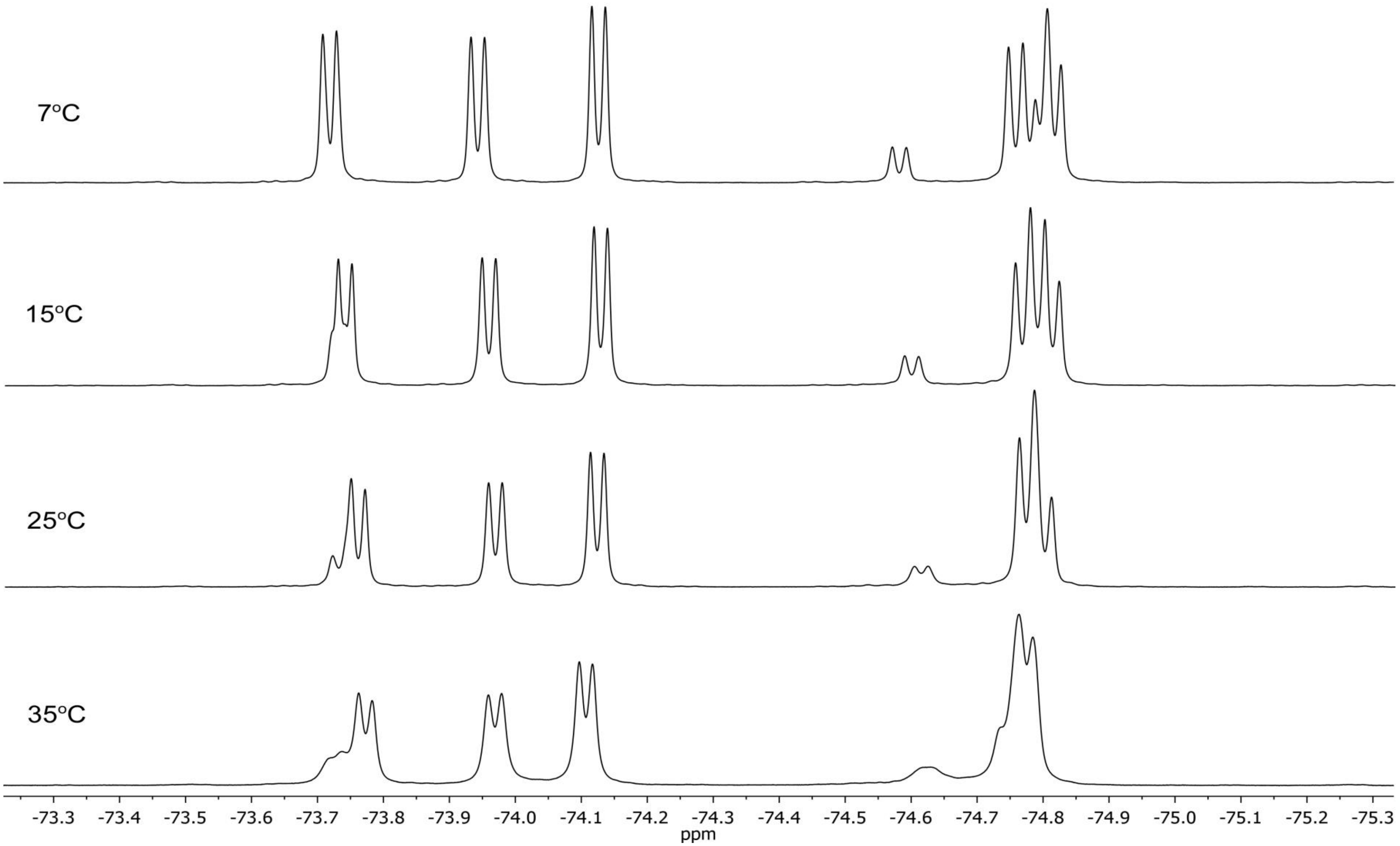

Figure S3. ^{19}F NMR spectra in $CDCl_3$ at 7, 15, 25 and 35ºC (from top to bottom)

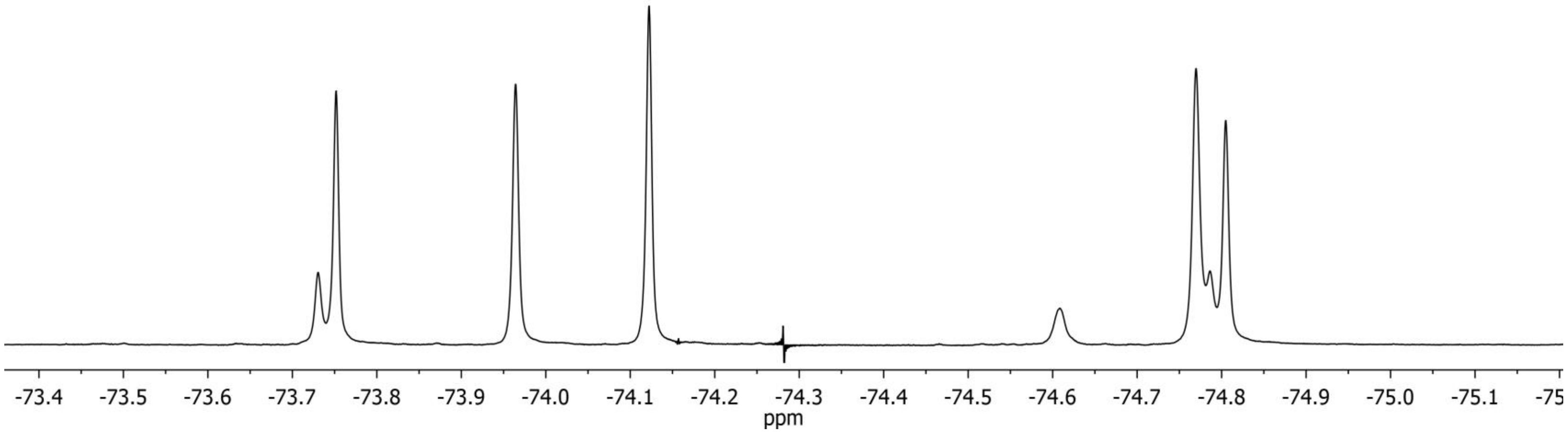

Figure S4. $^{19}F-\{^{1}H\}$ NMR spectra in $CDCl_3$ at 25ºC

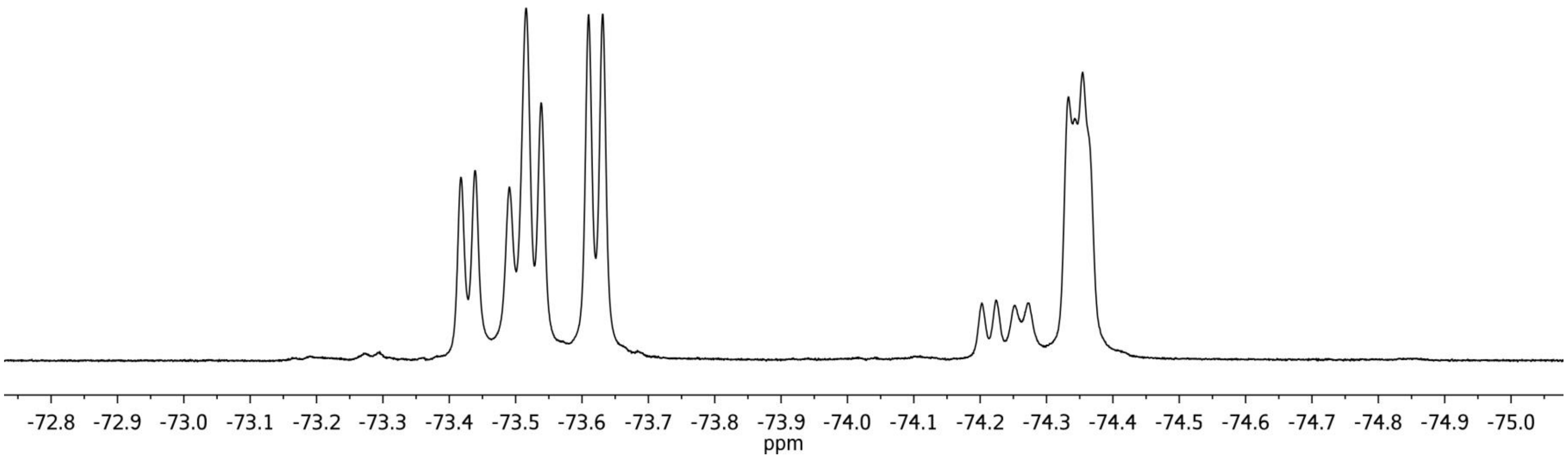

Figure S5. ^{19}F NMR spectra in acetone-d_6 at 25ºC

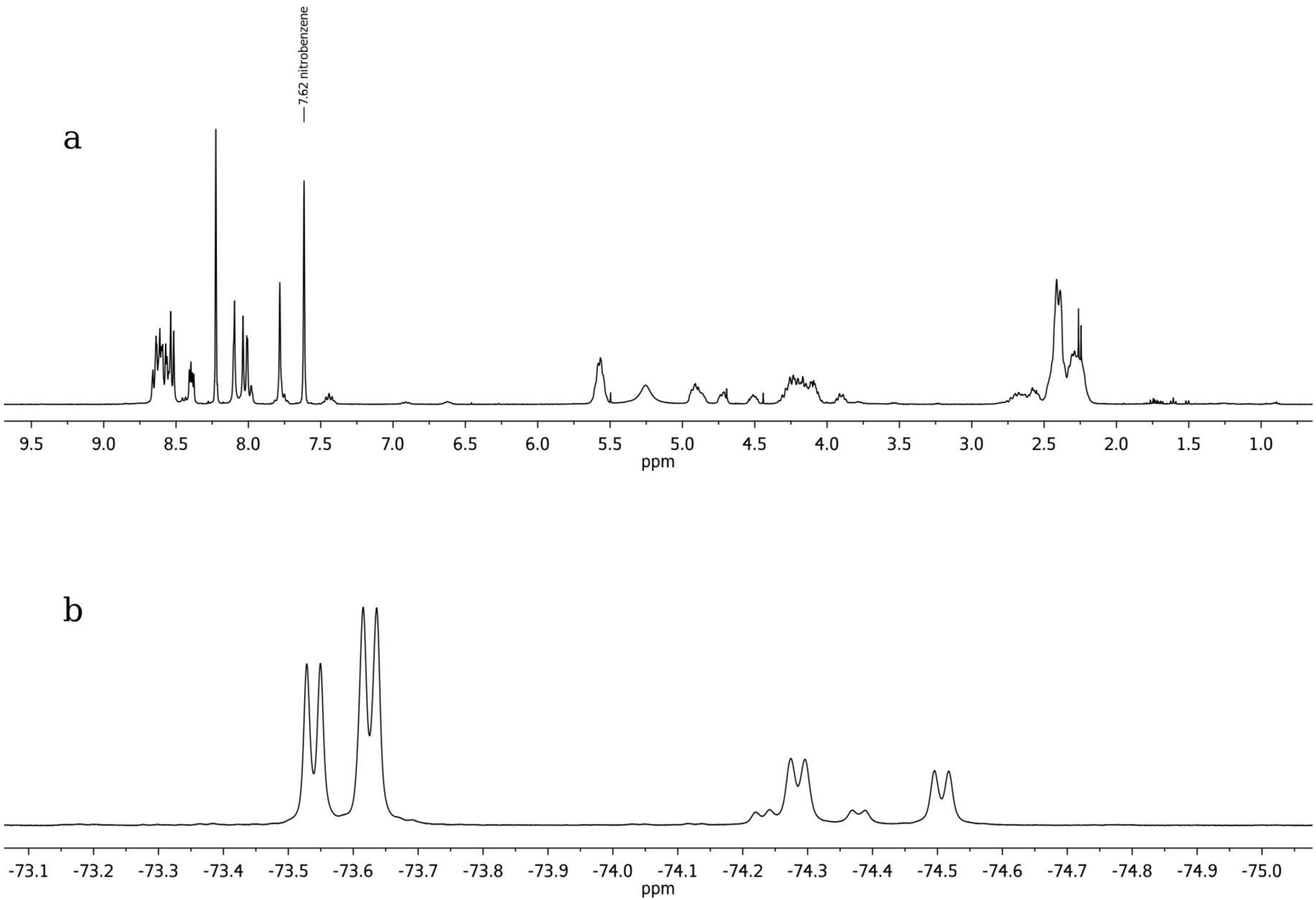

Figure S6. (a) ^{1}H NMR and (b) ^{19}F NMR spectra in $C_6D_5NO_2$ at 25ºC

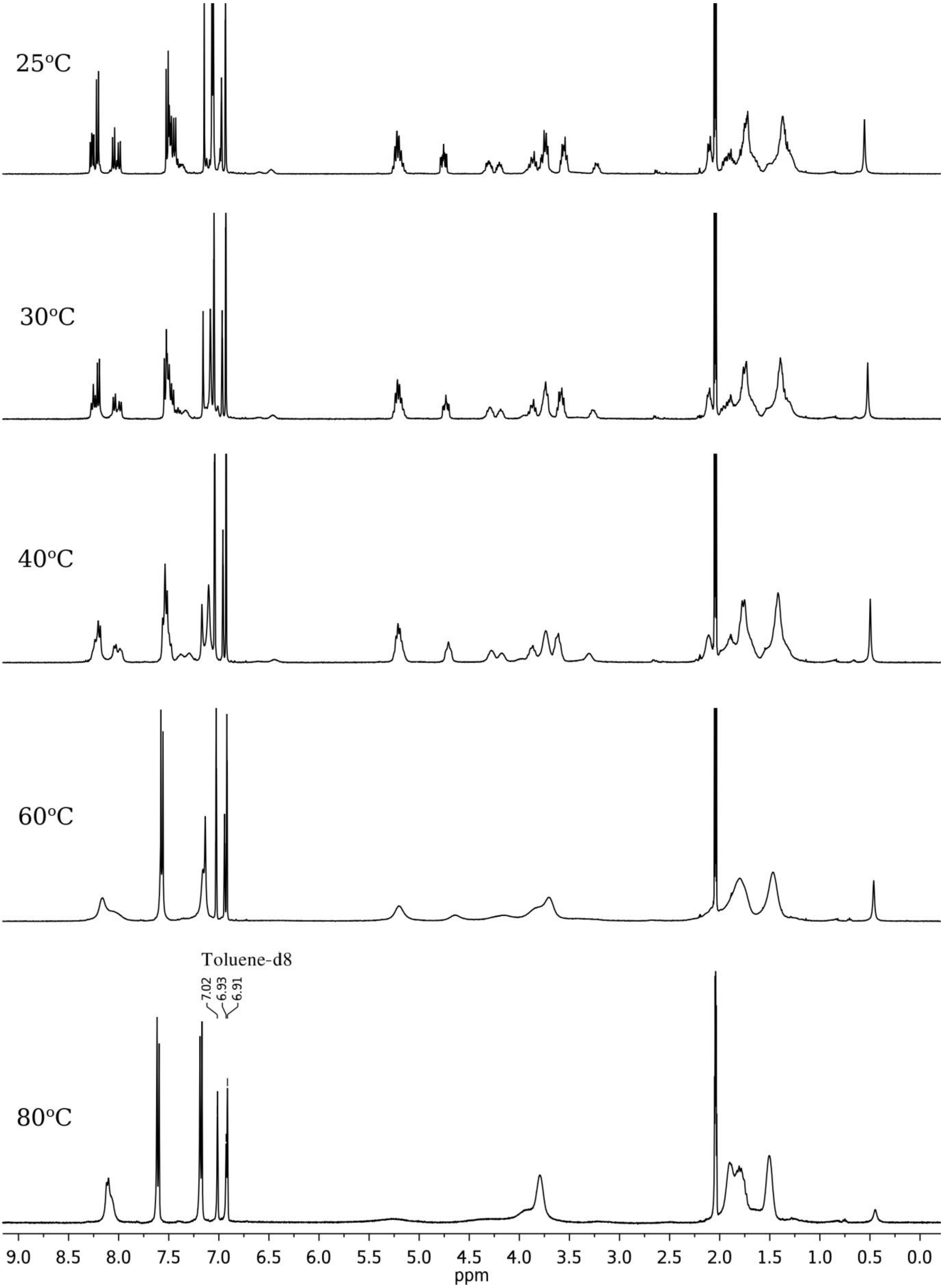

Figure S7. ^{1}H NMR spectra in toluene-d_8 at 25, 30, 40, 60 and 80ºC (from top to bottom)

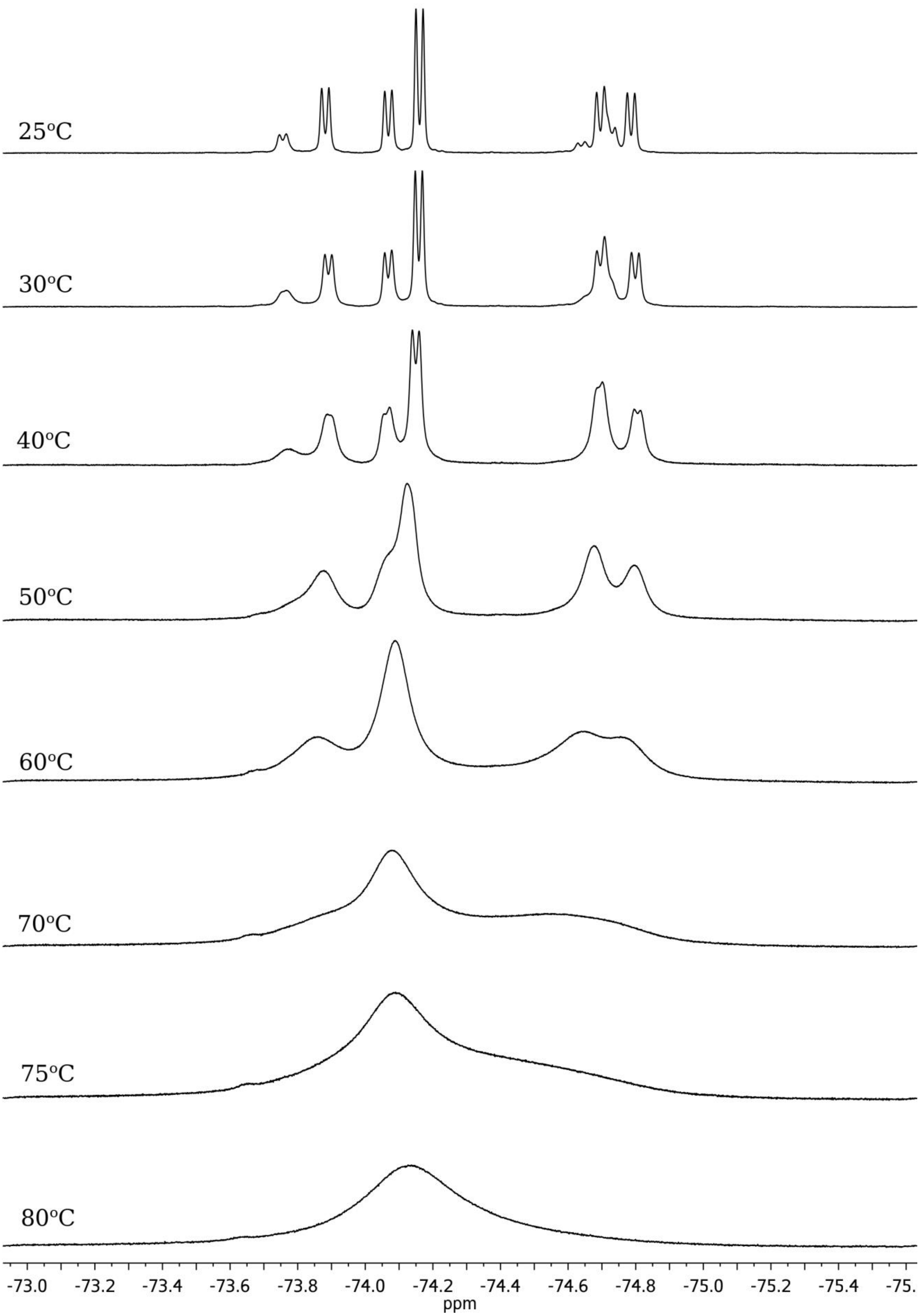

Figure S8. ^{19}F NMR spectra in toluene-d_8 at 25, 30, 40, 50, 60, 70, 75 and 80ºC (from top to bottom)

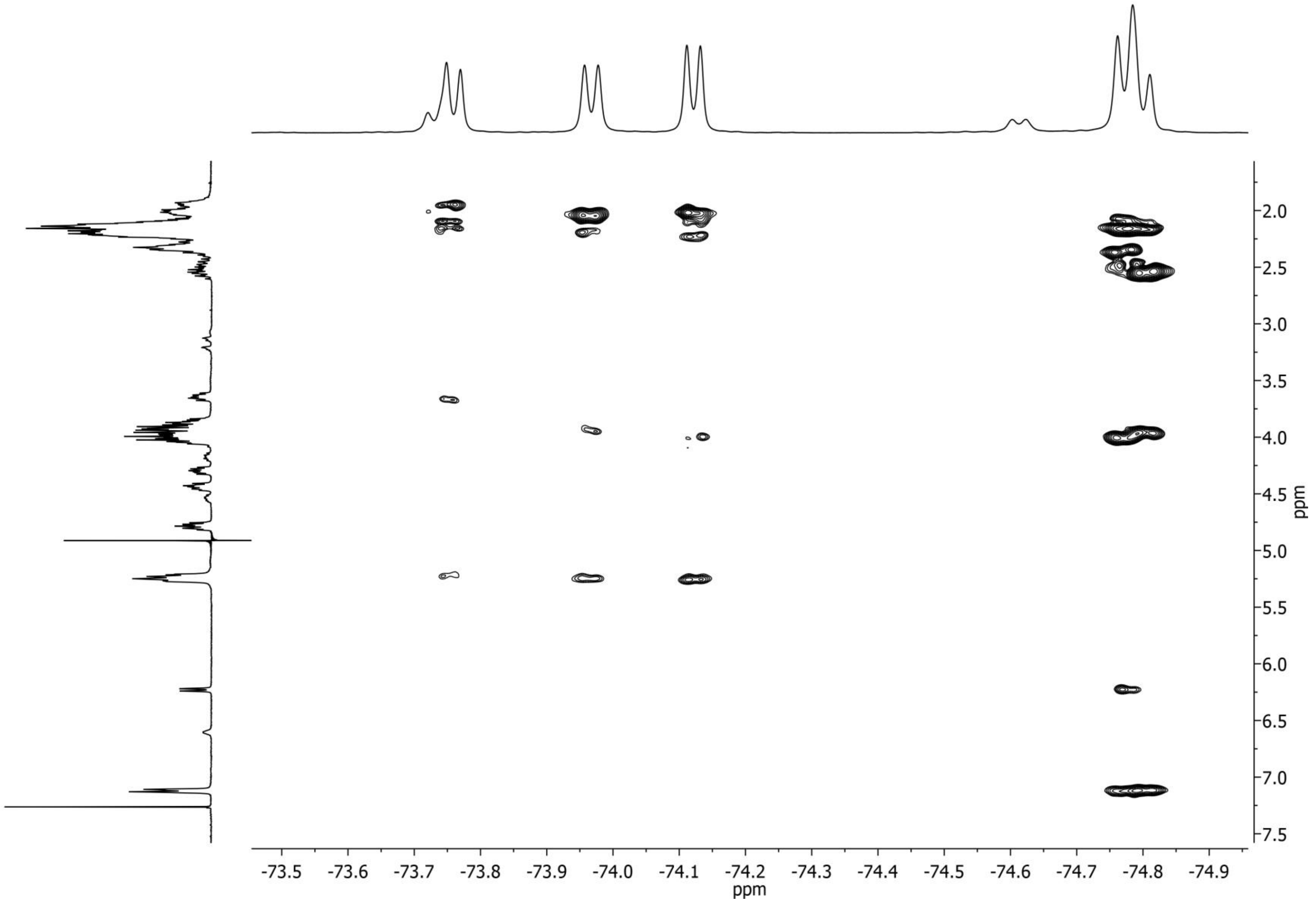

Figure S9. HF_getCOR NMR spectra in $CDCl_3$ at 25ºC

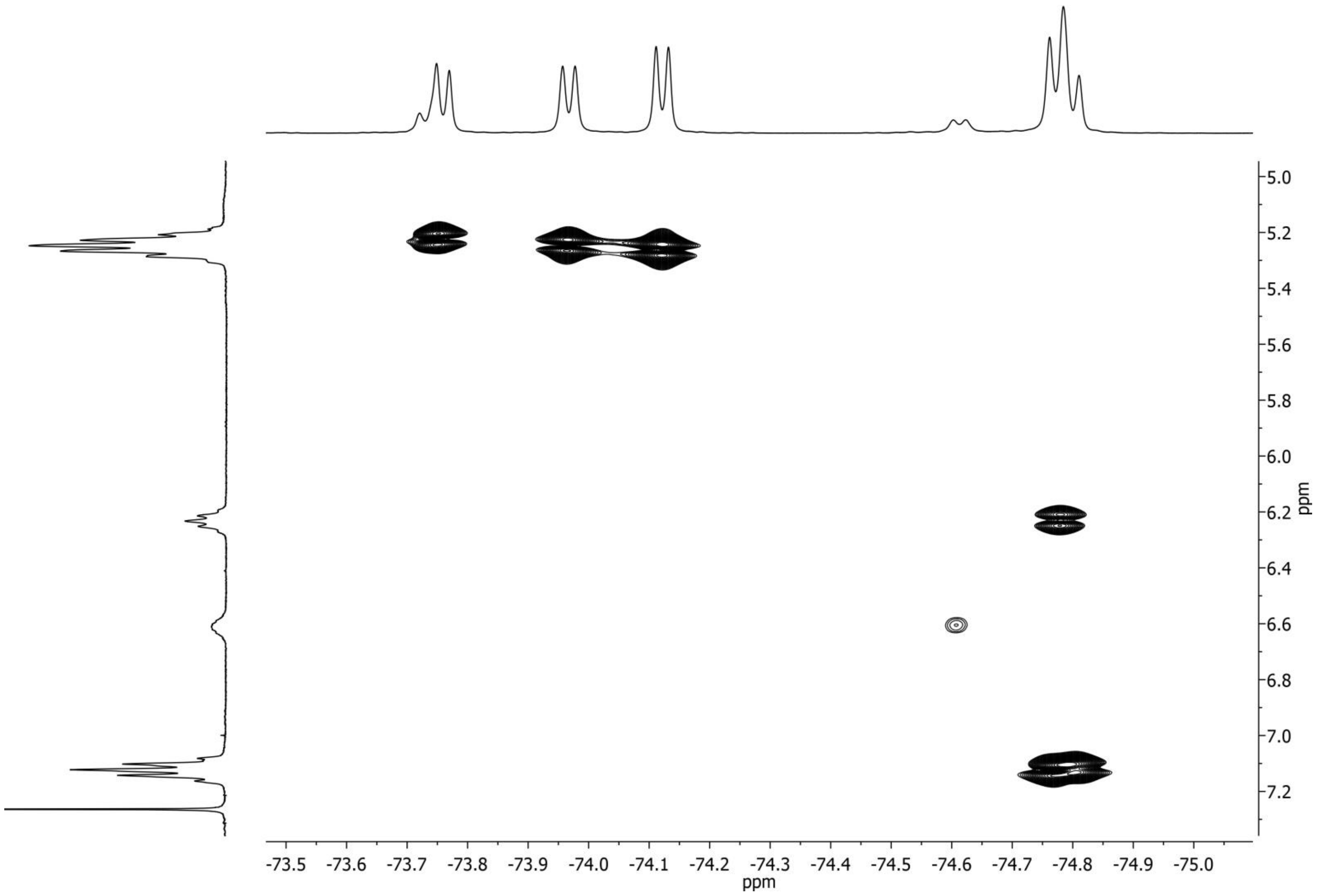

Figure S10. HF_getCOR NMR spectra in $CDCl_3$ at 25ºC, fragmental view

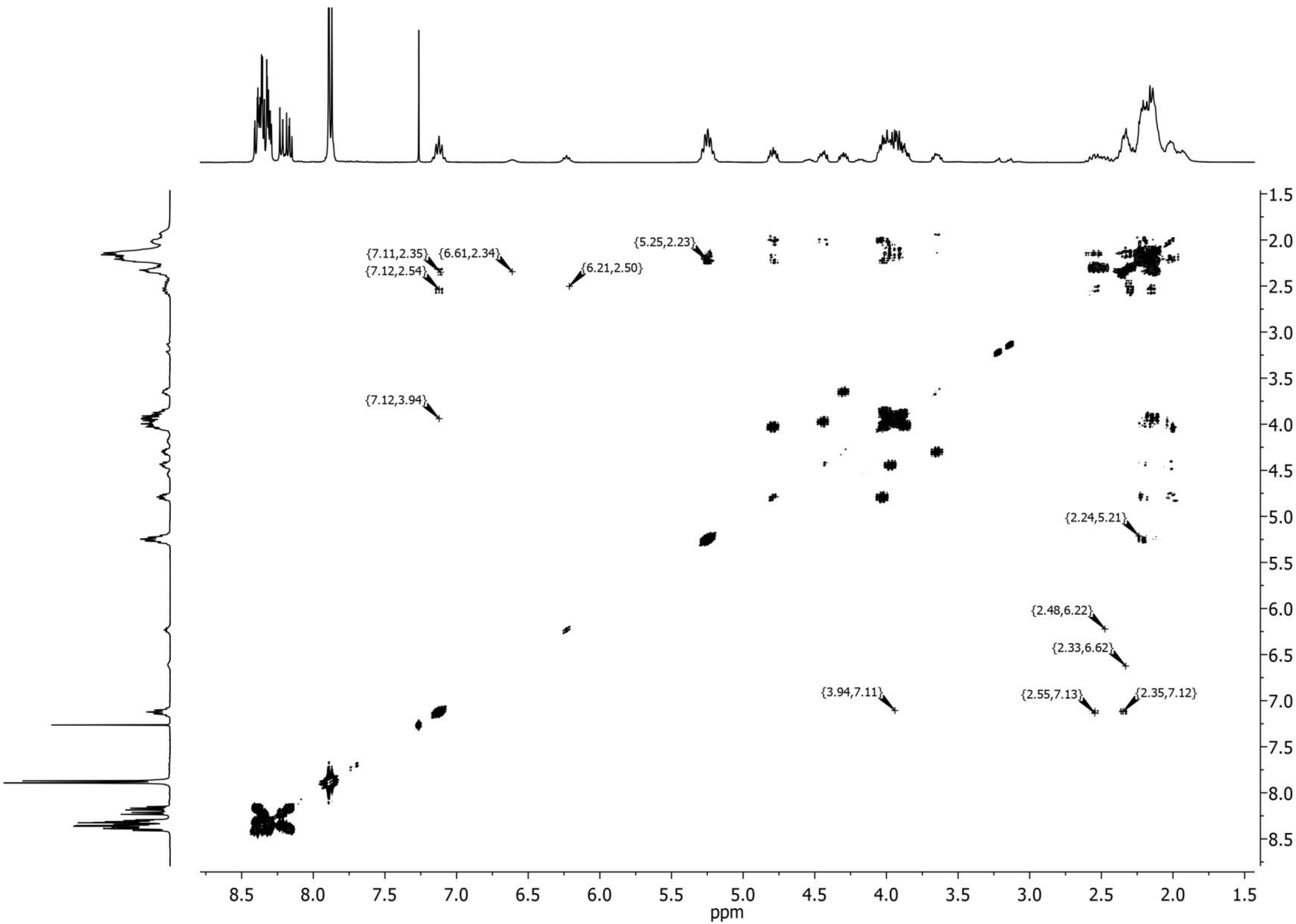

Figure S11. HH gCOSY NMR spectra in $CDCl_3$ at 25ºC

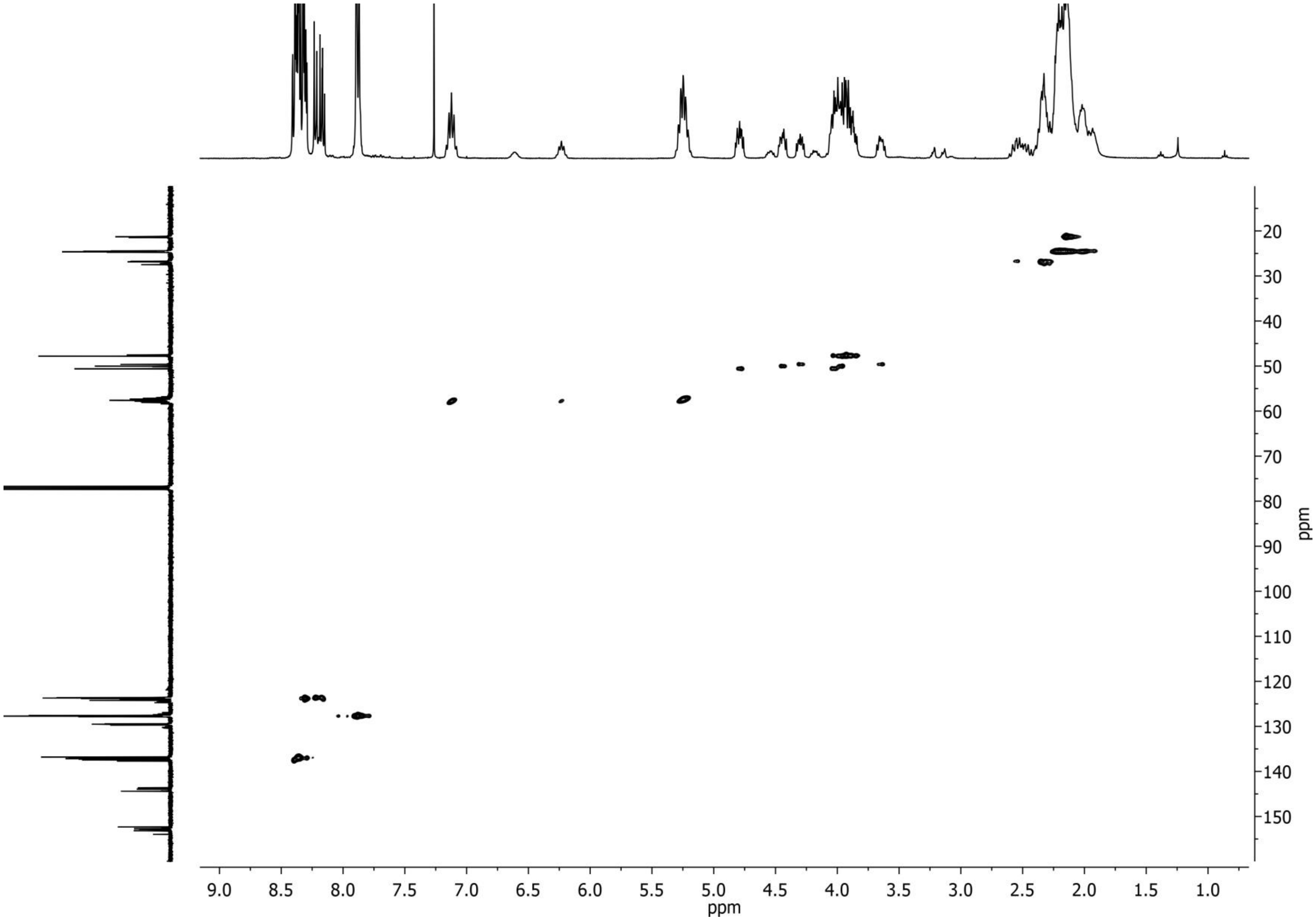

Figure S12. HC gHSQCAD NMR spectra in $CDCl_3$ at 25ºC

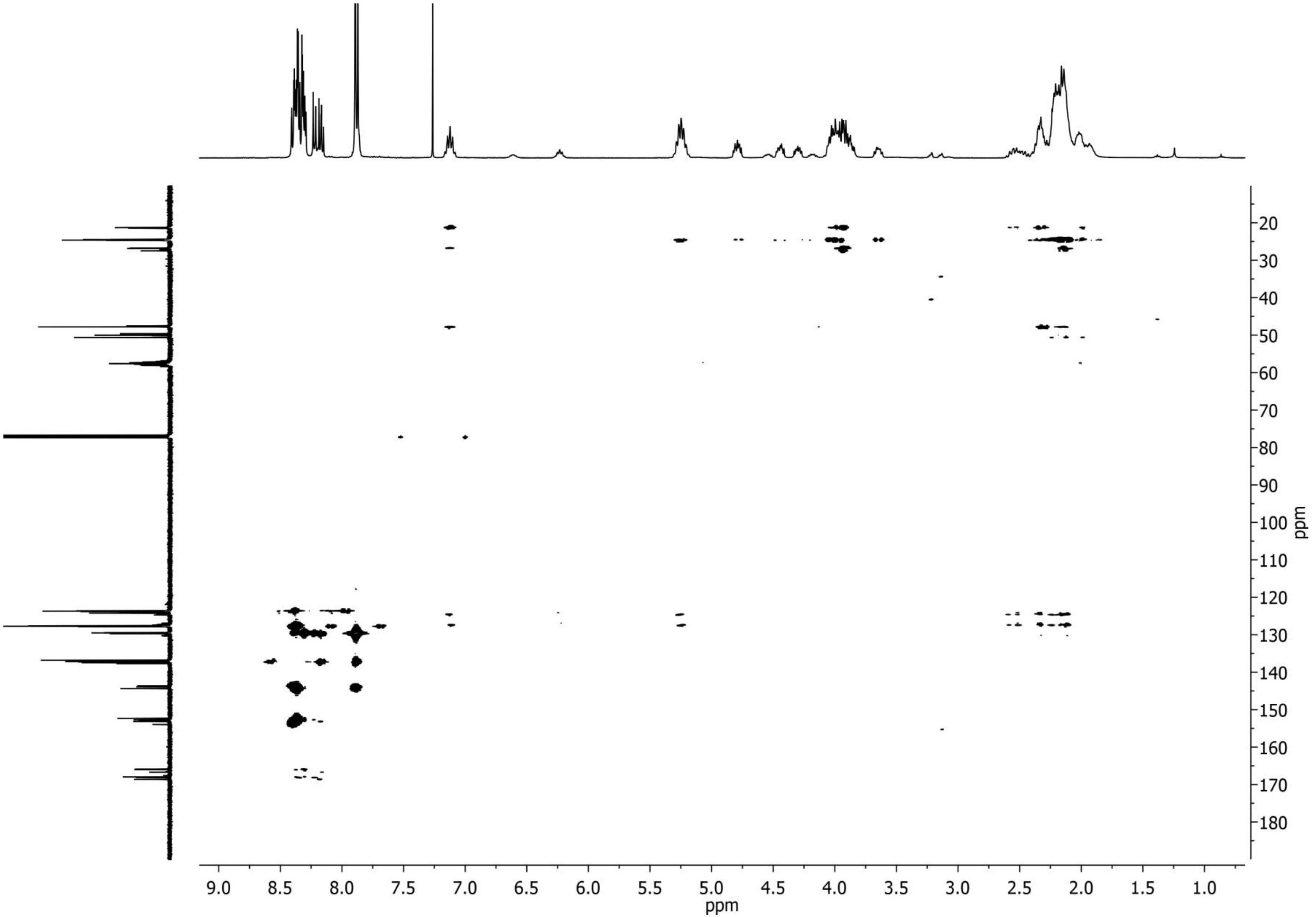

Figure S13. HC gHMBCAD NMR spectra in $CDCl_3$ at 25ºC

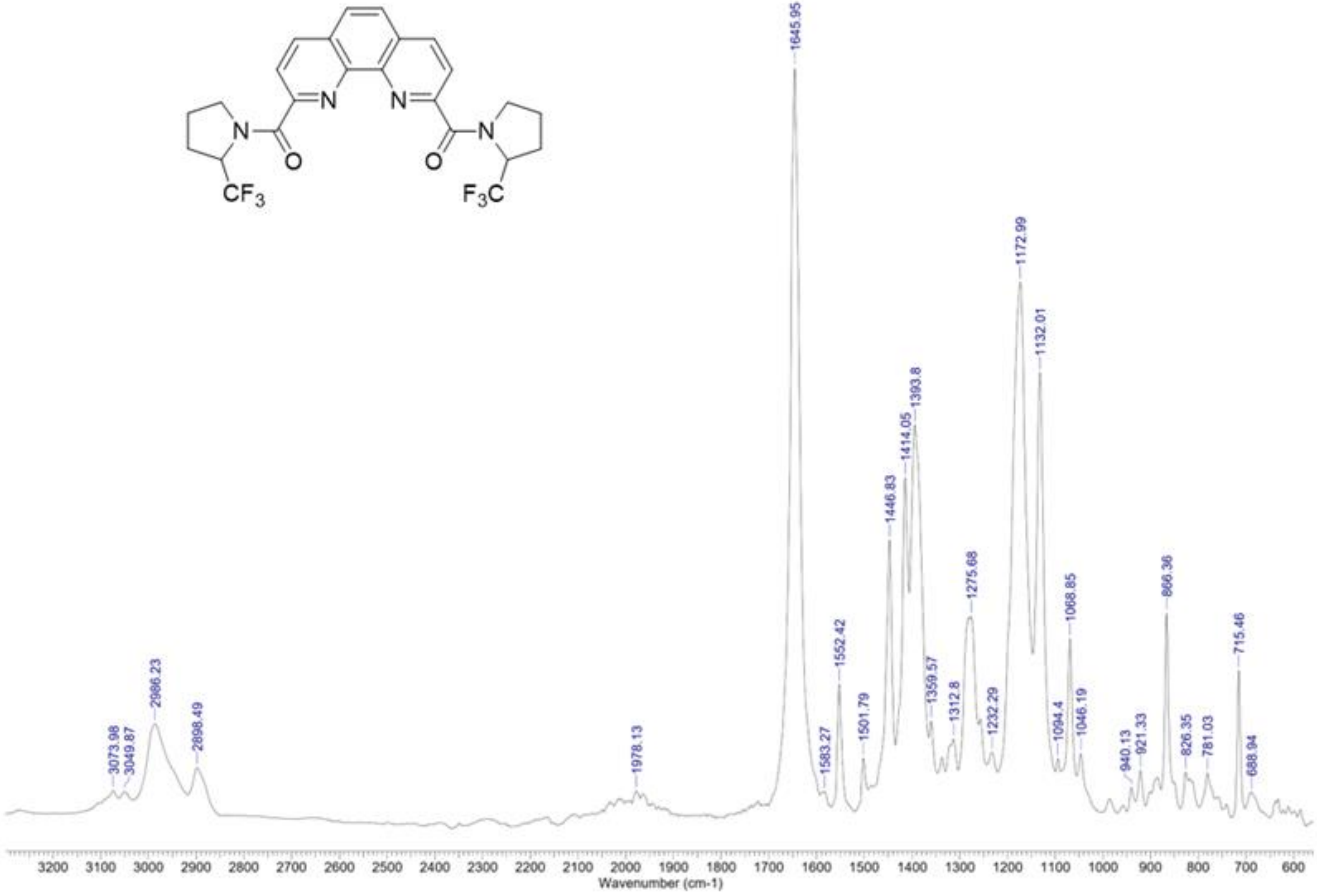

Figure S14. Solid-state IR spectra at 25ºC

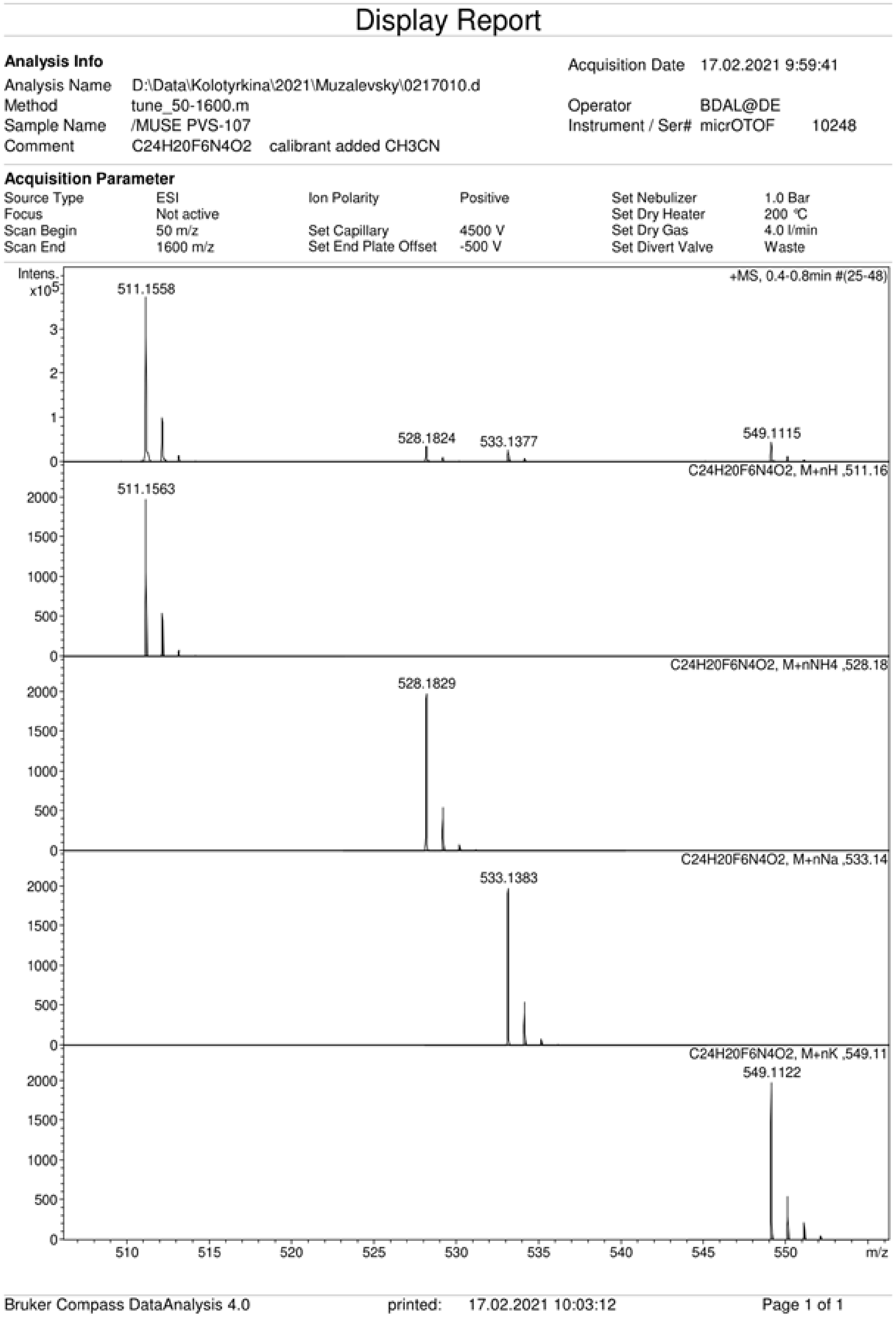

Figure S15. HRMS spectra of (**1**) at 25ºC

(4,7-dichloro-9-((2-(trifluoromethyl)pyrrolidin-1-yl)carbonyl)-1,10-phenanthrolin-2-yl)(2-(trifluoromethyl)pyrrolidin-1-yl)methanone (2)

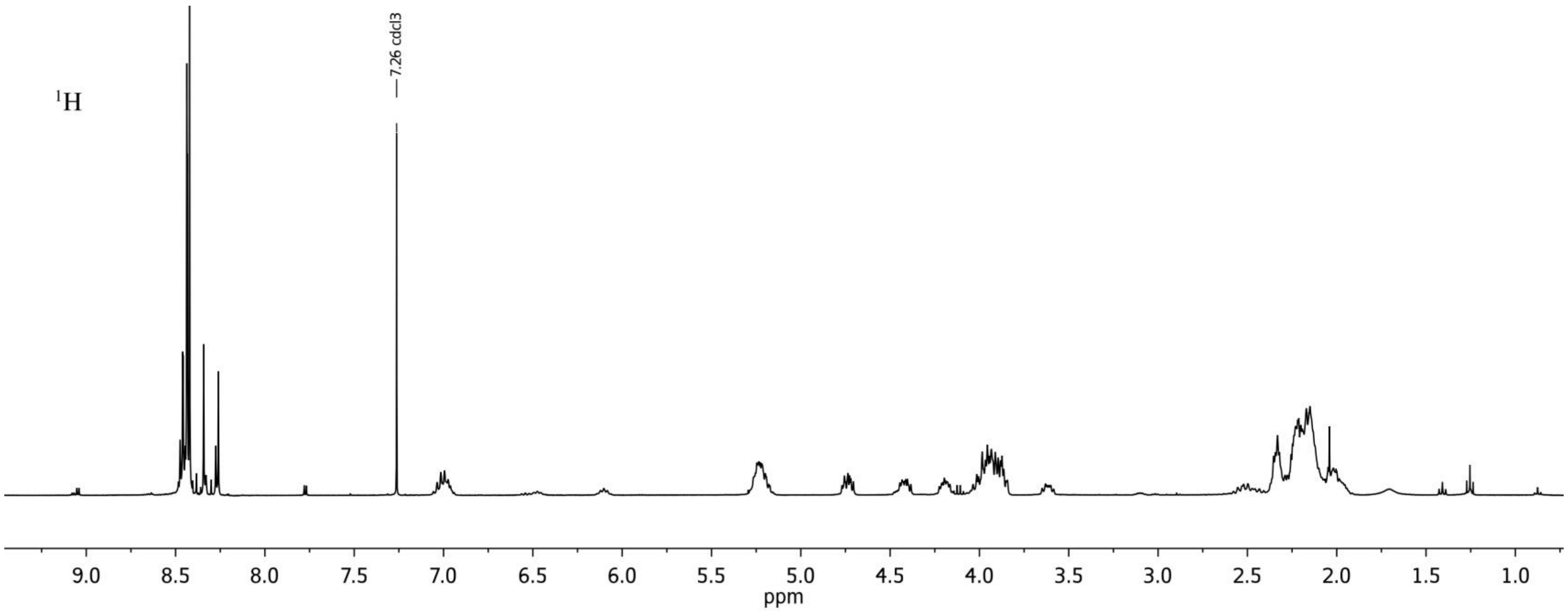

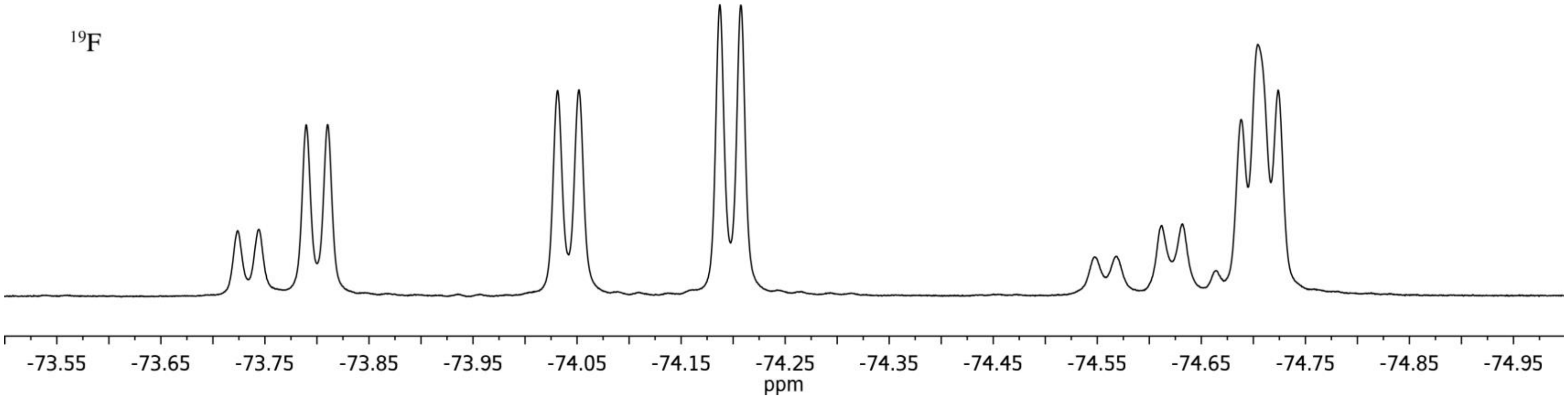

Figure S16. ^{1}H and ^{19}F NMR spectra in $CDCl_3$ at 25ºC

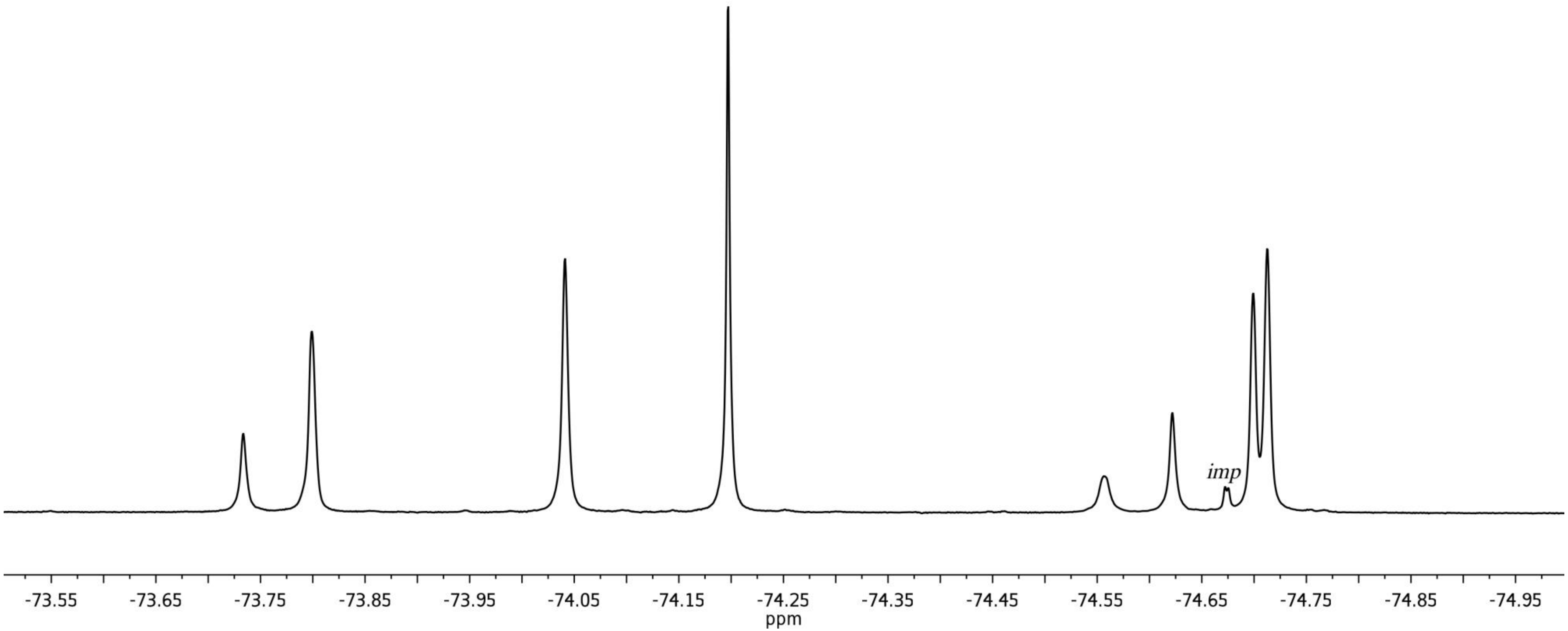

Figure S17. ^{19}F-{^{1}H} NMR spectra in $CDCl_3$ at 25ºC

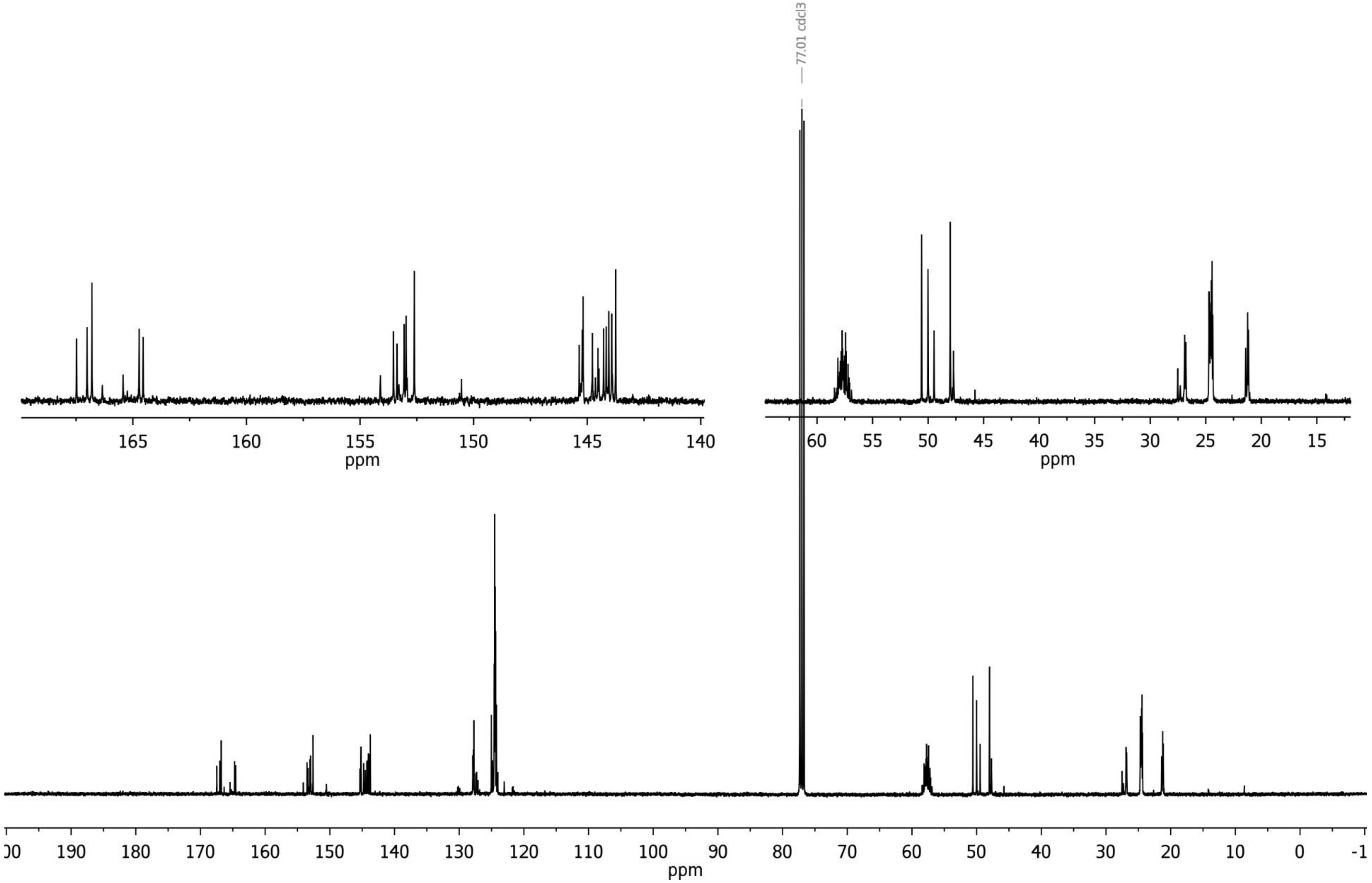

Figure S18. ^{13}C NMR spectra in $CDCl_3$ at 25ºC

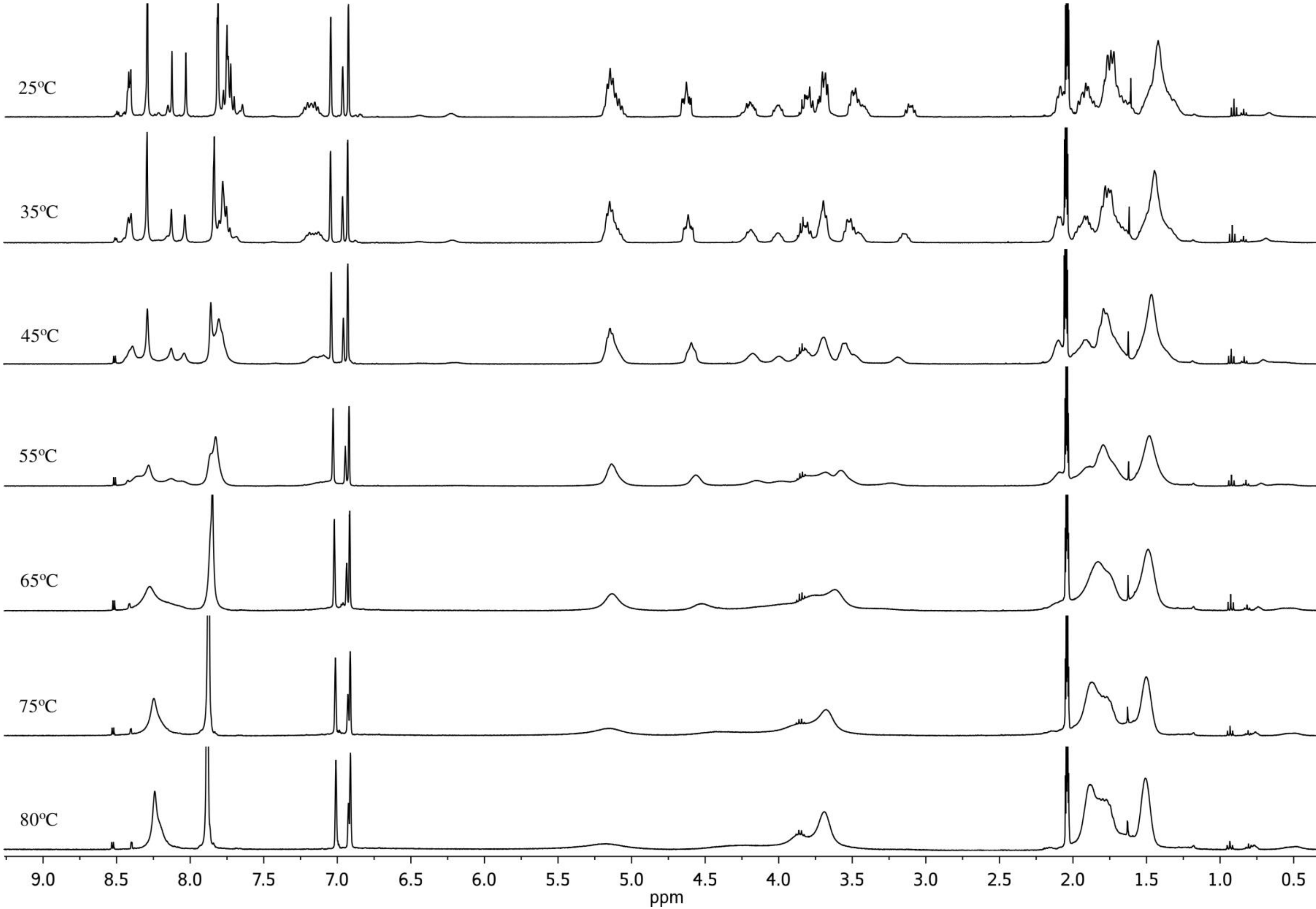

Figure S19. ^{1}H NMR spectra in toluene-d_8 at 25, 35, 45, 55, 65, 75 and 80ºC (from top to bottom)

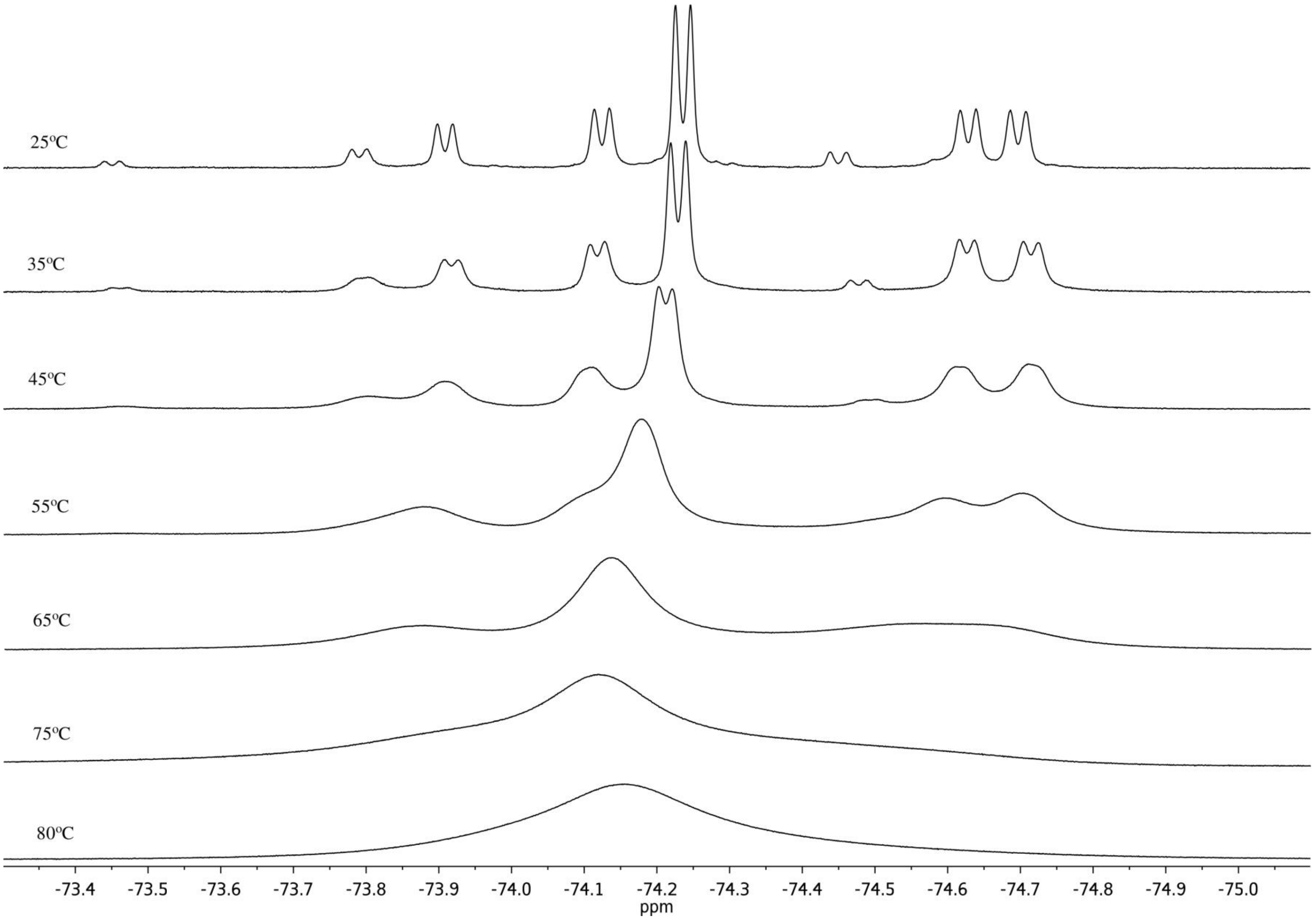

Figure S20. ^{19}F NMR spectra in toluene-d_8 at 25, 35, 45, 55, 65, 75 and 80ºC (from top to bottom)

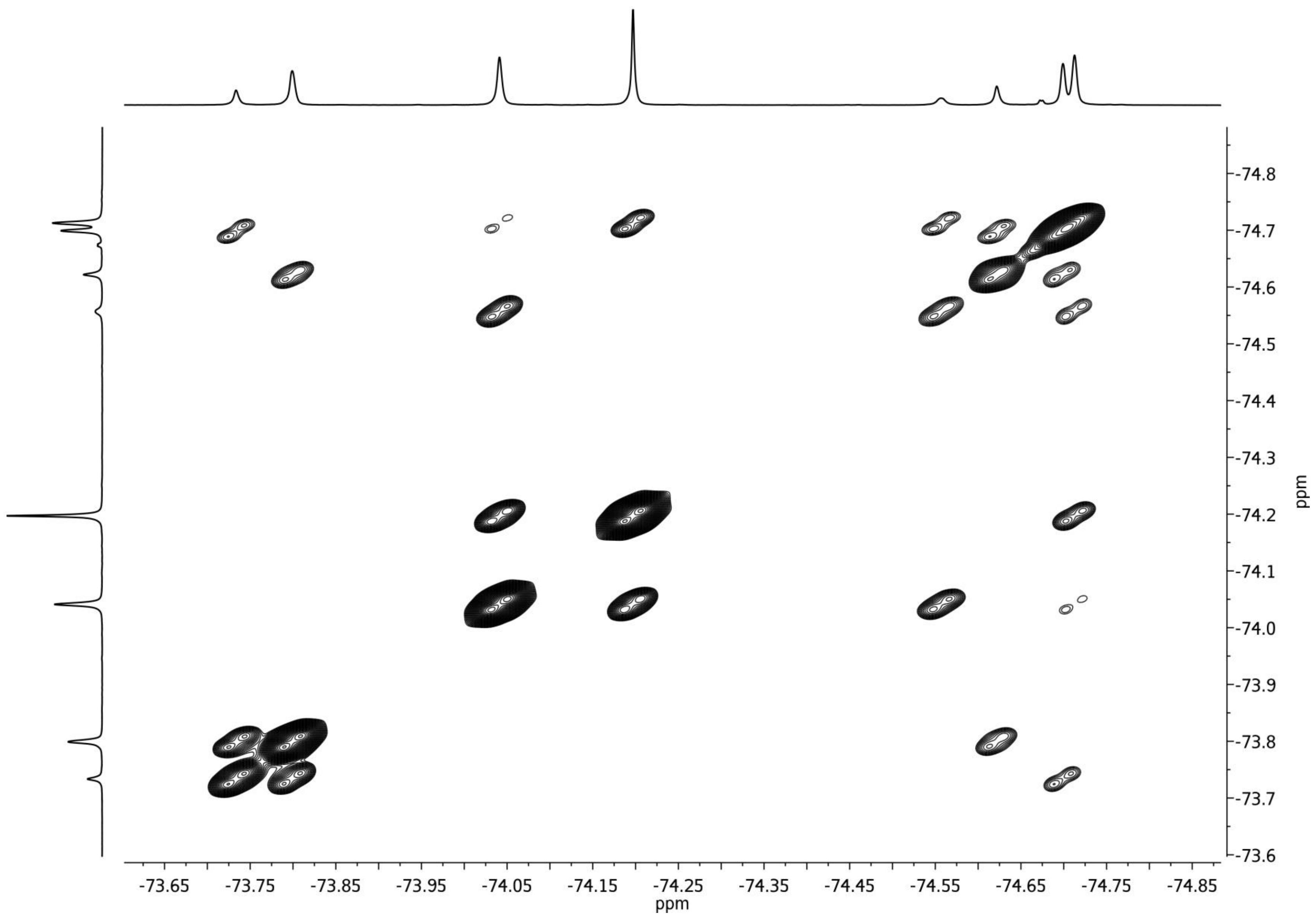

Figure S21. ^{19}F-{^{1}H}/^{19}F-{^{1}H} ROESY NMR spectra in $CDCl_3$ at 25ºC

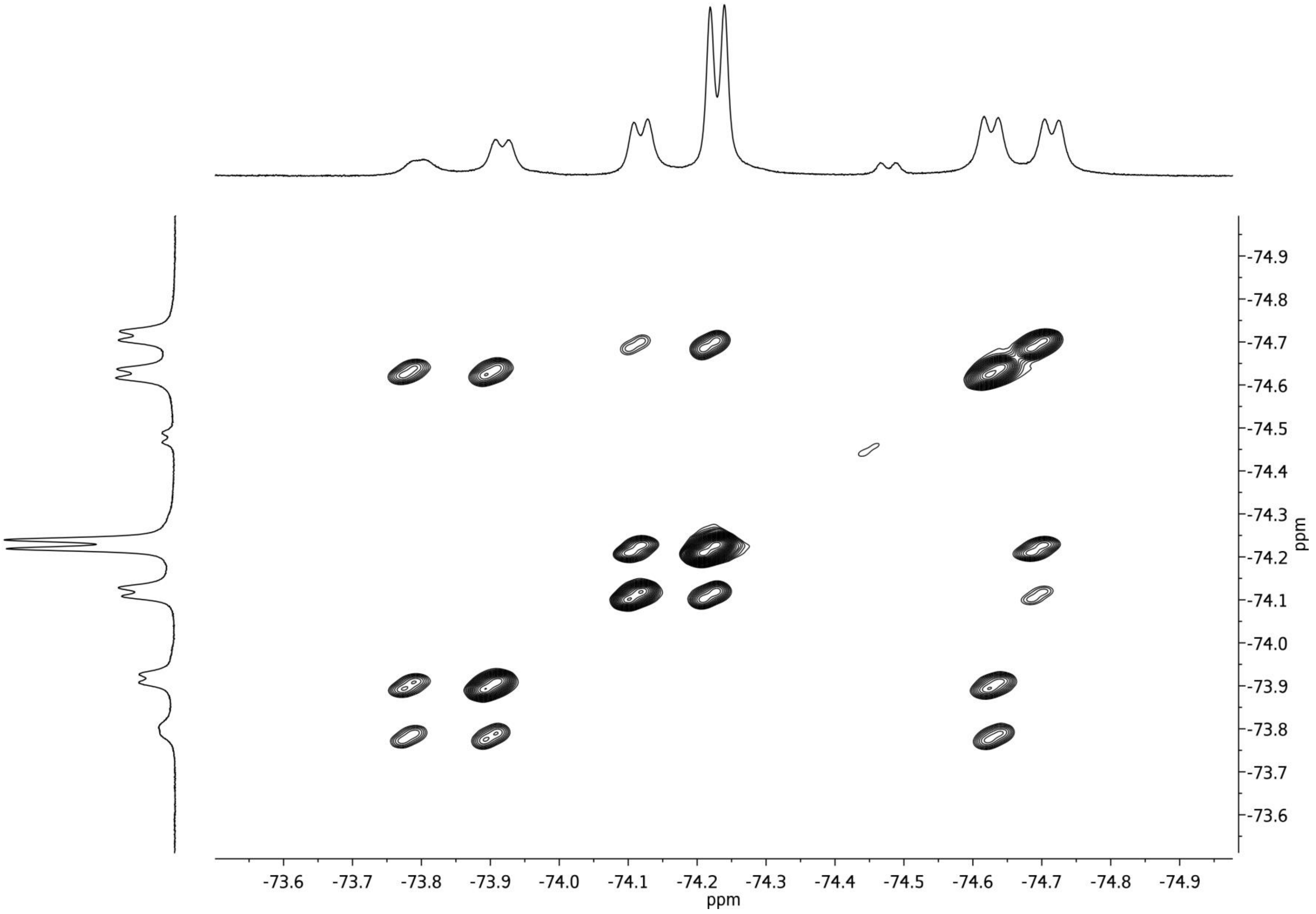

Figure S22. $^{19}F/^{19}F$ ROESY NMR spectra in toluene-d_8 at 25ºC

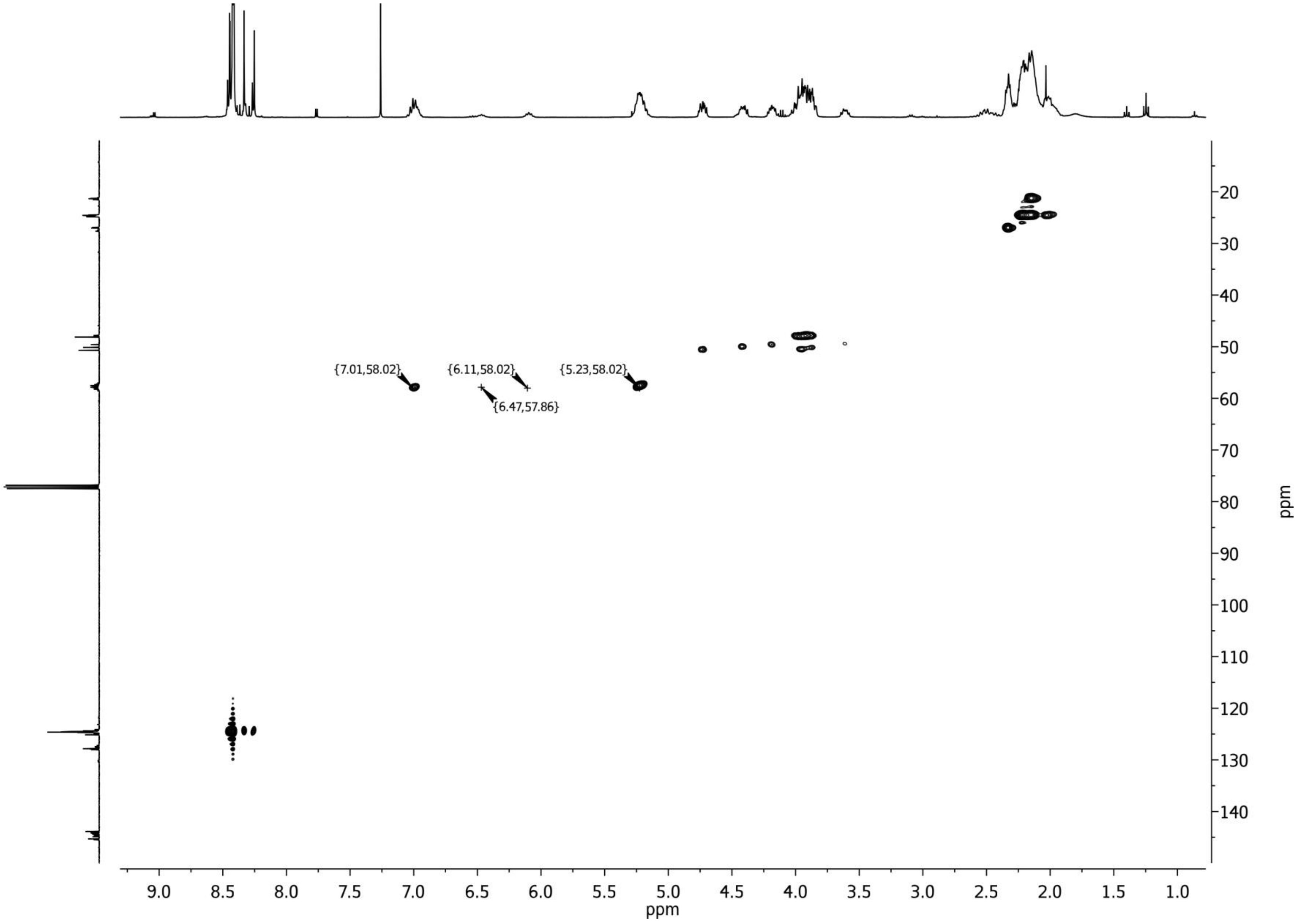

Figure S23. $^{1}H/^{13}C$ HSQC NMR spectra in $CDCl_3$ at 25ºC

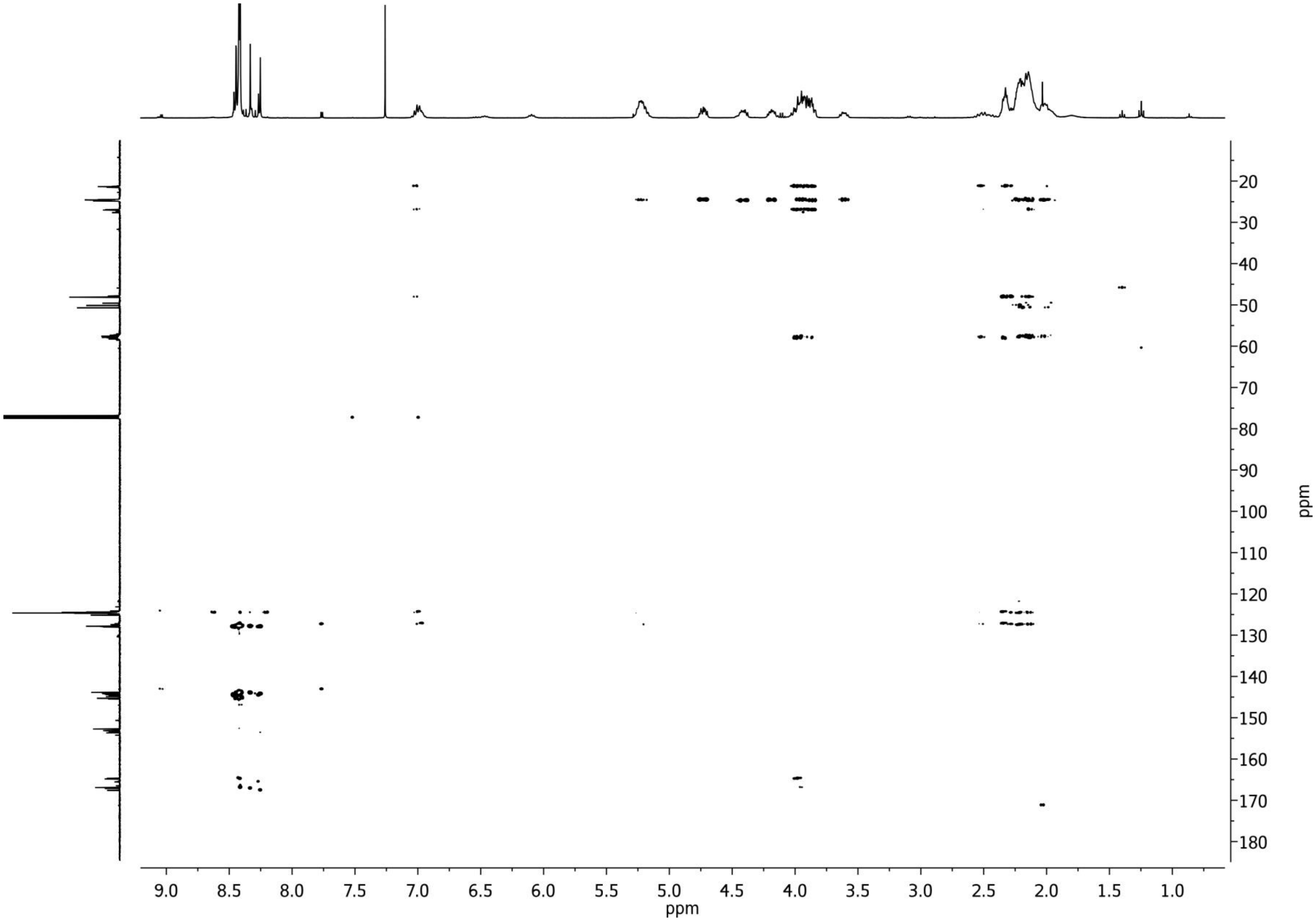

Figure S24. $^{1}H/^{13}C$ HMBC NMR spectra in $CDCl_3$ at 25ºC

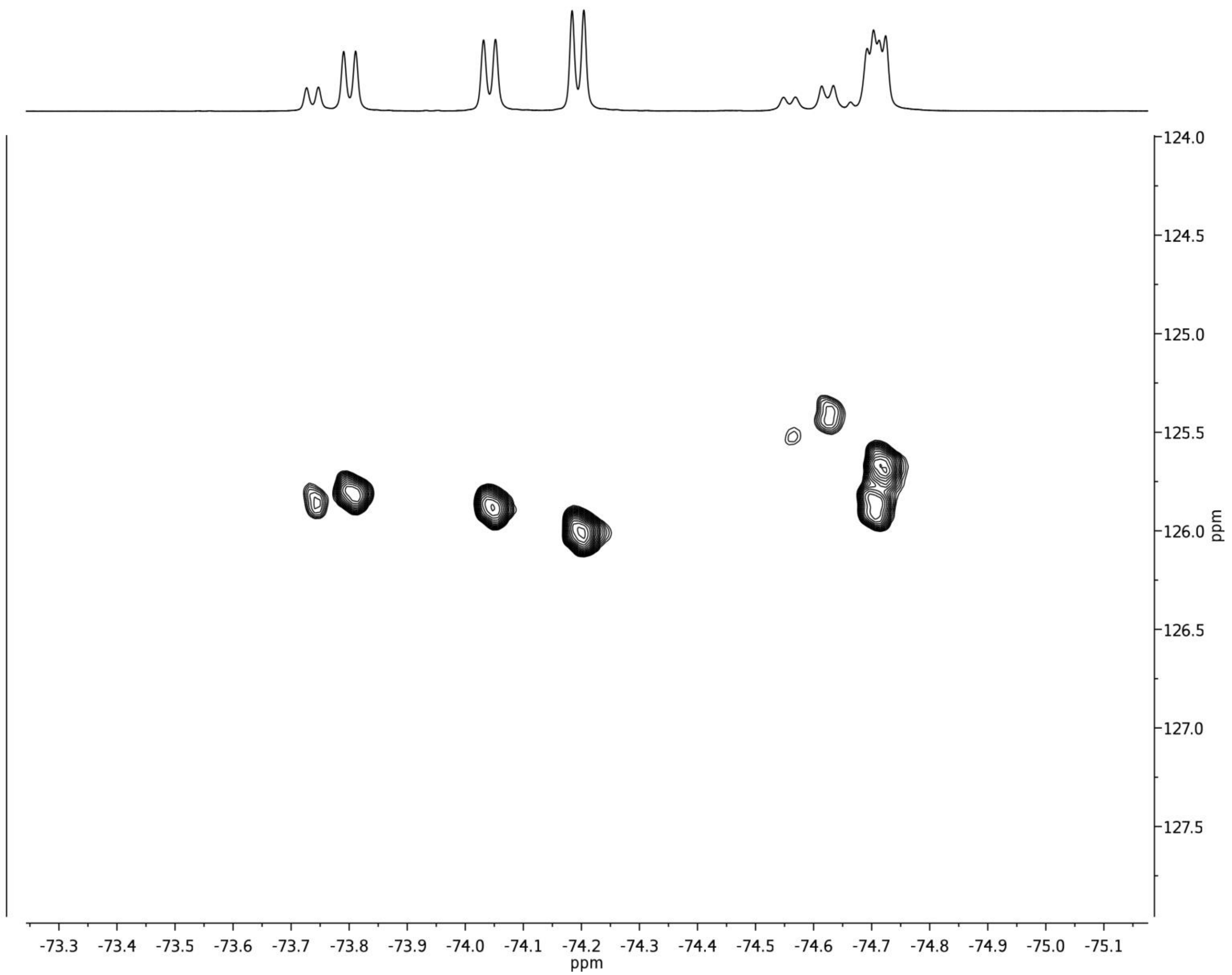

Figure S25. $^{19}F/^{13}C$-{^{19}F} HMBC NMR spectra in $CDCl_3$ at 25ºC

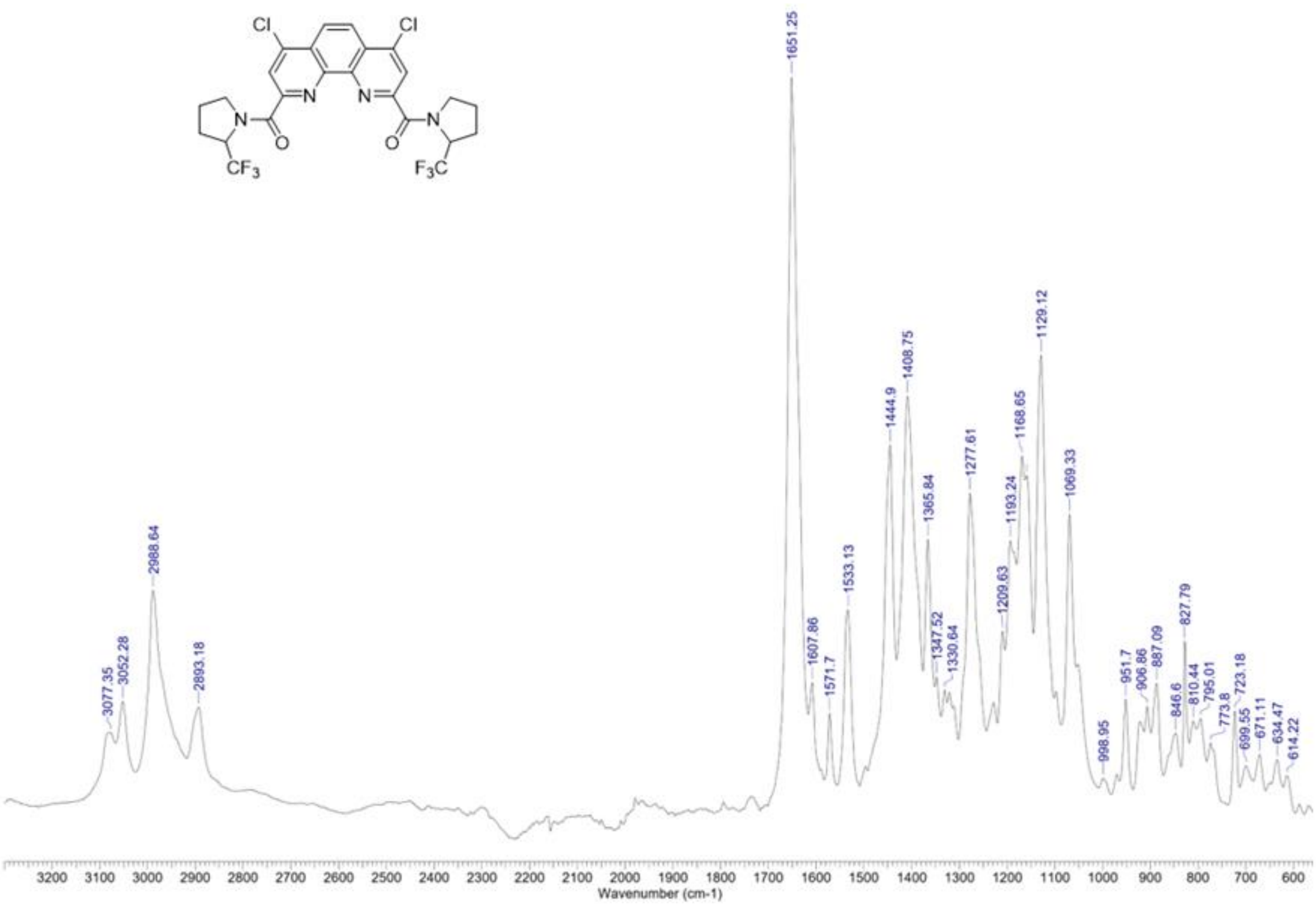

Figure S26. Solid-state IR spectra at 25ºC

Display Report

Analysis Info

Analysis Name D:\Data\Kolotyrkina\2021\Muzalevsky\0203040.d
Method tune_50-1600.m
Sample Name /MUSE PVS-103
Comment C24H18Cl2F6N4O2 calibrant added CH3OH

Acquisition Date 03.02.2021 19:33:55
Operator BDAL@DE
Instrument / Ser# micrOTOF 10248

Acquisition Parameter

Source Type	ESI	Ion Polarity	Positive	Set Nebulizer	1.0 Bar
Focus	Not active			Set Dry Heater	200 °C
Scan Begin	50 m/z	Set Capillary	4500 V	Set Dry Gas	4.0 l/min
Scan End	1600 m/z	Set End Plate Offset	-500 V	Set Divert Valve	Waste

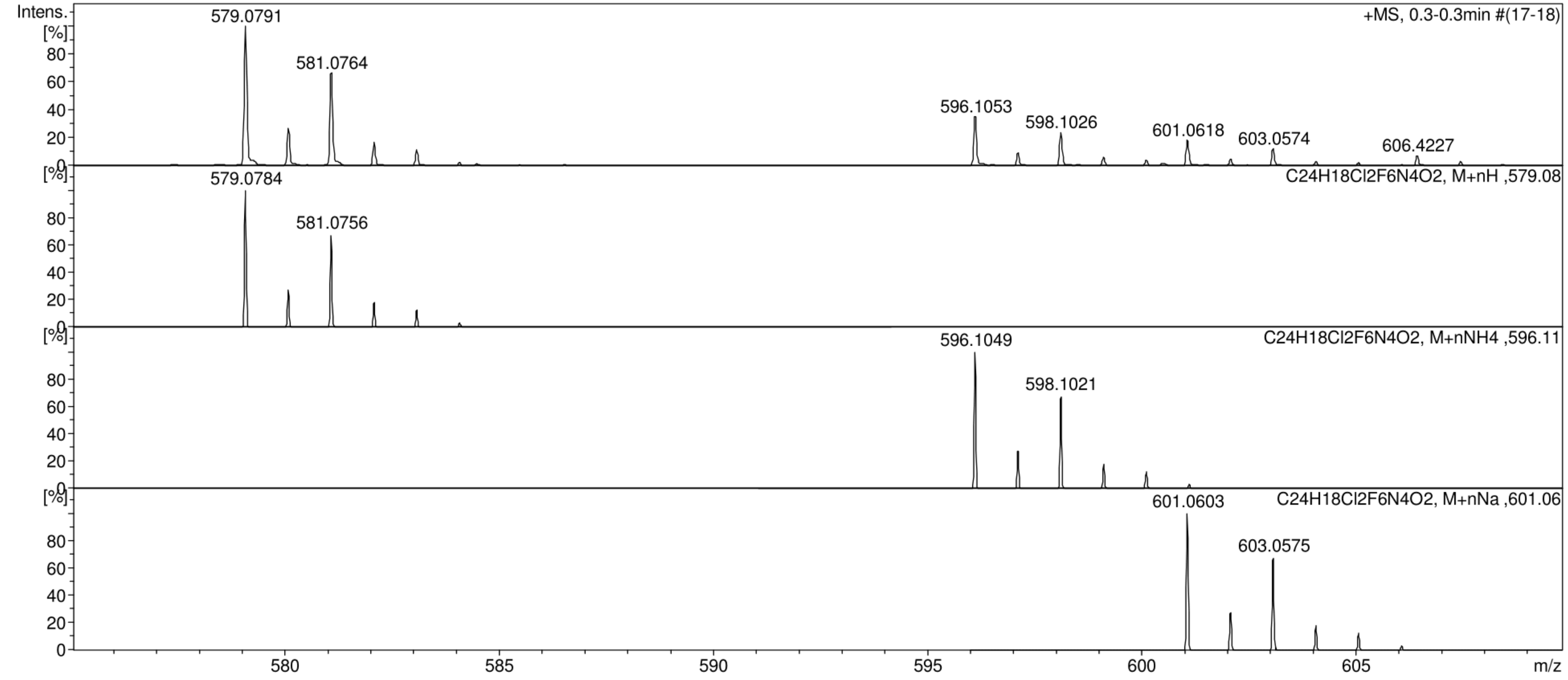

Figure S27. HRMS spectra of (**2**) at 25ºC

NMR and IR spectra of complexes

Complex 1*La(NO_3)$_3$

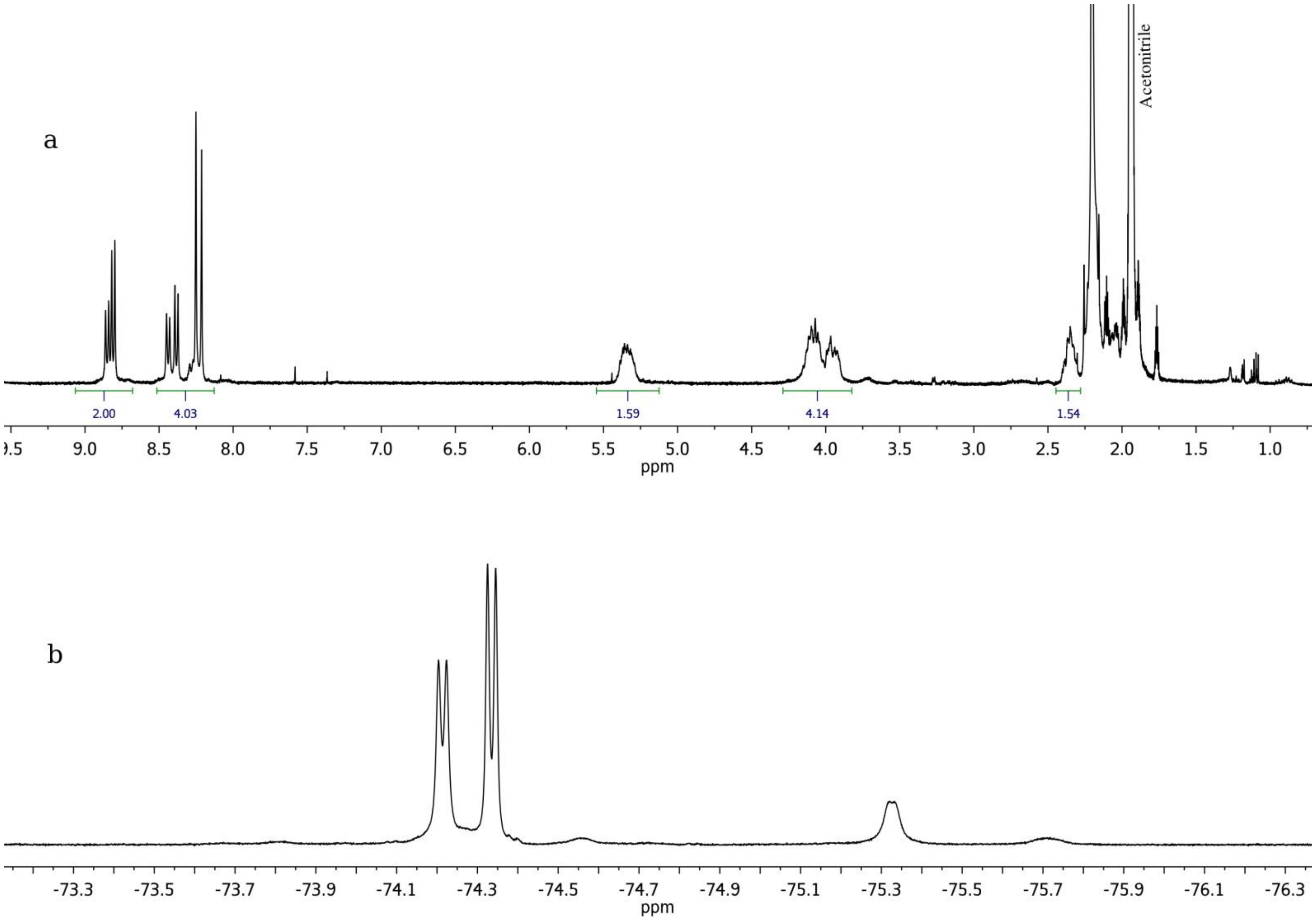

Figure S28. (a) ^{1}H and (b) ^{19}F NMR spectra in CD_3CN at 25ºC

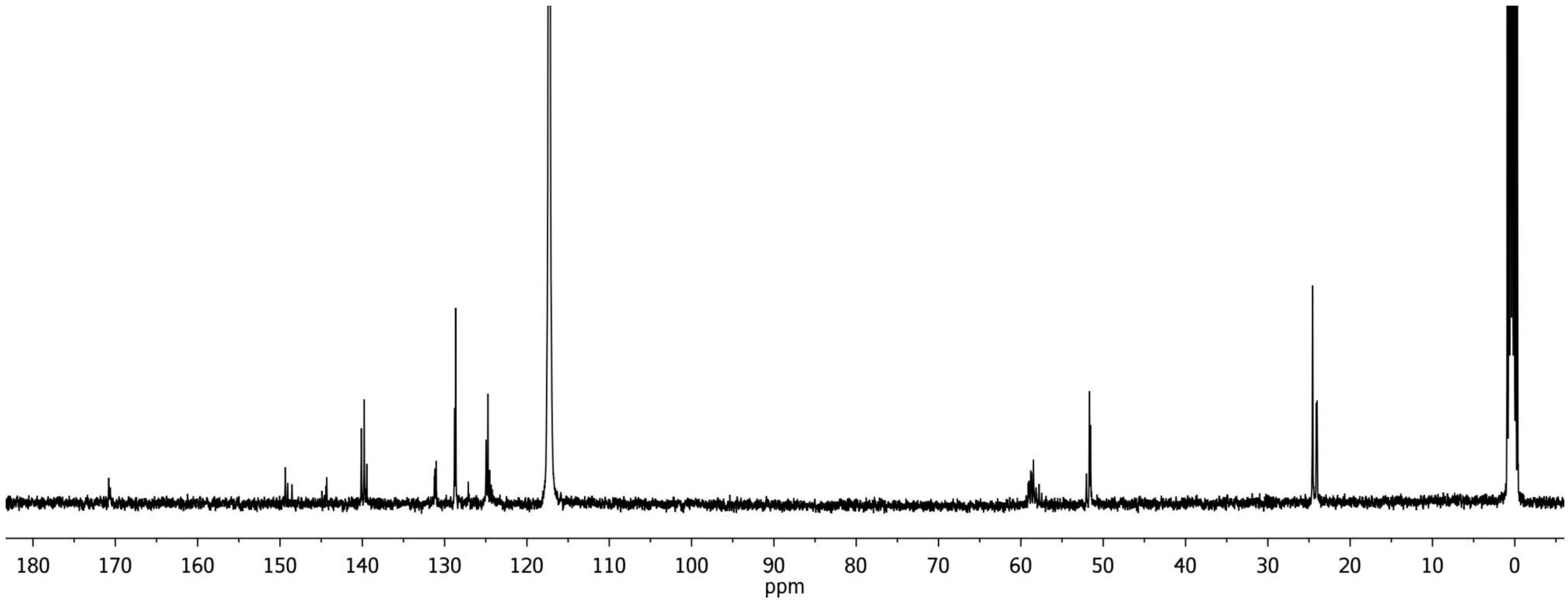

Figure S29. ^{13}C NMR spectra in CD_3CN at 25ºC

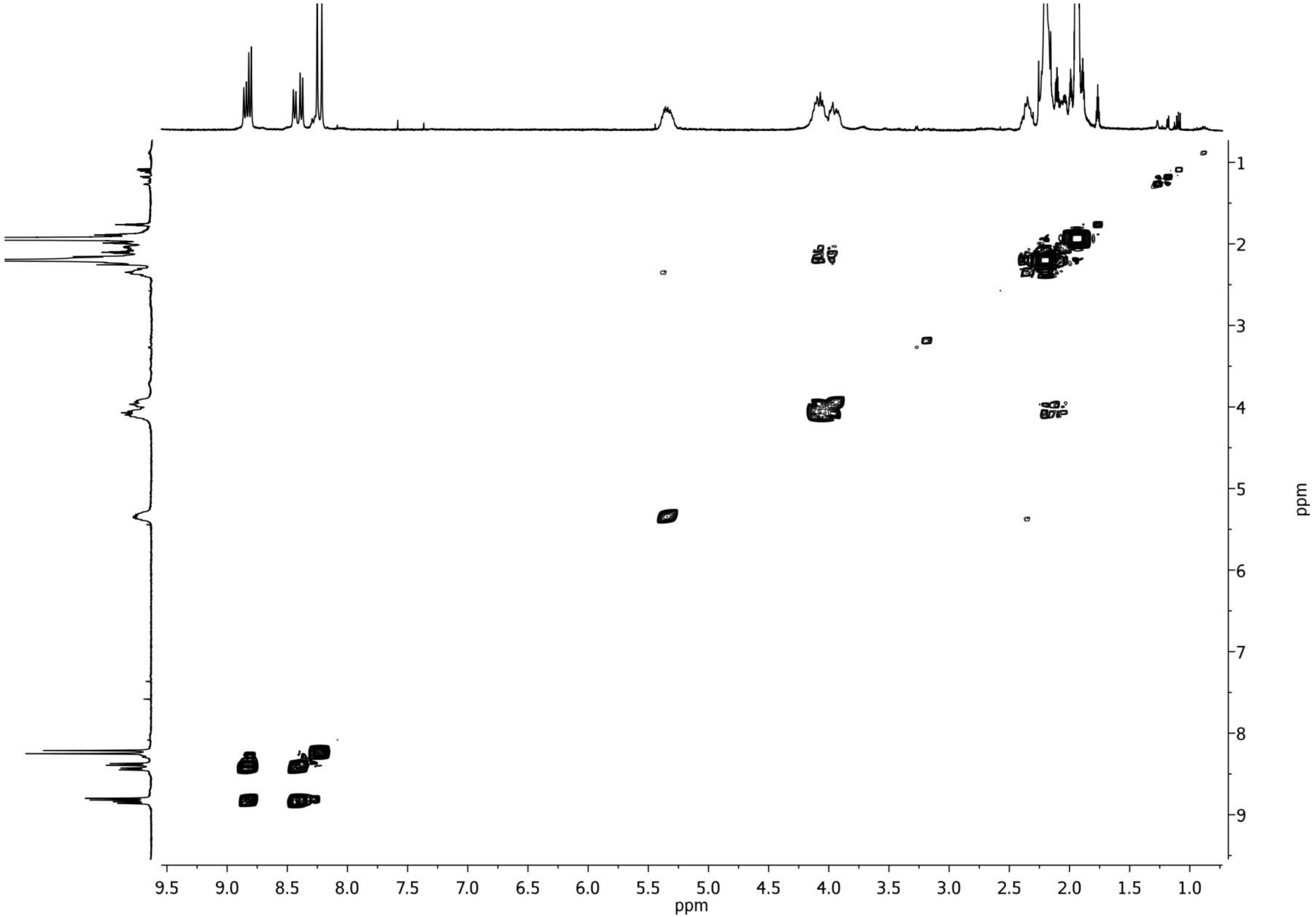

Figure S30. $^{1}H/^{1}H$ COSY NMR spectra in CD_3CN at 25ºC

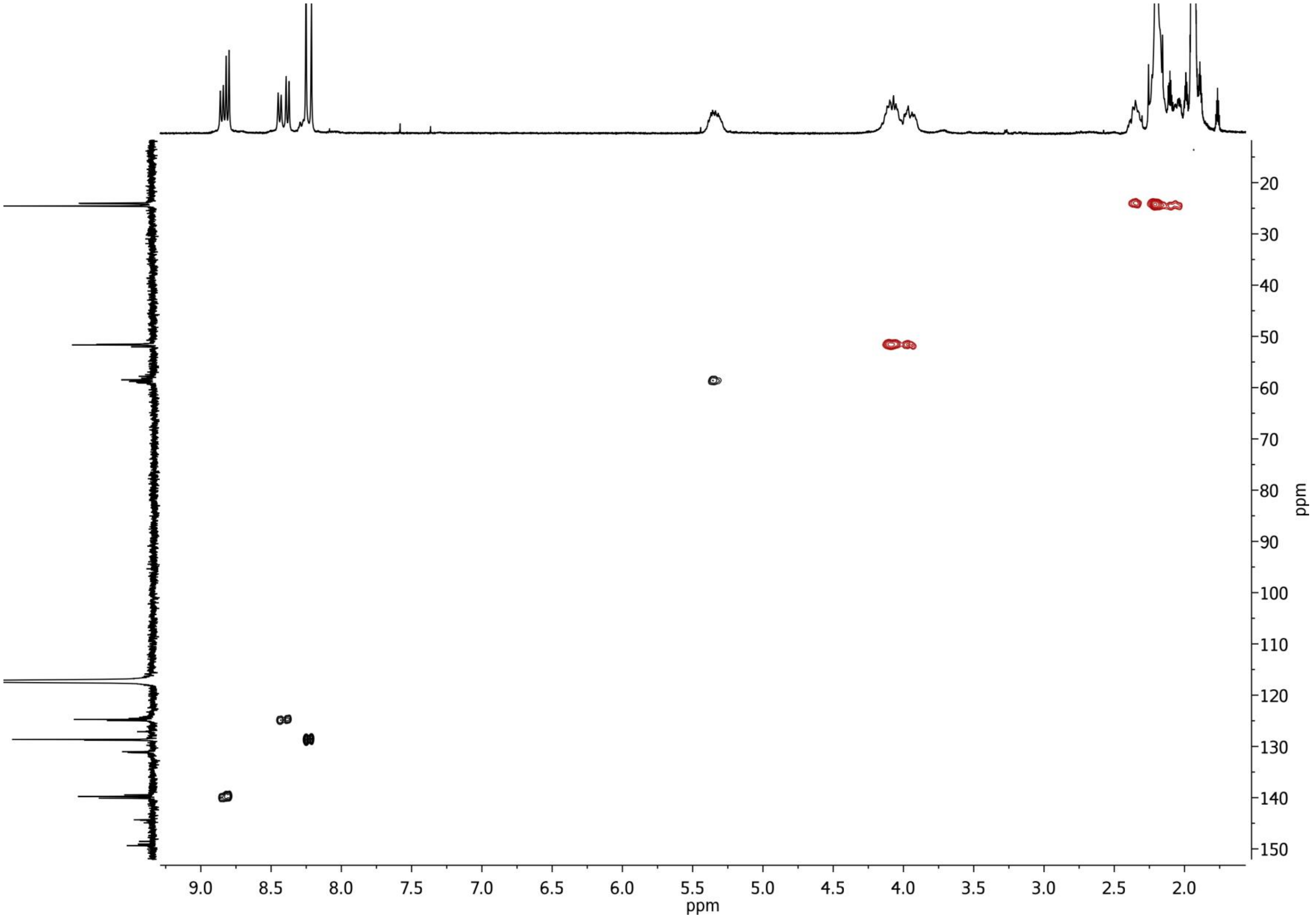

Figure S31. $^{1}H/^{13}C$ HSQC NMR spectra in CD_3CN at 25ºC

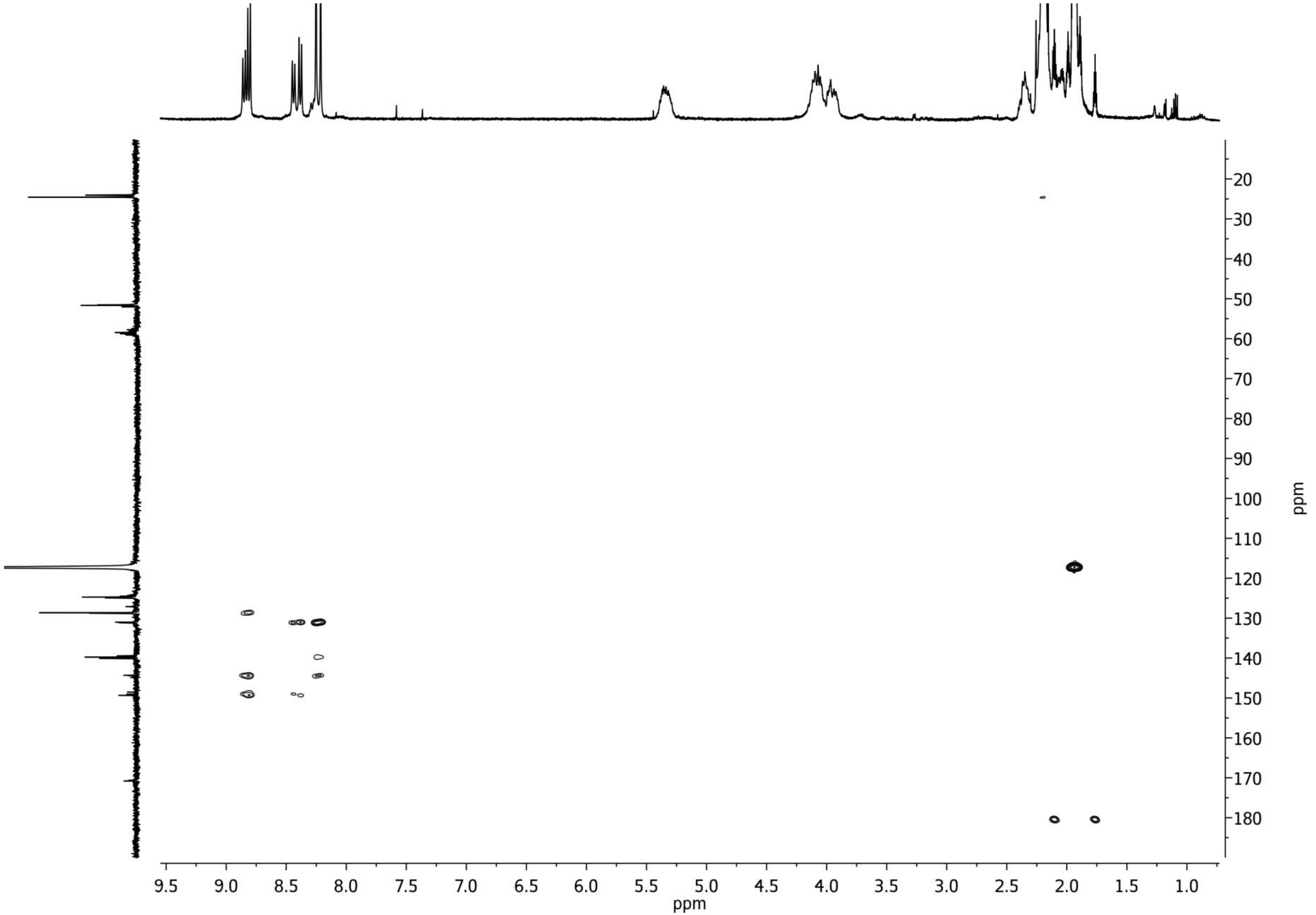

Figure S32. $^{1}H/^{13}C$ HMBC NMR spectra in CD_3CN at 25ºC

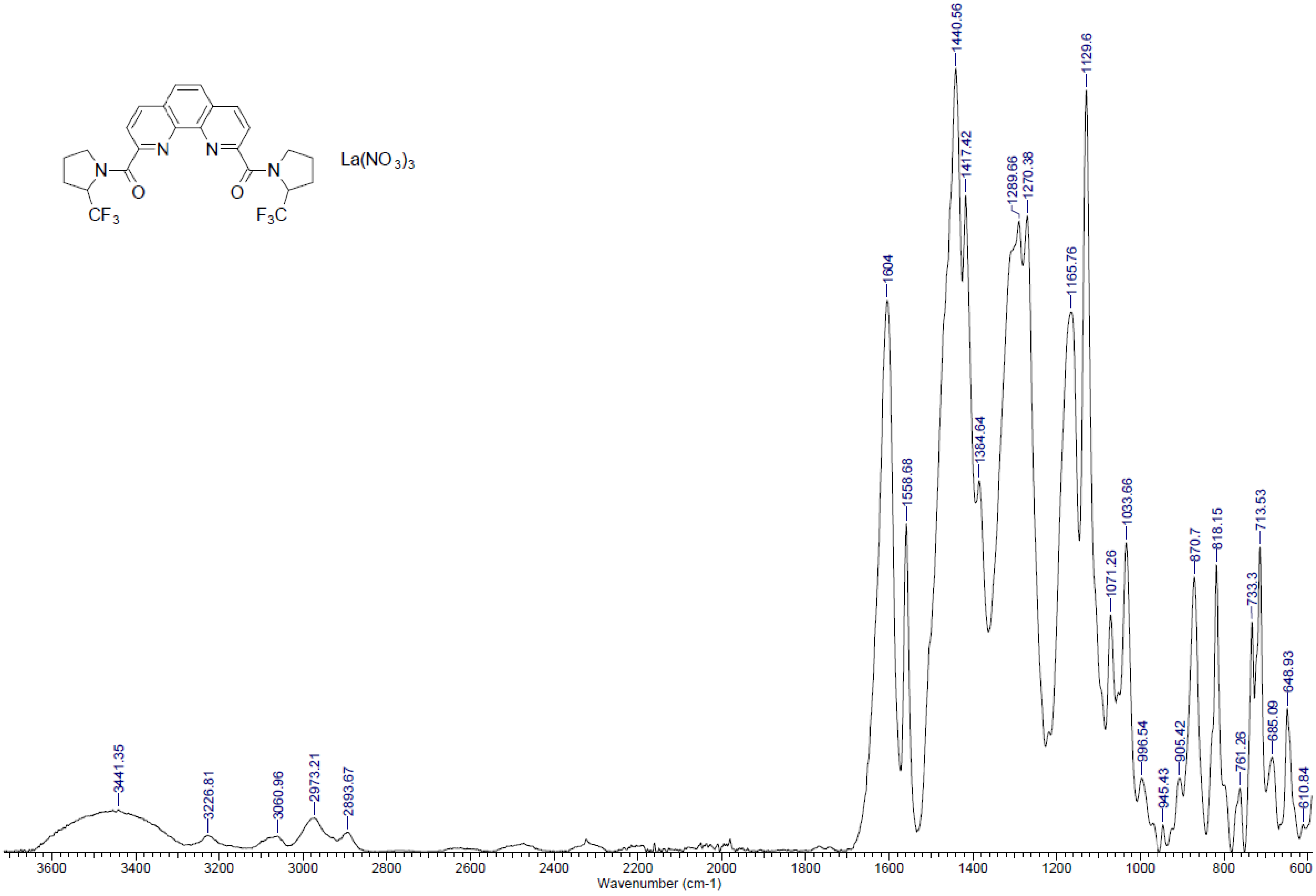

Figure S33. Solid-state IR spectra at 25ºC

Complex 1*Nd(NO_3)$_3$

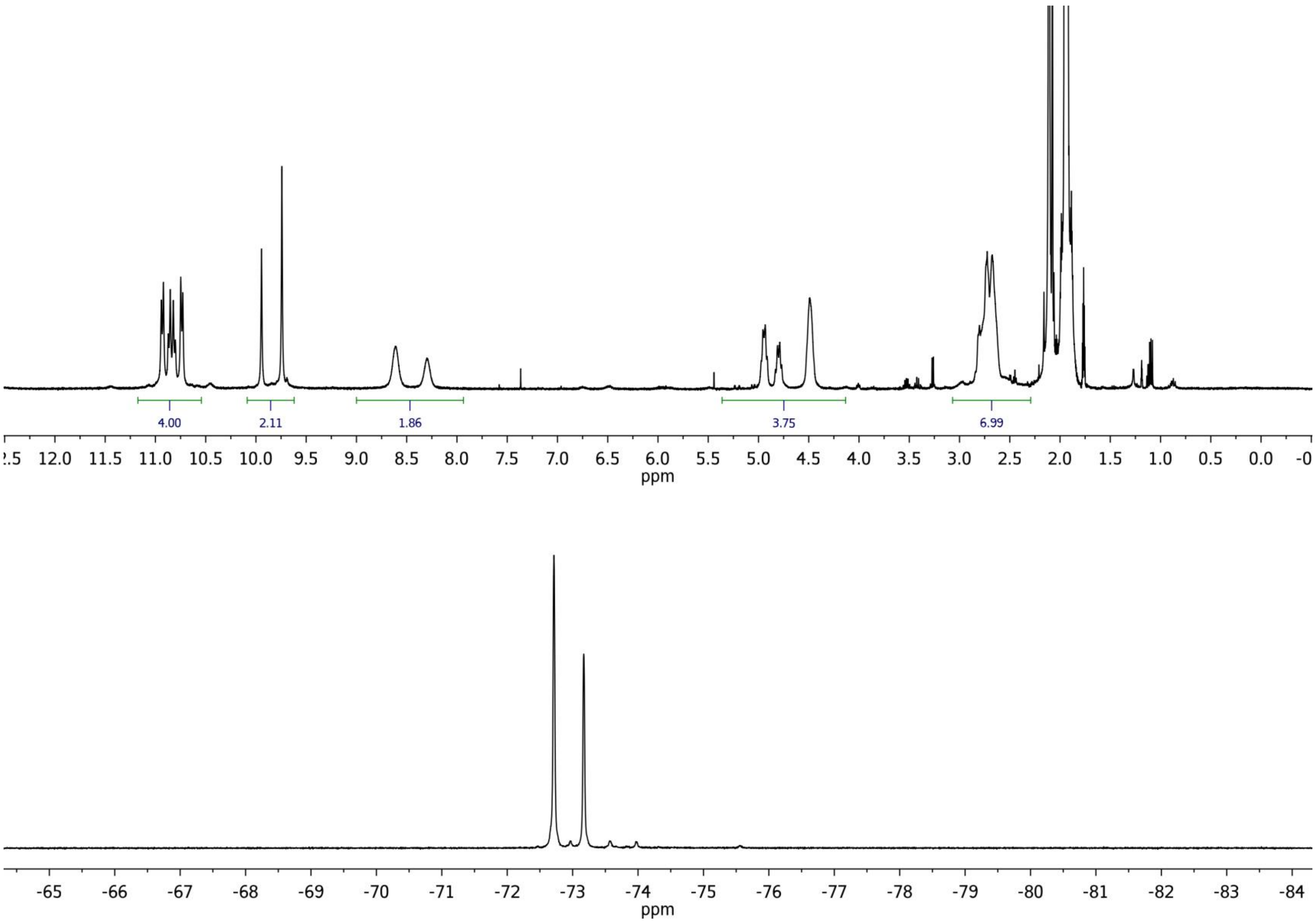

Figure S34. (a) ^{1}H and (b) ^{19}F NMR spectra in CD_3CN at 25ºC

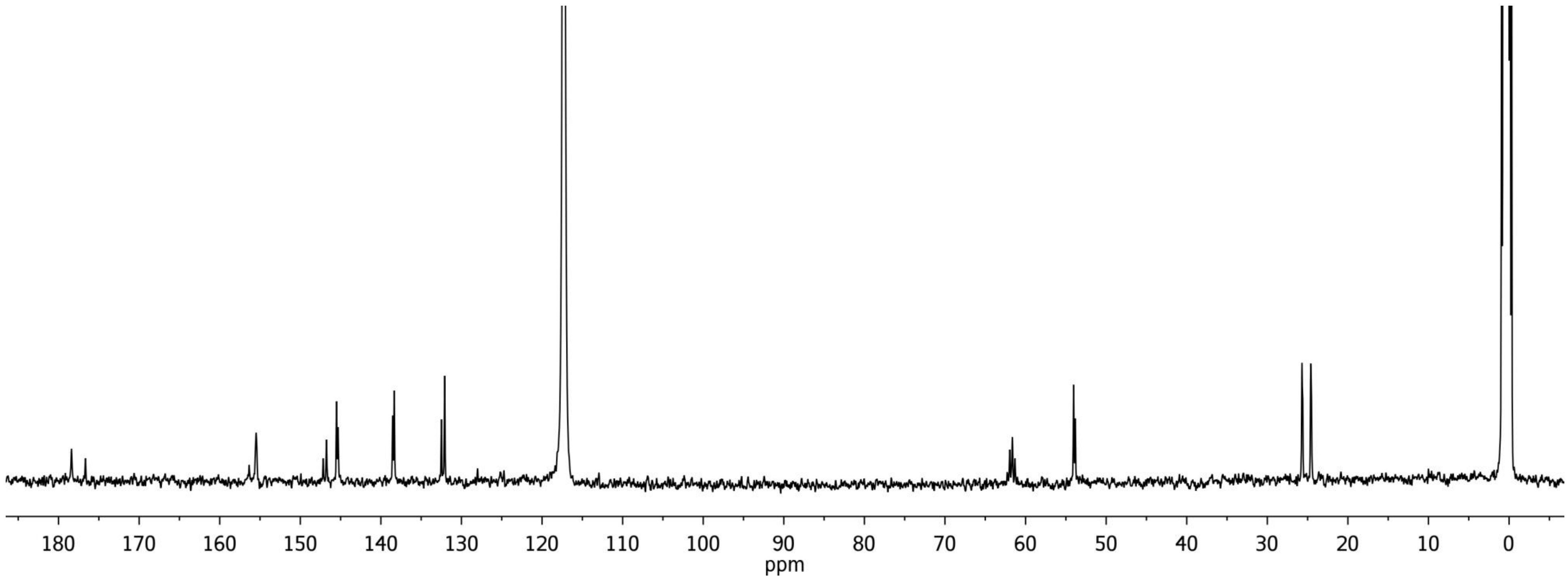

Figure S35. ^{13}C NMR spectra in CD_3CN at 25ºC

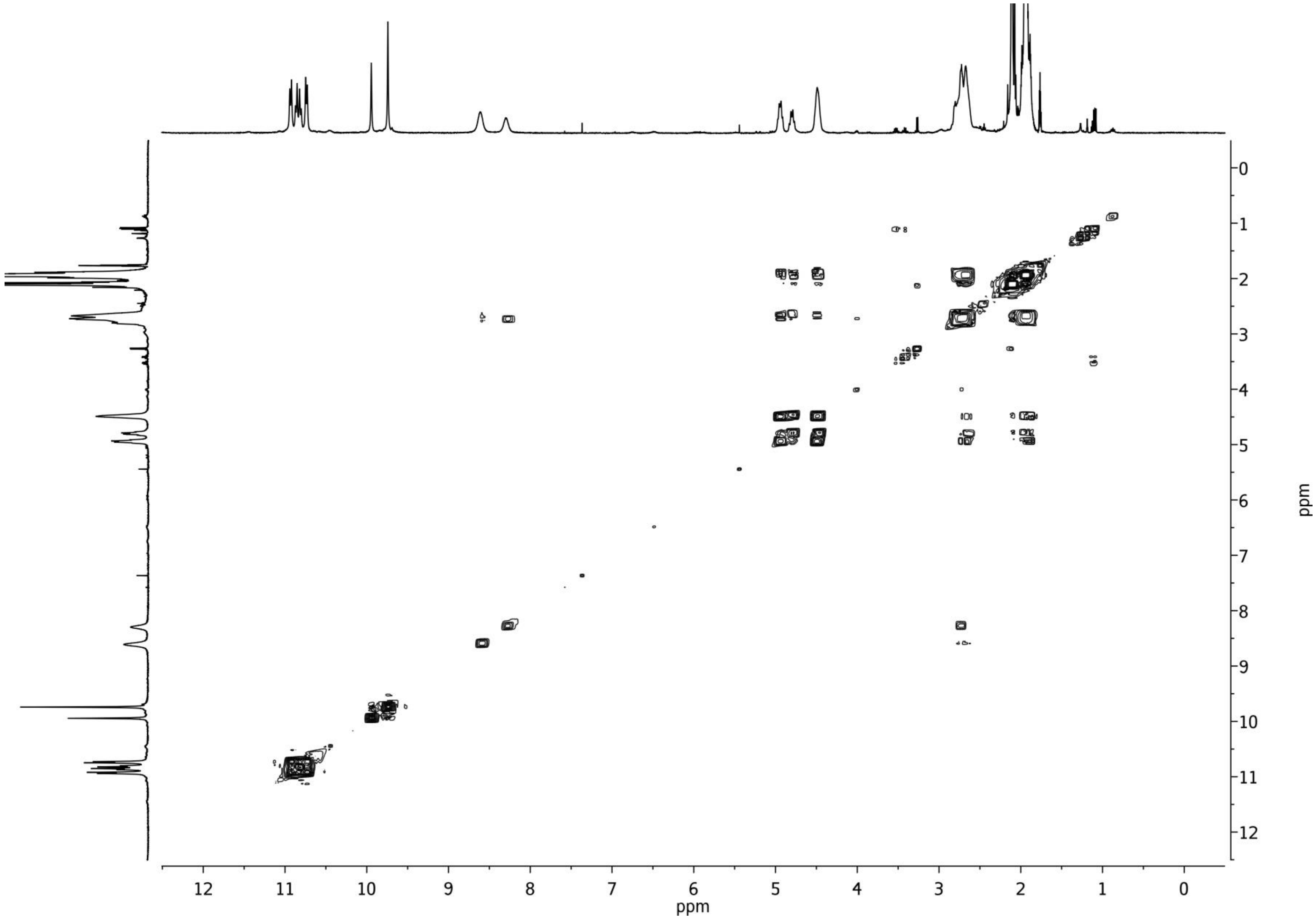

Figure S36. $^{1}H/^{1}H$ COSY NMR spectra in CD_3CN at 25ºC

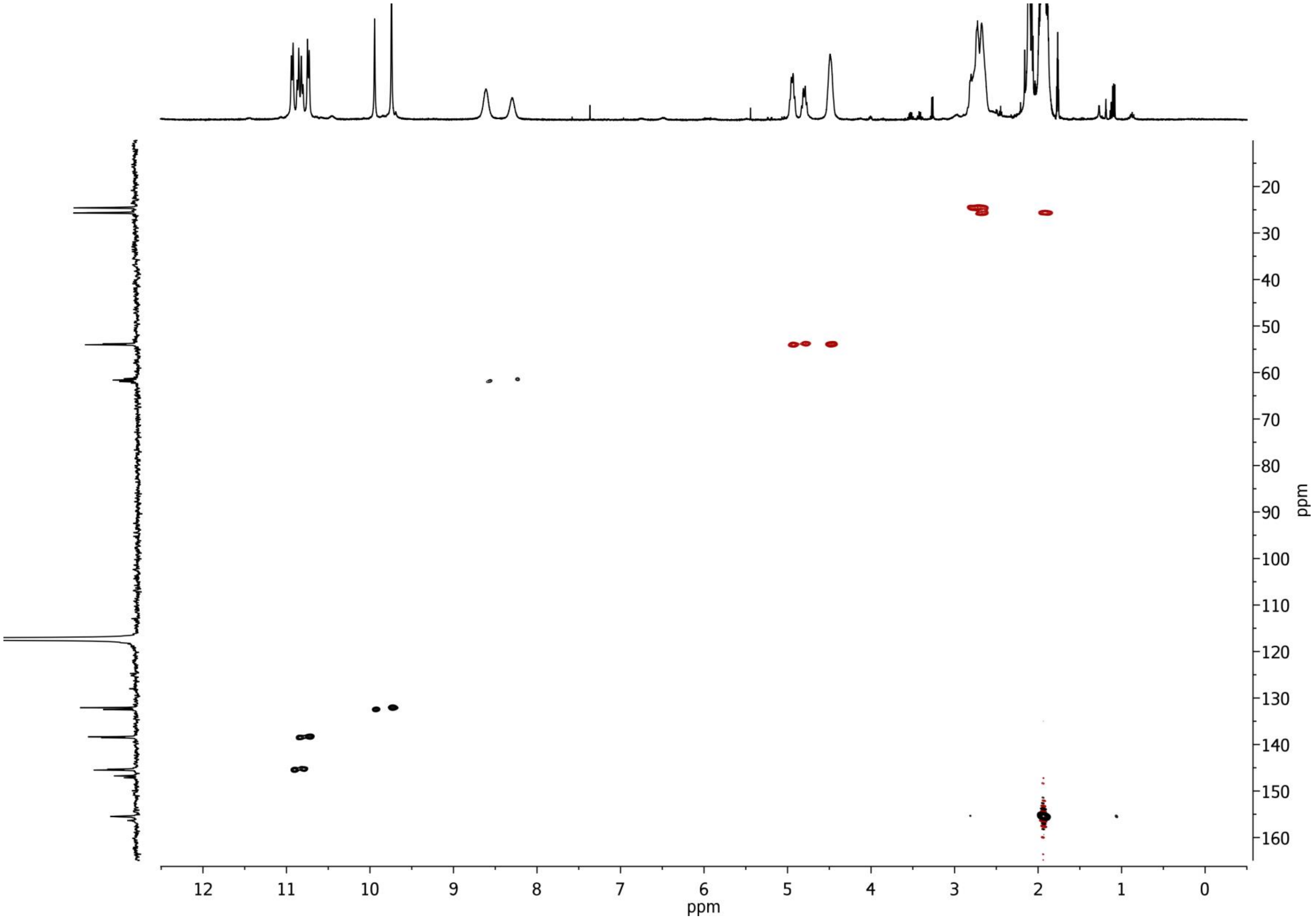

Figure S37. $^{1}H/^{13}C$ HSQC NMR spectra in CD_3CN at 25ºC

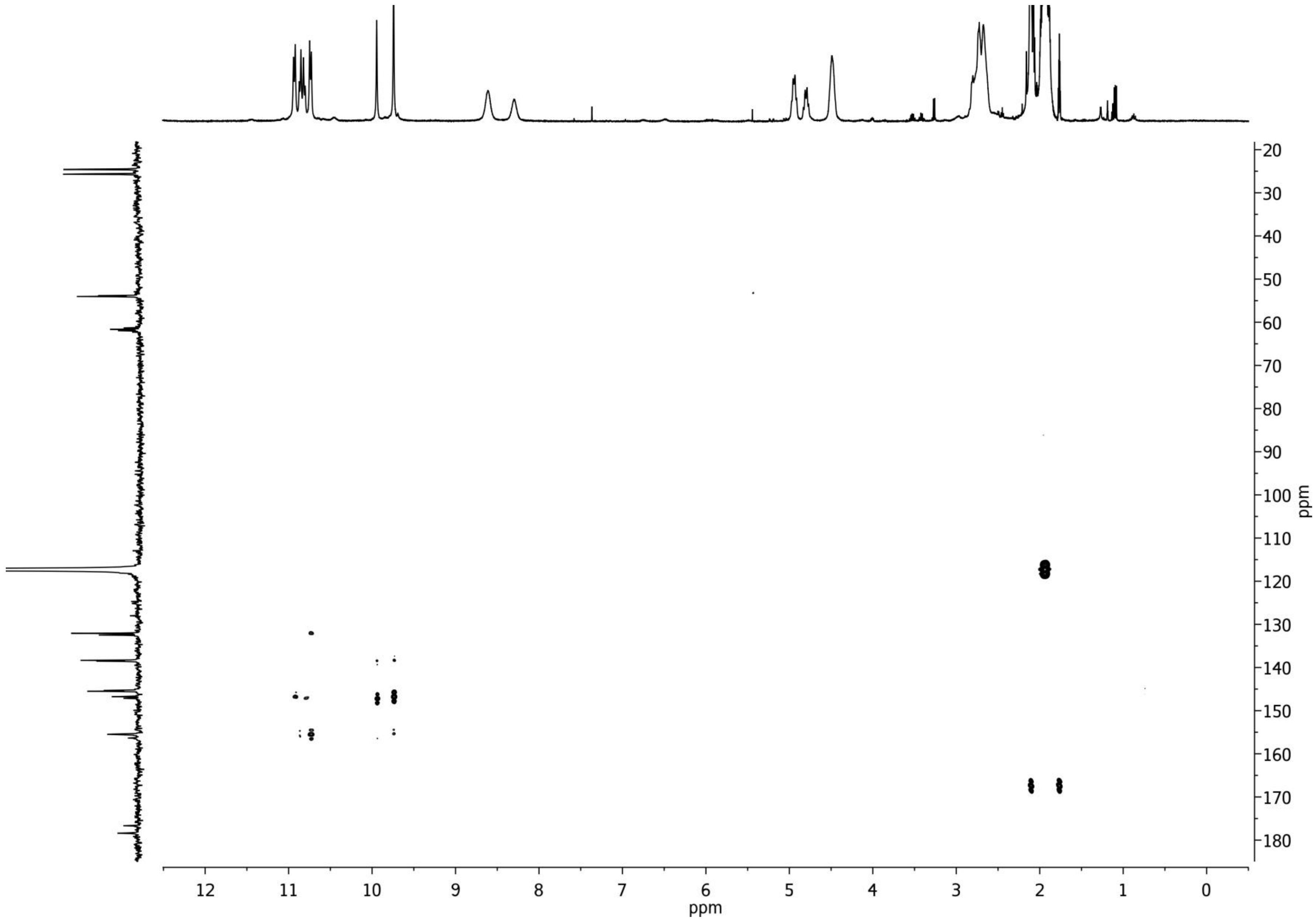

Figure S38. $^{1}H/^{13}C$ HMBC NMR spectra in CD_3CN at 25ºC

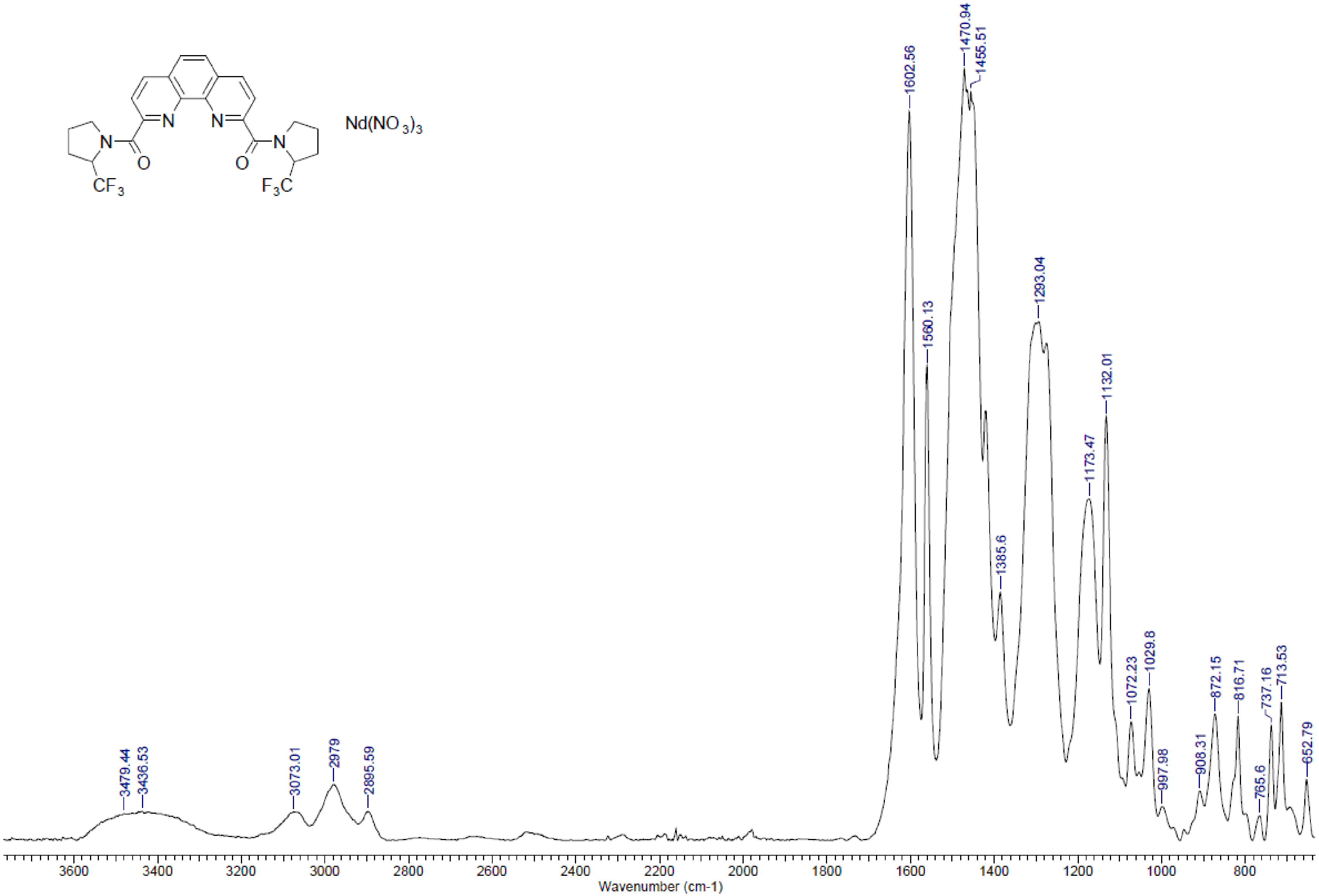

Figure S39. Solid-state IR spectra at 25ºC

Complex 1*Eu(NO_3)$_3$

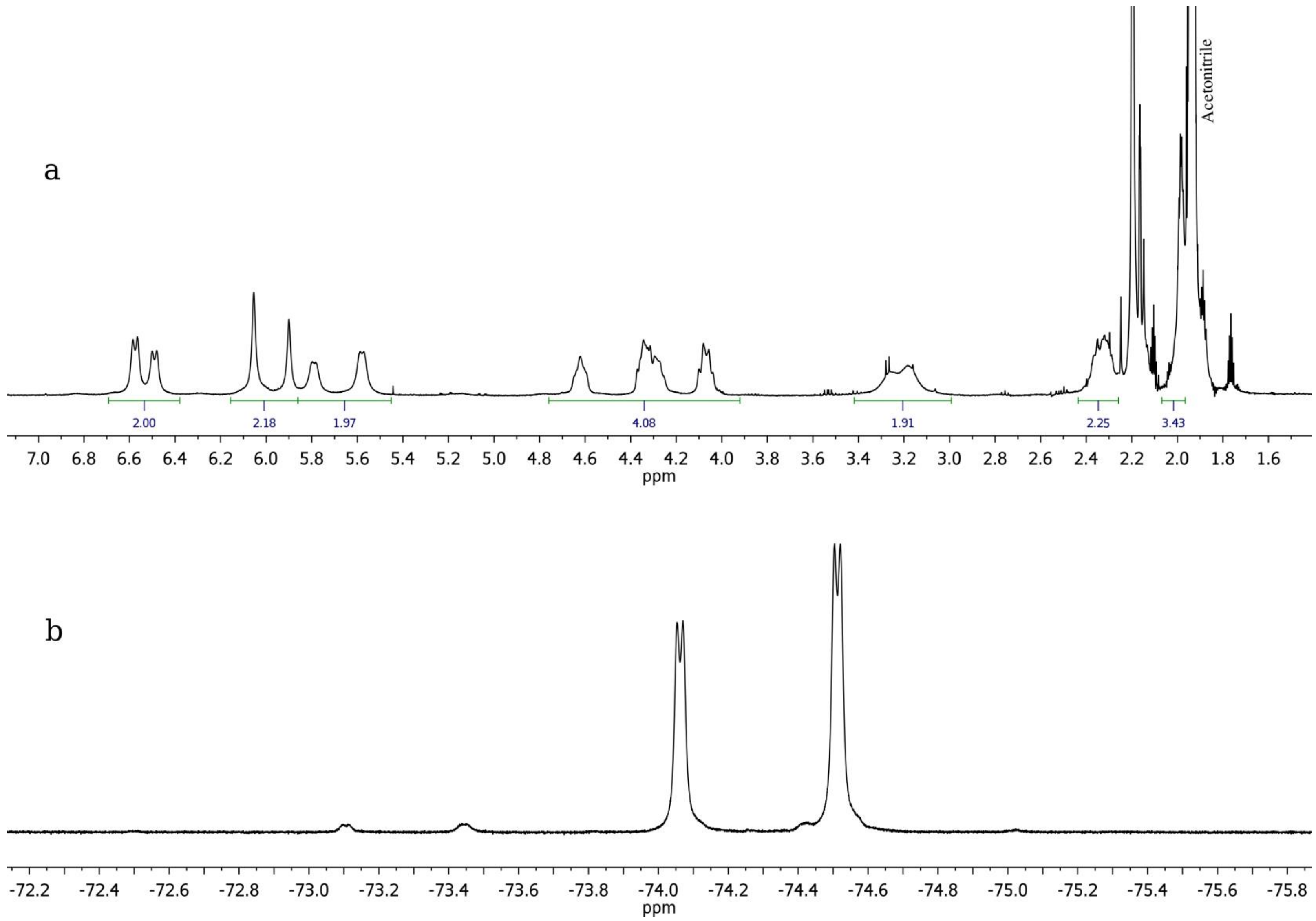

Figure S40. (a) ^{1}H and (b) ^{19}F NMR spectra in CD_3CN at 25ºC

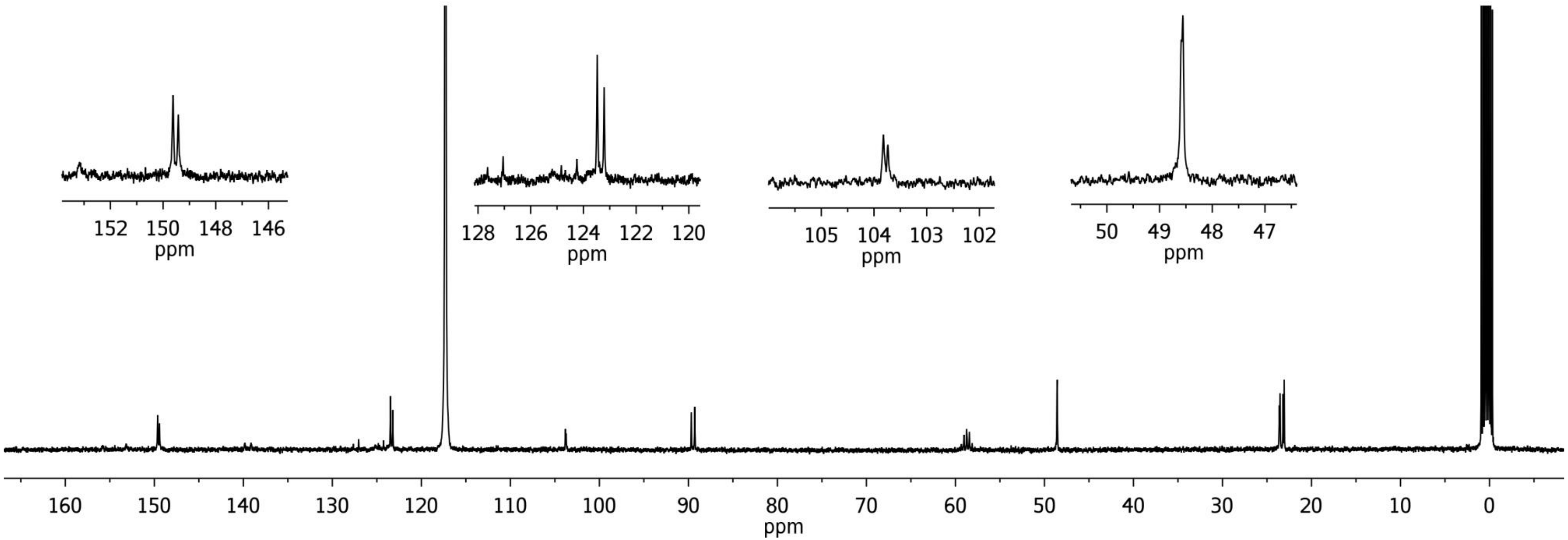

Figure S41. ^{13}C NMR spectra in CD_3CN at 25ºC

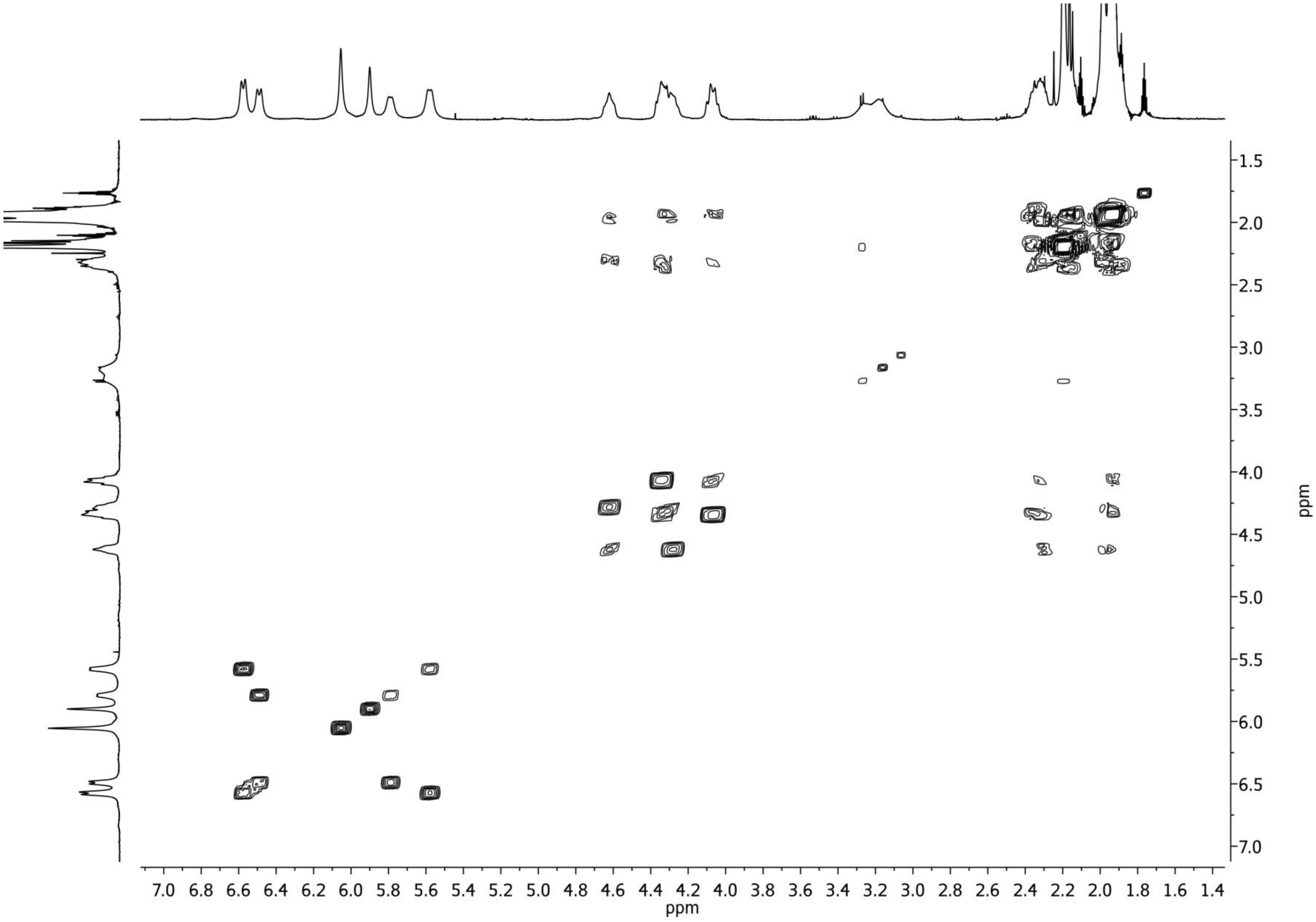

Figure S42. $^1H/^1H$ COSY NMR spectra in CD_3CN at 25ºC

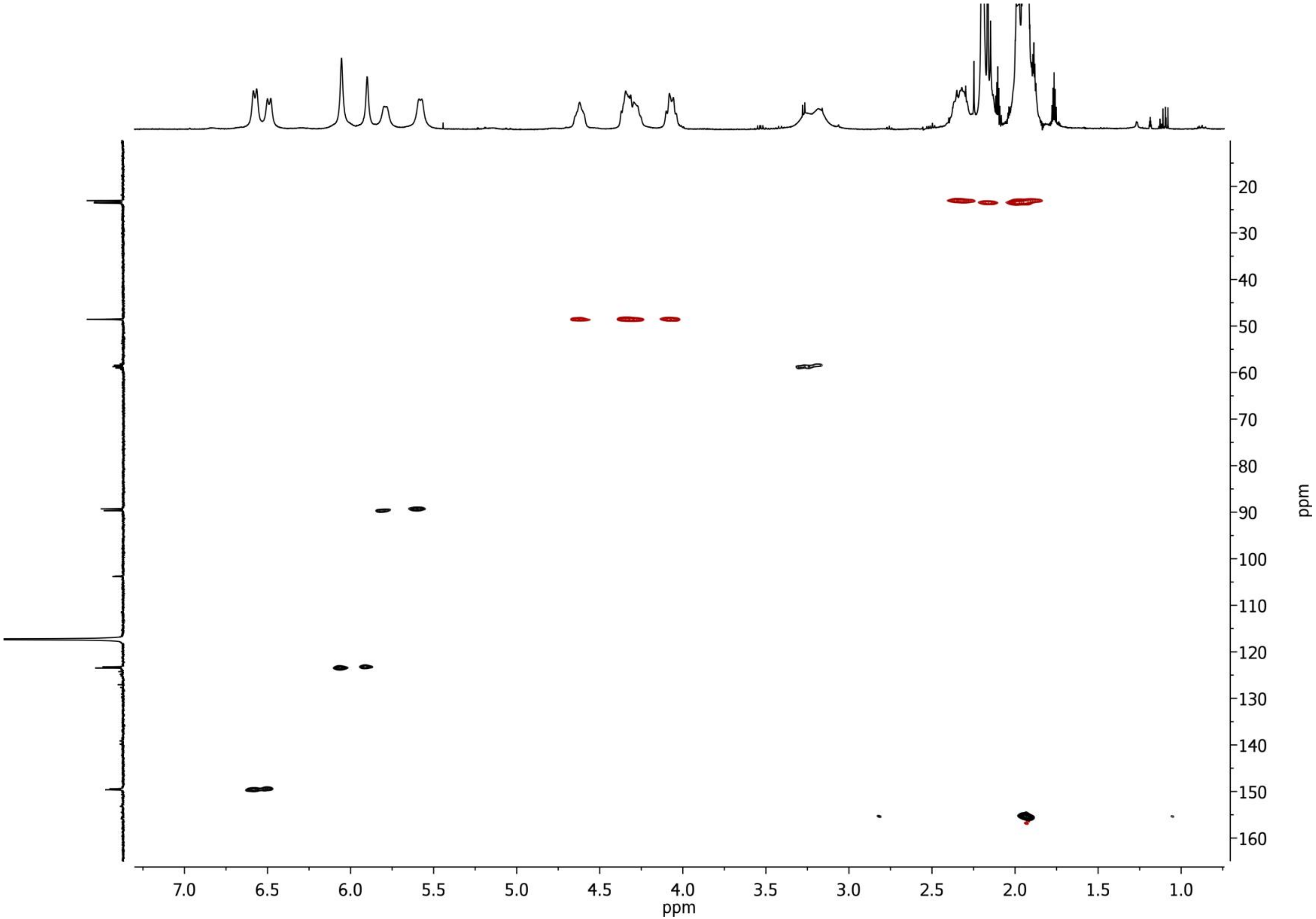

Figure S43. $^{1}H/^{13}C$ HSQC NMR spectra in CD_3CN at 25ºC

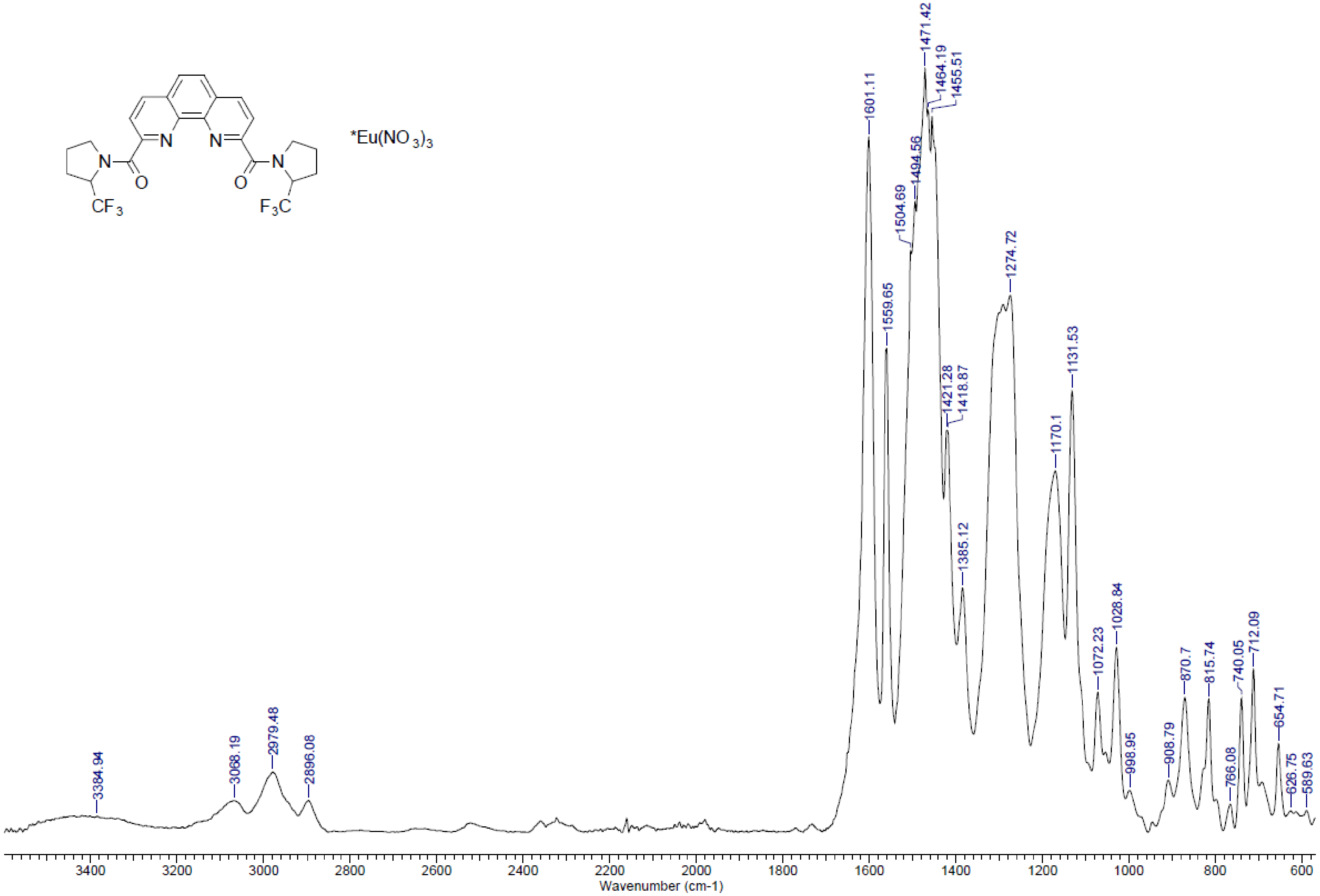

Figure S44. Solid-state IR spectra at 25ºC

Complex 1*Lu(NO_3)$_3$

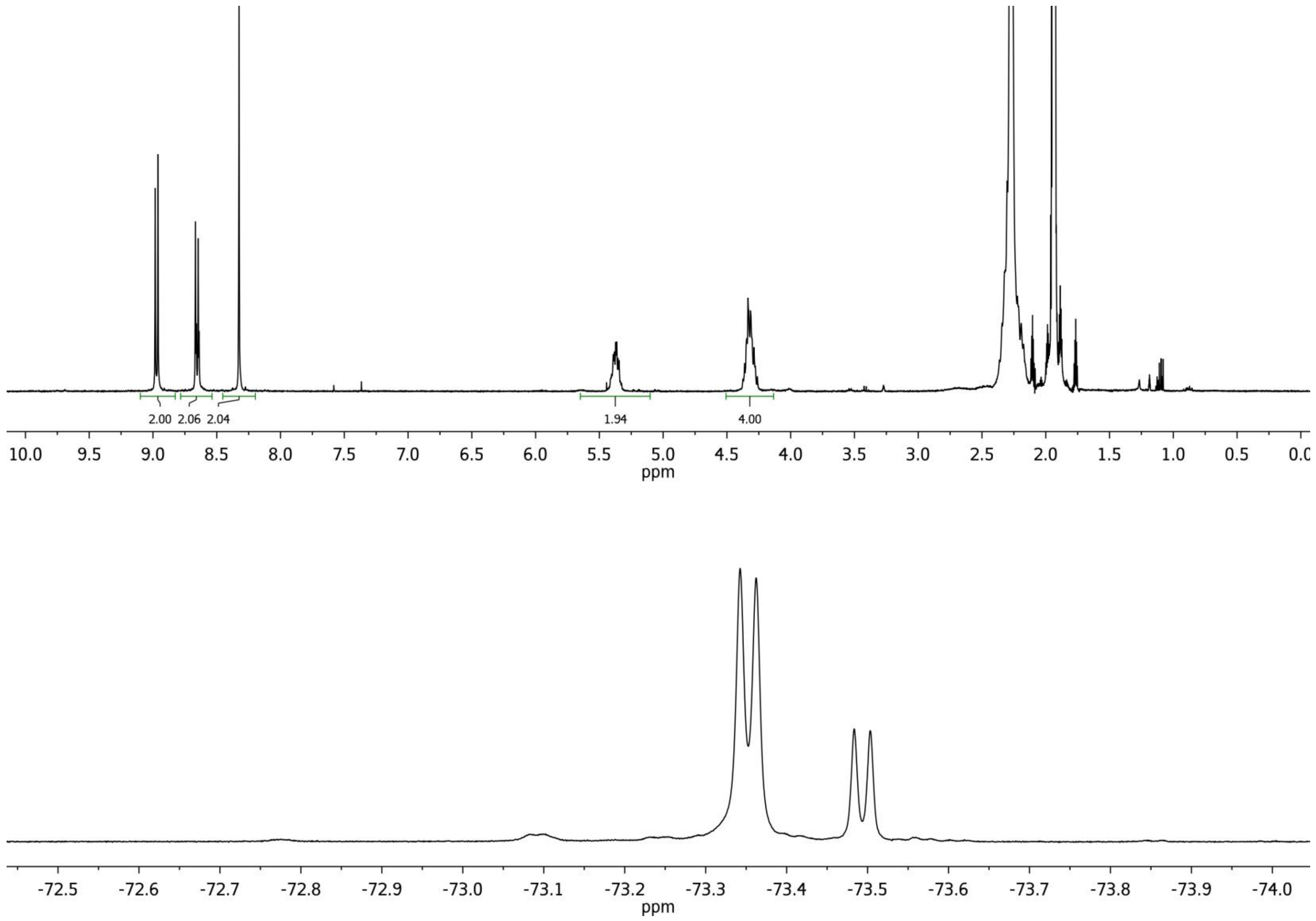

Figure S45. (a) ^{1}H and (b) ^{19}F NMR spectra in CD_3CN at 25ºC

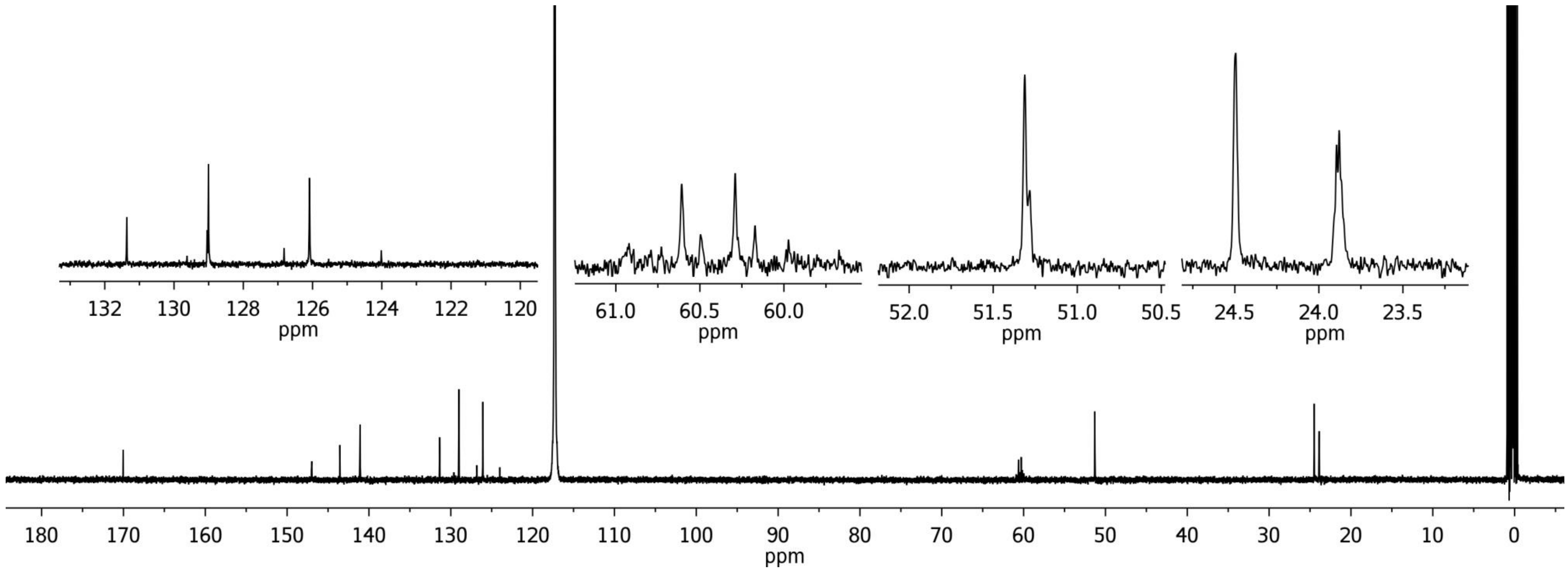

Figure S46. ^{13}C NMR spectra in CD_3CN at 25ºC

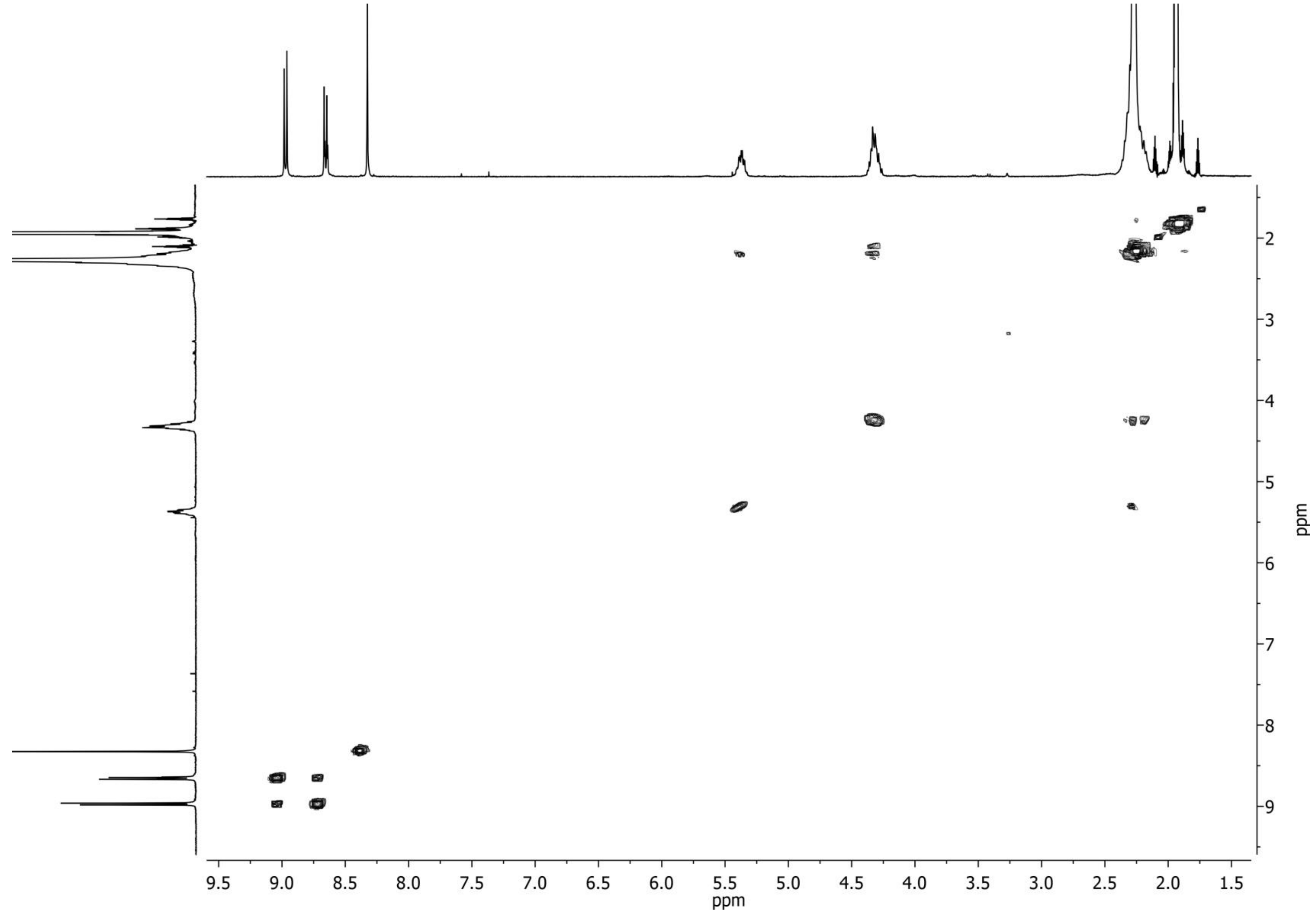

Figure S47. $^{1}H/^{1}H$ COSY NMR spectra in CD_3CN at 25ºC

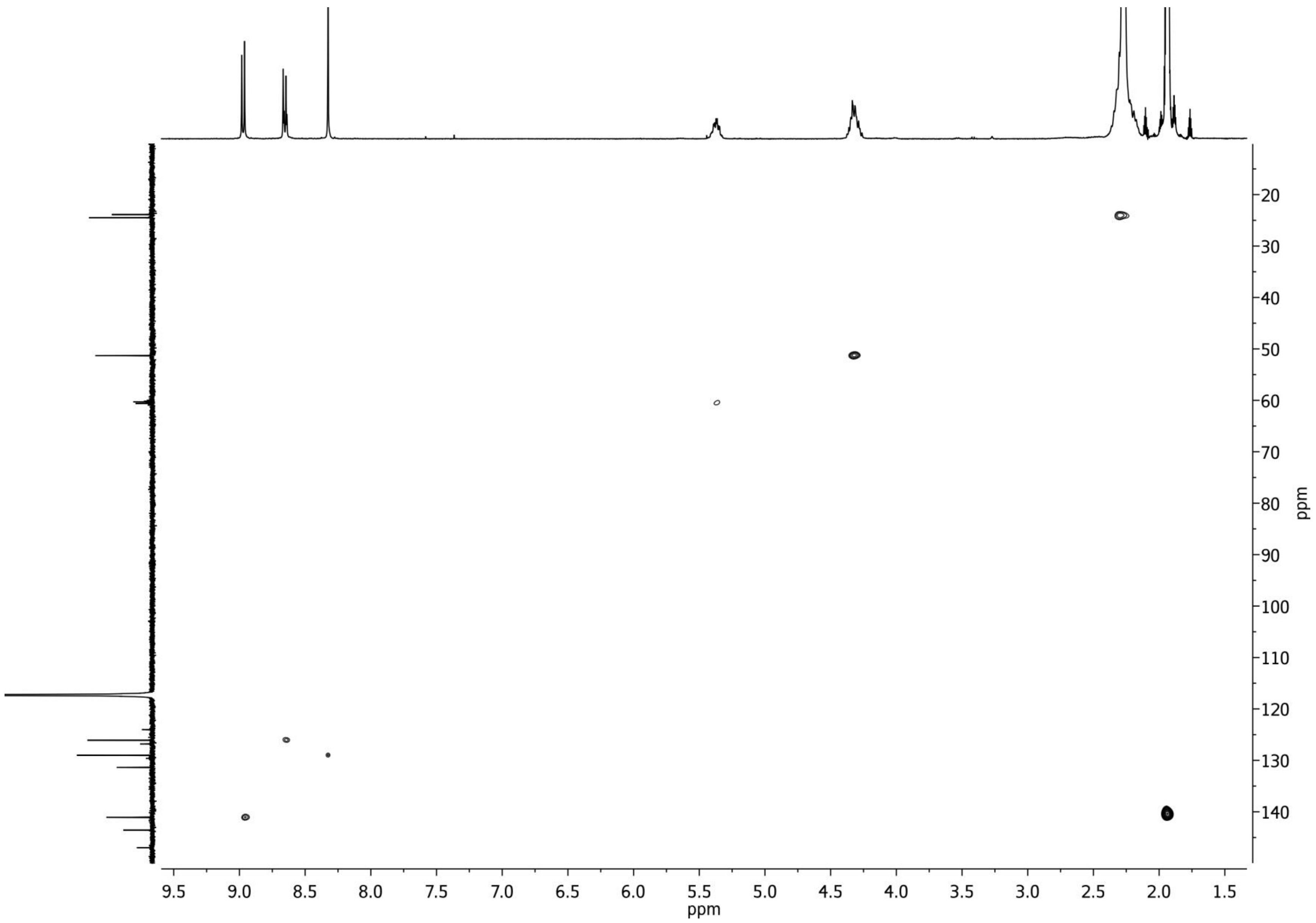

Figure S48. $^{1}H/^{13}C$ HSQC NMR spectra in CD_3CN at 25ºC

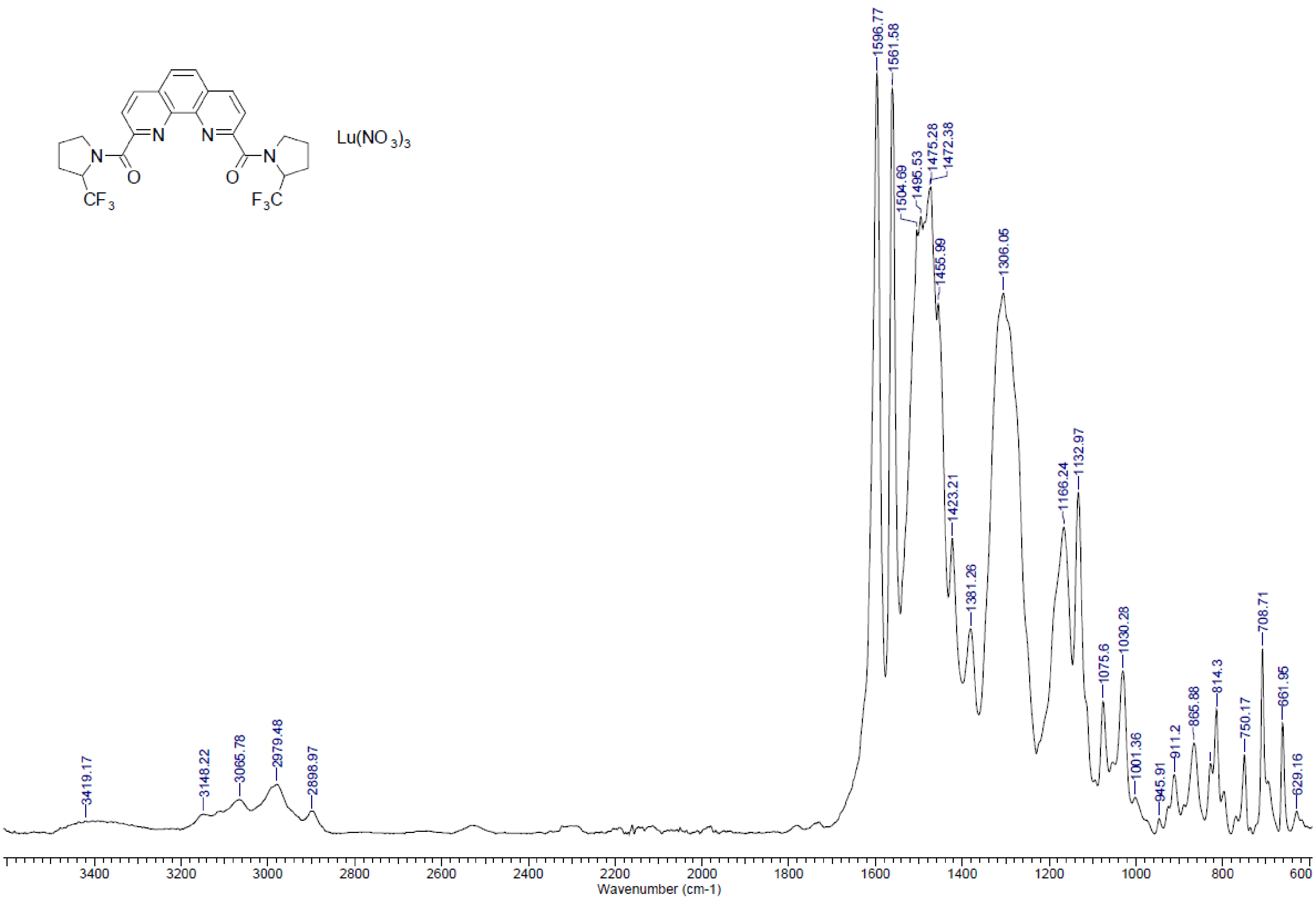

Figure S49. Solid-state IR spectra at 25ºC

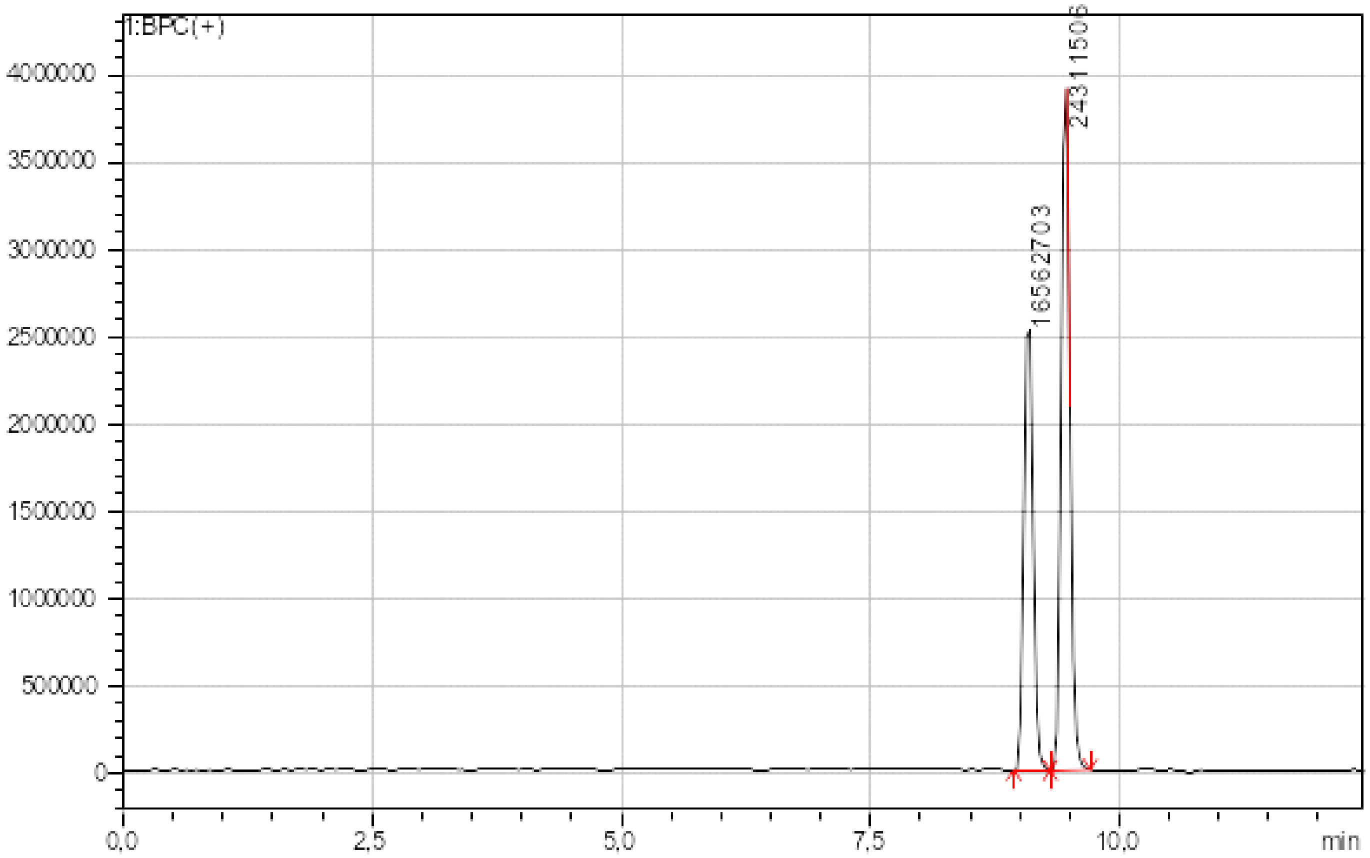

Figure S50. HPLC chromatogram of ligand **1** isomers.

2. Spectrophotometric titration data

Ligand 1

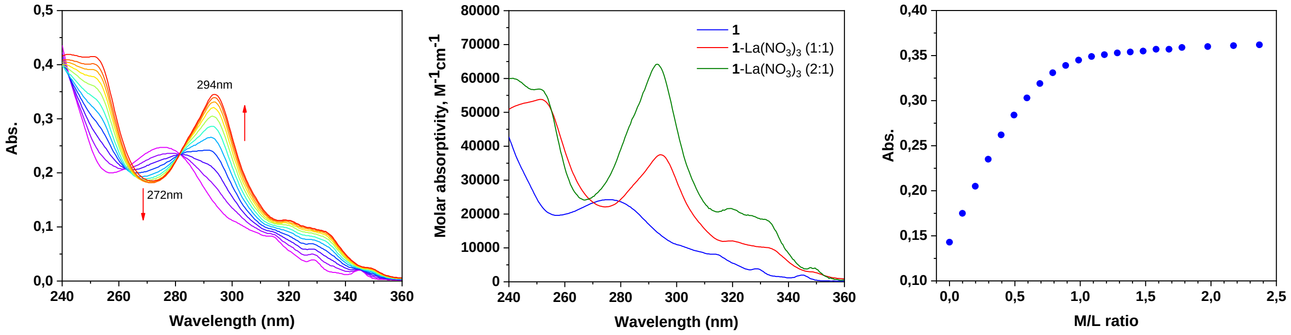

Figure S51. Spectrophotometric titration of **1** (ca. 10^{-5} mol/L) with La^{3+} ions (ca. 10^{-3} mol/L) in CH_3CN solution (T = 25.0 ± 0.1 °C, I = 0 M, V_0 = 2.0 mL). Left: absorption spectra. Middle: molar absorptivities of free ligand **1** and La(III) complexes calculated from spectral deconvolution. Right: titration curve at 294 nm (maximum absorption).

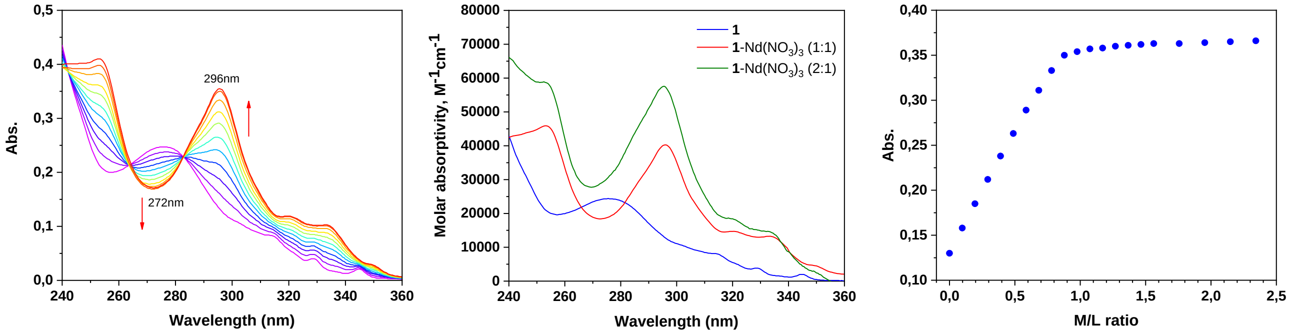

Figure S52. Spectrophotometric titration of **1** (ca. 10^{-5} mol/L) with Nd^{3+} ions (ca. 10^{-3} mol/L) in CH_3CN solution (T = 25.0 ± 0.1 °C, I = 0 M, V_0 = 2.0 mL). Left: absorption spectra. Middle: molar absorptivities of free ligand **1** and Nd(III) complexes calculated from spectral deconvolution. Right: titration curve at 296 nm (maximum absorption).

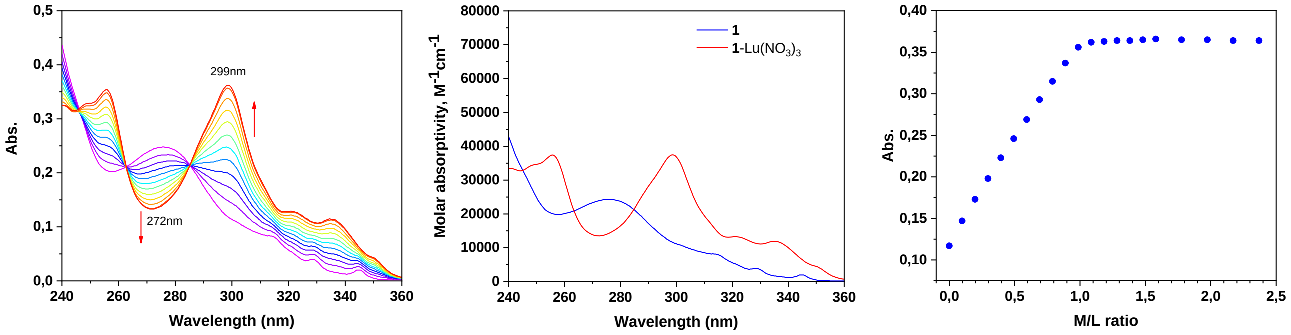

Figure S52. Spectrophotometric titration of **1** (ca. 10^{-5} mol/L) with Lu^{3+} ions (ca. 10^{-3} mol/L) in CH_3CN solution (T = 25.0 ± 0.1 °C, I = 0 M, V_0 = 2.0 mL). Left: absorption spectra. Middle: molar absorptivities of free ligand **1** and Lu(III) complex calculated from spectral deconvolution. Right: titration curve at 299 nm (maximum absorption).

Ligand 2

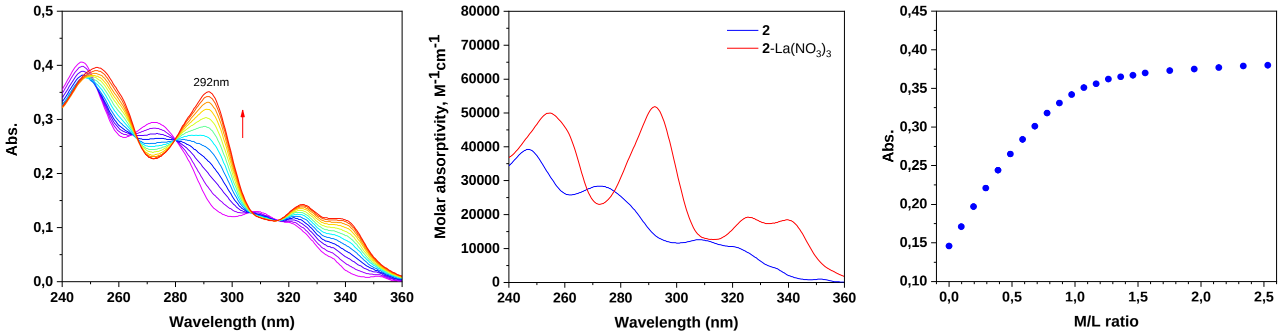

Figure S53. Spectrophotometric titration of **2** (ca. 10^{-5} mol/L) with La^{3+} ions (ca. 10^{-3} mol/L) in CH_3CN solution (T = 25.0 ± 0.1 °C, I = 0 M, V_0 = 2.0 mL). Left: absorption spectra. Middle: molar absorptivities of free ligand **2** and La(III) complex calculated from spectral deconvolution. Right: titration curve at 292 nm (maximum absorption).

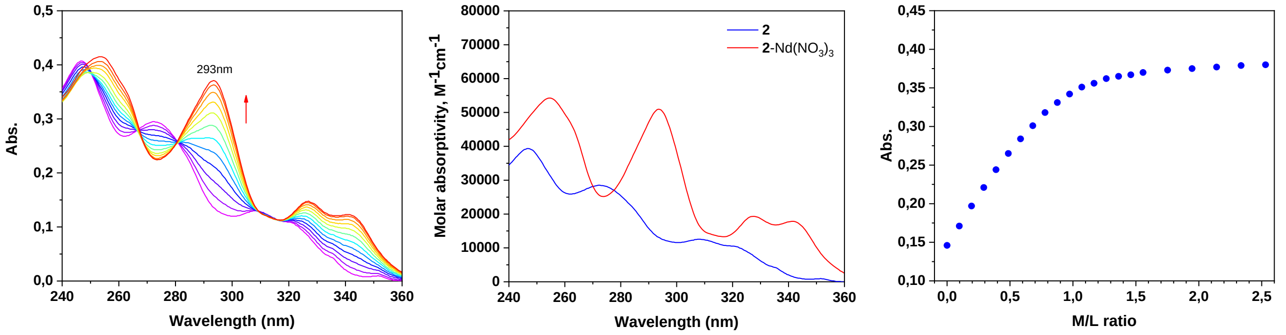

Figure S54. Spectrophotometric titration of **2** (ca. 10^{-5} mol/L) with Nd^{3+} ions (ca. 10^{-3} mol/L) in CH_3CN solution (T = 25.0 ± 0.1 °C, I = 0 M, V_0 = 2.0 mL). Left: absorption spectra. Middle: molar absorptivities of free ligand **2** and Nd(III) complex calculated from spectral deconvolution. Right: titration curve at 293 nm (maximum absorption).

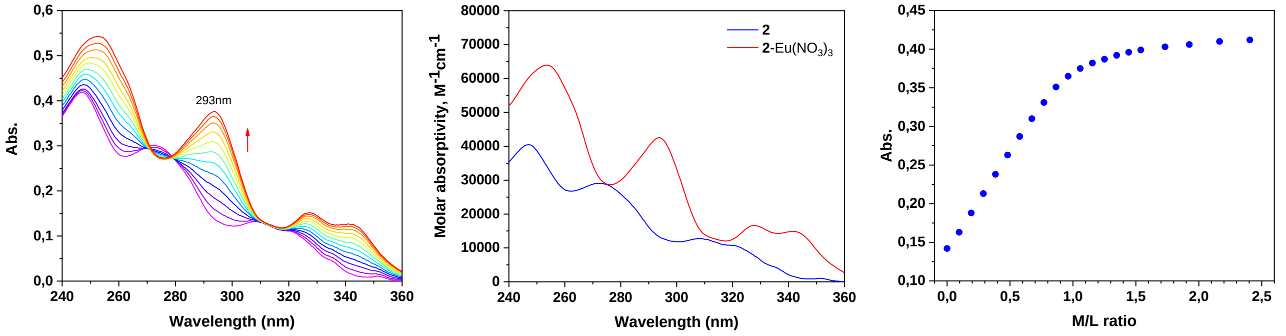

Figure S55. Spectrophotometric titration of **2** (ca. 10^{-5} mol/L) with Eu^{3+} ions (ca. 10^{-3} mol/L) in CH_3CN solution (T = 25.0 ± 0.1 °C, I = 0 M, V_0 = 2.0 mL). Left: absorption spectra. Middle: molar absorptivities of free ligand **2** and Eu(III) complex calculated from spectral deconvolution. Right: titration curve at 293 nm (maximum absorption).

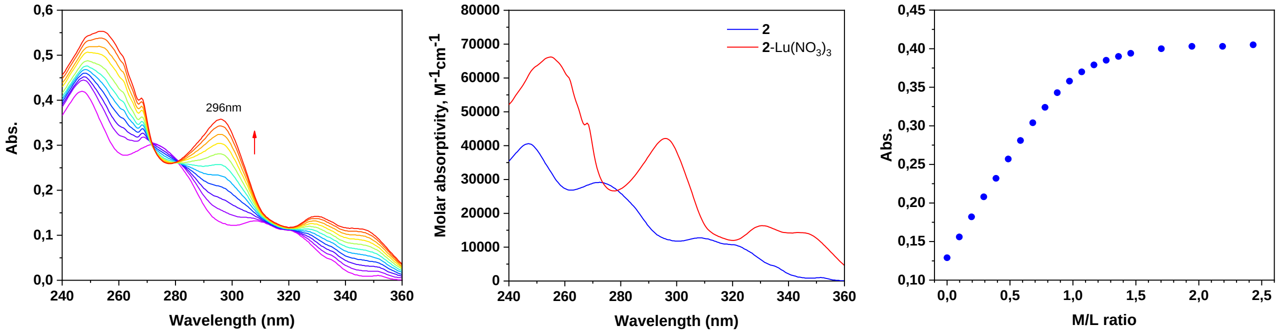

Figure S56. Spectrophotometric titration of **2** (ca. 10^{-5} mol/L) with Lu^{3+} ions (ca. 10^{-3} mol/L) in CH_3CN solution (T = 25.0 ± 0.1 °C, I = 0 M, V_0 = 2.0 mL). Left: absorption spectra. Middle: molar absorptivities of free ligand **2** and Lu(III) complex calculated from spectral deconvolution. Right: titration curve at 296 nm (maximum absorption).

3. **X-ray analysis data**

Analysis of anisotropic displacement parameters and Fourier density synthesis have revealed that in **1** and **2** the pyrrolidine ring is disordered by two positions. The refinement of two positions of carbon atoms were performed with the EADP and DFIX constraints. The positions of water hydrogen atoms were found in an electron density-difference map and refined with isotropic displacement parameters. Crystal data, data collection and structure refinement details are summarized in Table S1.

Table S1. Crystallographic data for **1A, 1B•H_2O and 2B**

	**1A**	**1B•H_2O**	**2B**
Empirical formula	$C_{24}H_{22}F_6N_4O_3$	$C_{24}H_{20}F_6N_4O_2$	$C_{24}H_{18}Cl_2F_6N_4O_2$
Formula weight	528.45	510.44	579.32
Temperature (K)	120	100	111
Crystal system	Monoclinic	Triclinic	Monoclinic
Space group	P21/c	P-1	C2/c
a (Å)	10.3933(9)	7.7456(5)	22.0858(8)
b (Å)	6.9536(8)	9.6214(6)	18.0454(6)
c (Å)	31.848(3)	15.5122(10)	13.008(3)
α (º)	90	106.285(2)	90
β (º)	91.663(5)	91.363(2)	113.529(2)
γ (º)	90	97.650(2)	90
Volume (Å³)	2300.7(4)	1097.49(12)	4753.3(11)
Z(Z’)	4(1)	2(1)	8(1)
d_{calc}, g·cm^{-3}	1.526	1.545	1.619
μ, cm^{-1}	1.35	1.35	3.53
F(000)	1088	524	2352
$2\theta_{max}$, °	52	58	58
Complentess to Θ_{max}	0.995	0.998	0.998
Refl. collected	16595	17516	16358
Refl. unique (R_{int})	4503(0.1130)	6390 (0.0667)	6311 (0.066)
Refl. with I > 2σ(I)	2310	4434	4169
Parameters	338	329	344
R_1 with I>2σ(I)	0.0792	0.0662	0.0644
wR_2 (all data)	0.1896	0.1839	0.1834
Goodness-of-fit on F^2	0.935	1.035	1.046
Largest difference in peak / hole (e/Å³)	0.328/-0.313	0.491/-0.344	0.974/-0.443
CCDC	2159821	2159822	2159823

5. Theoretical computations

Cartesian coordinates

A(R,R)

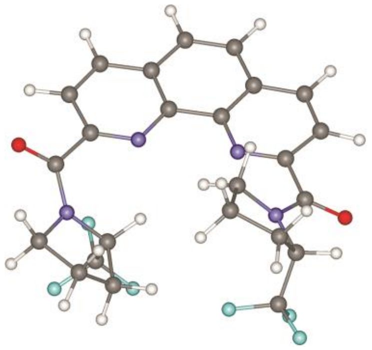

cartesian

set=L1

8	-4.40573509	-1.54875451	0.14641297
8	4.32728359	1.09720827	-0.33366149
9	-3.14976796	2.45954764	0.10837650
9	-1.65849286	3.21752169	1.52084609
9	-3.58953749	2.47647790	2.24671988
9	2.11635563	0.16155416	3.46163914
9	3.34204079	1.73226943	2.56762800
9	4.24010747	0.31709369	3.97535967
7	-1.60970849	-0.06614511	-1.29450192
6	-2.91260661	-0.29622176	-1.19467168
6	-3.79243385	-0.28166944	-2.30223317
1	-4.84320133	-0.52790006	-2.14222998
6	-1.09295335	0.21612165	-2.51055372
6	-1.90098555	0.30465254	-3.68292702
6	-3.28415699	0.04004249	-3.54191398
1	-3.93251021	0.08995505	-4.42185453
6	0.34091243	0.44902273	-2.62245923
6	0.88612297	0.79865479	-3.89512041
6	0.03348633	0.89179781	-5.04014866
1	0.47886353	1.16044716	-6.00280878
6	-1.30730369	0.64800833	-4.93870301
1	-1.95259537	0.71437820	-5.81993696
6	2.27843252	1.04289952	-3.96128814
1	2.72910790	1.32412643	-4.91787603

6	3.04417990	0.92785621	-2.82128277
1	4.11628052	1.13077025	-2.80404985
6	2.40895212	0.53838902	-1.61818802
7	1.10509156	0.31601445	-1.51702624
6	-3.50118843	-0.71647161	0.14094164
7	-2.98915310	-0.18381931	1.29619596
6	-1.97830716	0.86930442	1.46671479
1	-1.21484646	0.78017029	0.67927103
6	-1.41295033	0.64591069	2.89806028
1	-0.31555453	0.62131323	2.89022836
1	-1.72842867	1.46891931	3.55716010
6	-2.03661425	-0.68412019	3.36963710
1	-1.39691427	-1.53455218	3.08325129
6	-3.35816377	-0.75733010	2.59783820
1	-3.74699536	-1.77207339	2.43691978
1	-4.14452797	-0.15101205	3.08236481
6	-2.60326783	2.26207680	1.33317965
6	3.28455818	0.45103453	-0.38374682
7	2.85142392	-0.35130359	0.65313950
6	3.74497248	-0.50024480	1.80530531
1	4.76073765	-0.19323358	1.50378880
6	3.63188294	-1.98415017	2.18760218
1	4.36826572	-2.55388854	1.59897234
1	3.84028566	-2.15534028	3.25385083
6	2.19634077	-2.35595402	1.77828404
1	2.08114706	-3.43023706	1.57210290
1	1.49492096	-2.08109861	2.57919284
6	1.92736394	-1.51067747	0.52185236
1	2.17225421	-2.07407880	-0.39472714
1	0.88708854	-1.17240452	0.43746115
6	3.35410444	0.44139762	2.95209697
1	-2.18366276	-0.71225511	4.45951508

$end

Energy = -1893.6146907992

B(R,R)

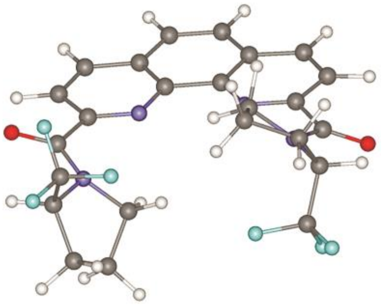

cartesian

set=L1

**8**	**-4.47069134**	**-0.91692256**	**0.14029828**
**8**	**4.48232508**	**1.04267302**	**-0.54435020**
**9**	**-3.00084778**	**-2.76604164**	**2.16593734**
**9**	**-3.22547904**	**-2.04544199**	**4.22040487**
**9**	**-1.33227847**	**-1.76029085**	**3.15158692**
**9**	**2.01226693**	**0.58150271**	**3.16480576**
**9**	**3.39477834**	**1.99925462**	**2.24702920**
**9**	**4.09057393**	**0.68360777**	**3.85202218**
**7**	**-1.47903721**	**0.11873498**	**-1.40876807**
**6**	**-2.79252241**	**-0.01267327**	**-1.27442540**
**6**	**-3.69919478**	**0.06622381**	**-2.35860537**
**1**	**-4.76142803**	**-0.08770511**	**-2.16147080**
**6**	**-0.97832095**	**0.35081627**	**-2.64139373**
**6**	**-1.80409098**	**0.46877420**	**-3.79916186**
**6**	**-3.19980914**	**0.31798086**	**-3.61853572**
**1**	**-3.86535808**	**0.39761079**	**-4.48341081**
**6**	**0.46444937**	**0.49009325**	**-2.78779064**
**6**	**1.00952409**	**0.75370591**	**-4.07985511**
**6**	**0.14095329**	**0.86518815**	**-5.21197974**
**1**	**0.58400997**	**1.06539014**	**-6.19238067**
**6**	**-1.21236657**	**0.72660104**	**-5.07681121**
**1**	**-1.86844134**	**0.81193670**	**-5.94841202**
**6**	**2.41469519**	**0.89986474**	**-4.17365745**
**1**	**2.86869480**	**1.10857462**	**-5.14714347**
**6**	**3.18874666**	**0.78700941**	**-3.03793540**
**1**	**4.27175260**	**0.92238373**	**-3.04342150**

**6**	**2.54624396**	**0.49909116**	**-1.81039628**
**7**	**1.23370696**	**0.36102863**	**-1.68818113**
**6**	**-3.38029415**	**-0.35493319**	**0.08188339**
**7**	**-2.62938174**	**-0.06647260**	**1.20353545**
**6**	**-3.20721566**	**-0.40398910**	**2.51082158**
**1**	**-4.29749721**	**-0.51481794**	**2.38612568**
**6**	**-2.79748049**	**0.74942993**	**3.46320425**
**1**	**-3.65421862**	**1.10258527**	**4.05300040**
**1**	**-2.02270586**	**0.40088297**	**4.16318772**
**6**	**-2.22204090**	**1.83153494**	**2.53018041**
**1**	**-3.02411027**	**2.49144618**	**2.16125625**
**6**	**-1.64256381**	**1.02170845**	**1.36393863**
**1**	**-1.53769021**	**1.58751900**	**0.43337395**
**1**	**-0.65453422**	**0.60466769**	**1.62142492**
**6**	**-2.68889207**	**-1.75755611**	**3.00508562**
**6**	**3.40920969**	**0.44569076**	**-0.56490852**
**7**	**2.91061976**	**-0.24914959**	**0.51650086**
**6**	**3.73310058**	**-0.31173315**	**1.72640817**
**1**	**4.77448268**	**-0.06434633**	**1.46005981**
**6**	**3.54721570**	**-1.74754234**	**2.24086747**
**1**	**4.29542681**	**-2.39156005**	**1.75220564**
**1**	**3.69211061**	**-1.81999238**	**3.32864620**
**6**	**2.12322819**	**-2.11626235**	**1.78953939**
**1**	**1.98982073**	**-3.20104570**	**1.66675985**
**1**	**1.38634820**	**-1.76326703**	**2.52406617**
**6**	**1.93758732**	**-1.37542953**	**0.45386028**
**1**	**2.18892278**	**-2.02604653**	**-0.40112814**
**1**	**0.91763522**	**-1.00073428**	**0.30303875**
**6**	**3.29980864**	**0.74942353**	**2.74747840**
**1**	**-1.45974671**	**2.45101842**	**3.02558945**

$end

Energy = -1893.6146319762

C(R,R)

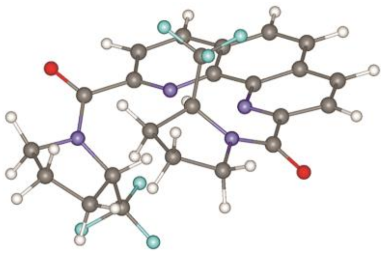

cartesian

set=L1

8	-4.33128570	-1.41009911	0.19730228
8	4.34822881	1.35541102	-0.01705197
9	-3.08458568	2.61483511	0.02279821
9	-1.66632107	3.43767167	1.47389124
9	-3.63737582	2.73785624	2.13094715
9	3.62157875	-3.21299725	0.72193153
9	2.82342160	-2.41991608	-1.14701497
9	1.52769583	-3.57493725	0.18821624
7	-1.48319940	0.03966970	-1.18281853
6	-2.78763317	-0.20029020	-1.12799300
6	-3.62351146	-0.23084797	-2.26838299
1	-4.67657411	-0.48634961	-2.14197888
6	-0.92209326	0.29255276	-2.38688162
6	-1.68856715	0.34706637	-3.58982393
6	-3.07197691	0.06588248	-3.49523031
1	-3.68629781	0.08539670	-4.40037799
6	0.51326858	0.53283604	-2.45634116
6	1.09379477	0.87868376	-3.71372738
6	0.28032588	0.94390558	-4.88845595
1	0.75567044	1.20634480	-5.83836563
6	-1.05816782	0.67746995	-4.83059998
1	-1.67380002	0.71731251	-5.73423750
6	2.48320908	1.14467289	-3.73946665
1	2.95781274	1.42625953	-4.68423329
6	3.21623401	1.04713768	-2.57738749
1	4.28318809	1.27013365	-2.53140962
6	2.55561594	0.63967891	-1.39497231

**7**	**1.25078771**	**0.40464286**	**-1.33047301**
**6**	**-3.43533524**	**-0.56961614**	**0.19489558**
**7**	**-2.99430522**	**0.02931820**	**1.34872309**
**6**	**-1.99464873**	**1.09078583**	**1.51884716**
**1**	**-1.18957274**	**0.96104845**	**0.77934125**
**6**	**-1.51248369**	**0.94412103**	**2.99053192**
**1**	**-0.41504912**	**0.92367830**	**3.04372257**
**1**	**-1.85996230**	**1.80161132**	**3.58644278**
**6**	**-2.16275273**	**-0.35991386**	**3.49695518**
**1**	**-1.50971134**	**-1.22373206**	**3.29304824**
**6**	**-3.43757311**	**-0.47584470**	**2.65493124**
**1**	**-3.81623459**	**-1.49882312**	**2.52555912**
**1**	**-4.24980992**	**0.15254474**	**3.06235477**
**6**	**-2.60601712**	**2.47654372**	**1.28179730**
**6**	**3.40505495**	**0.57688690**	**-0.13808503**
**7**	**3.10054965**	**-0.32914323**	**0.84468734**
**6**	**2.06632902**	**-1.36653676**	**0.86570613**
**1**	**1.17216967**	**-1.01269177**	**0.32845564**
**6**	**1.82889594**	**-1.59016888**	**2.36908122**
**1**	**1.08547322**	**-0.85381760**	**2.71268209**
**1**	**1.43694057**	**-2.59616188**	**2.57947328**
**6**	**3.20253617**	**-1.32193911**	**3.01355243**
**1**	**3.11345558**	**-0.98136322**	**4.05526003**
**1**	**3.80907166**	**-2.23808844**	**3.00263680**
**6**	**3.84348978**	**-0.24611343**	**2.11923988**
**1**	**3.73174853**	**0.77160725**	**2.52975249**
**1**	**4.91896231**	**-0.41503179**	**1.95285104**
**6**	**2.52131679**	**-2.64931465**	**0.15307700**
**1**	**-2.37198085**	**-0.32982784**	**4.57661697**

$end

Energy = -1893.6136306699

A(R,S)

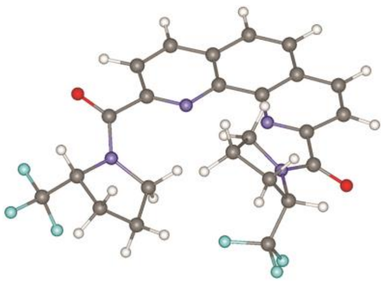

cartesian

set=L1

8	-4.19777189	-1.44746691	0.47267001
8	4.40083085	1.20695212	-0.89567322
9	2.47990209	0.29086355	3.06816542
9	3.59551858	1.88132863	2.07090330
9	4.62533133	0.52440799	3.44546922
7	-1.57417071	0.00811333	-1.26464911
6	-2.86177779	-0.20150218	-1.02680994
6	-3.85193267	-0.18886718	-2.03939924
1	-4.88442199	-0.41768595	-1.77057173
6	-1.18249980	0.26492824	-2.53200598
6	-2.10448924	0.34681023	-3.61724267
6	-3.46960814	0.10596099	-3.33007148
1	-4.20454079	0.15078275	-4.13943260
6	0.23476137	0.48015072	-2.79233291
6	0.65248715	0.80214491	-4.11930157
6	-0.31055926	0.88310081	-5.17429674
1	0.03782841	1.12811871	-6.18221298
6	-1.63709454	0.65914534	-4.93316033
1	-2.36725830	0.71782664	-5.74609532
6	2.03280433	1.03520019	-4.32808330
1	2.38788218	1.29329408	-5.33037026
6	2.90676051	0.94258429	-3.26627541
1	3.97533967	1.14360999	-3.35988221
6	2.39168114	0.58184465	-1.99861737
7	1.10420784	0.36378341	-1.76584079
6	-3.32080528	-0.59287713	0.36737929

**7**	**-2.75058838**	**0.02637006**	**1.45192764**
**6**	**-3.09239919**	**-0.45113591**	**2.79212781**
**6**	**-4.26737632**	**0.32413980**	**3.40918780**
**6**	**-1.78616007**	**-0.26722421**	**3.58046511**
**1**	**-1.96435461**	**-0.16804468**	**4.66120493**
**1**	**-1.15682347**	**-1.15607348**	**3.41672013**
**6**	**-1.14521472**	**0.98646149**	**2.95419837**
**1**	**-1.51327067**	**1.88948265**	**3.45993319**
**6**	**-1.60217931**	**0.96949801**	**1.47921111**
**1**	**-1.91945123**	**1.97063715**	**1.14383618**
**1**	**-0.82426146**	**0.61335434**	**0.79263183**
**1**	**-3.43079210**	**-1.49752027**	**2.72052598**
**6**	**3.37518964**	**0.53404276**	**-0.84606640**
**7**	**3.04831271**	**-0.25787266**	**0.23578629**
**6**	**4.02306768**	**-0.35197583**	**1.32509889**
**1**	**5.00428938**	**-0.01589922**	**0.94941551**
**6**	**3.98968710**	**-1.83091362**	**1.74035732**
**1**	**4.70244050**	**-2.38642484**	**1.11051141**
**1**	**4.27882797**	**-1.97407071**	**2.79179674**
**6**	**2.54253229**	**-2.25946096**	**1.44116493**
**1**	**2.45207796**	**-3.34045656**	**1.25939156**
**1**	**1.89082258**	**-1.99582883**	**2.28638983**
**6**	**2.15244982**	**-1.44531049**	**0.19559373**
**1**	**2.34286295**	**-2.01758075**	**-0.72842837**
**1**	**1.09942675**	**-1.13745219**	**0.18820430**
**6**	**3.67367735**	**0.59895458**	**2.47795271**
**1**	**-0.04881532**	**0.96990487**	**3.02825399**
**9**	**-4.55028804**	**-0.17640136**	**4.64263751**
**9**	**-3.99466586**	**1.64954431**	**3.56475046**
**9**	**-5.38742895**	**0.23470440**	**2.66295744**

$end

Energy = -1893.6128633253

B(R,S)

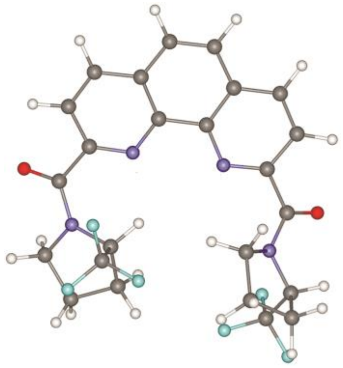

cartesian

set=L1

8	-5.00611878	0.69285337	0.29719973
8	3.82000912	2.05009179	-0.59523418
9	2.15736532	0.50590262	3.33925377
9	2.76312017	2.38694482	2.41064567
9	4.12940299	1.35397398	3.77452152
7	-1.86499414	0.24451595	-1.24213953
6	-3.17471581	0.09996136	-1.08315367
6	-4.06466482	-0.13824919	-2.15768752
1	-5.13028789	-0.22044222	-1.93994671
6	-1.34975520	0.19362927	-2.49048341
6	-2.16509337	-0.03091386	-3.64113834
6	-3.55159397	-0.21979994	-3.43256753
1	-4.20242365	-0.40464570	-4.29253948
6	0.08261073	0.40520122	-2.67214094
6	0.60115146	0.45670309	-4.00294009
6	-0.25494590	0.21869486	-5.12387377
1	0.18020651	0.24579883	-6.12746968
6	-1.58594775	-0.03036837	-4.94861180
1	-2.23787385	-0.21121091	-5.80858399
6	1.97355266	0.76074823	-4.16014055
1	2.39695733	0.82603349	-5.16689634
6	2.74884907	0.98692277	-3.04447312
1	3.80008935	1.27365327	-3.10901232
6	2.15455773	0.83250909	-1.77027203
7	0.87016753	0.54981575	-1.58041977

6	-3.83058947	0.33096803	0.27069282
7	-3.11083000	0.19453181	1.42716212
6	-1.74039729	-0.28060175	1.62743672
6	-1.69931422	-1.81014483	1.73894266
6	-1.31442801	0.42340815	2.92602610
1	-0.51663587	-0.11991246	3.45161950
1	-0.92572222	1.41837367	2.66038644
6	-2.61730227	0.53608677	3.74192565
1	-2.76545815	-0.36818089	4.34838139
6	-3.73147936	0.65988747	2.68564794
1	-4.61778945	0.05312659	2.92986775
1	-4.07446794	1.69802375	2.54358712
1	-1.12257484	-0.00849297	0.75648513
6	3.03955528	1.10414106	-0.56950135
7	2.91272152	0.25029120	0.50271700
6	3.84709898	0.40783348	1.61932769
1	4.69289555	1.03069100	1.28118794
6	4.24266241	-1.03107391	1.98840257
1	5.10636309	-1.32482996	1.37112034
1	4.53114631	-1.12433817	3.04563815
6	2.99704143	-1.85789512	1.62281700
1	3.23858160	-2.90751682	1.40016546
1	2.27274713	-1.83962622	2.44852536
6	2.40994724	-1.14337613	0.39322208
1	2.77649692	-1.59435629	-0.54504411
1	1.31341246	-1.16414369	0.38014556
6	3.21199092	1.17223063	2.78746341
1	-2.60566025	1.40333624	4.41764795
9	-0.41527007	-2.23554248	1.92426118
9	-2.43162540	-2.29428894	2.77677792
9	-2.16274085	-2.40693281	0.61506860

$end

Energy = -1893.6144936751

C(R,S)

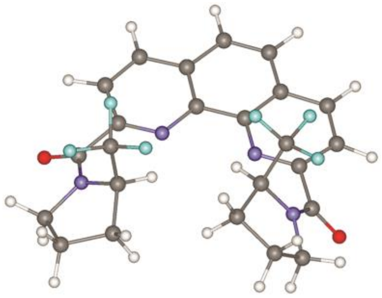

cartesian

set=L1

8	-4.86620368	1.17788562	0.25743015
8	3.99536496	2.12865119	-0.57798740
9	4.25313360	-2.35680752	0.75374790
9	3.14591254	-1.98814142	-1.08977192
9	2.23083768	-3.16373120	0.51342709
7	-1.73637379	0.44382018	-1.17059695
6	-3.05718272	0.37525915	-1.04329175
6	-3.93589593	0.14765052	-2.12847837
1	-5.00918259	0.13295348	-1.93551363
6	-1.19634523	0.32437281	-2.40685982
6	-2.00190186	0.09456219	-3.56583722
6	-3.39999116	-0.01430183	-3.38468353
1	-4.03922900	-0.20042592	-4.25287906
6	0.24751295	0.46688240	-2.57559512
6	0.78111125	0.44494246	-3.90150425
6	-0.06571658	0.19606445	-5.02584343
1	0.38697172	0.16289551	-6.02139062
6	-1.40762453	0.01256846	-4.86274445
1	-2.05639313	-0.17538363	-5.72342504
6	2.16491313	0.68956309	-4.06031253
1	2.59644763	0.69369160	-5.06563201
6	2.93940070	0.93480250	-2.95100769
1	4.00092401	1.17766737	-3.01861928
6	2.33317127	0.85119770	-1.67602093
7	1.03757850	0.62371041	-1.48337629
6	-3.74004333	0.68443929	0.28032420

**7**	**-3.10151984**	**0.45184367**	**1.46870975**
**6**	**-1.79702111**	**-0.16078158**	**1.71565461**
**6**	**-1.89024821**	**-1.69352390**	**1.75641404**
**6**	**-1.38323835**	**0.44053886**	**3.07081281**
**1**	**-0.68841359**	**-0.21257292**	**3.61897385**
**1**	**-0.88024464**	**1.40161892**	**2.88031860**
**6**	**-2.71562553**	**0.65502883**	**3.81529581**
**1**	**-2.99833865**	**-0.26017220**	**4.35368443**
**6**	**-3.73498306**	**0.95818433**	**2.70368164**
**1**	**-4.70665327**	**0.46760585**	**2.87125183**
**1**	**-3.92892873**	**2.03720888**	**2.58420425**
**1**	**-1.11156322**	**0.09098814**	**0.88979011**
**6**	**3.23821251**	**1.16464343**	**-0.49808227**
**7**	**3.20355564**	**0.37052603**	**0.61481635**
**6**	**2.39090187**	**-0.82065360**	**0.86194664**
**1**	**1.40120771**	**-0.70656414**	**0.39316661**
**6**	**2.32812021**	**-0.88199532**	**2.39901206**
**1**	**1.49394632**	**-0.24571105**	**2.73065266**
**1**	**2.14679534**	**-1.90270348**	**2.76425702**
**6**	**3.67437317**	**-0.29008029**	**2.85466712**
**1**	**3.61629874**	**0.14611235**	**3.86237337**
**1**	**4.44910082**	**-1.06935477**	**2.86117683**
**6**	**3.99605232**	**0.77544908**	**1.79415601**
**1**	**3.68636073**	**1.78852850**	**2.10235358**
**1**	**5.06689897**	**0.82185405**	**1.54179444**
**6**	**3.01632227**	**-2.08815591**	**0.25563699**
**1**	**-2.65496849**	**1.47712145**	**4.54288672**
**9**	**-0.65409829**	**-2.22972083**	**1.95646017**
**9**	**-2.69319783**	**-2.15253754**	**2.75363957**
**9**	**-2.37030022**	**-2.19751368**	**0.59673638**

$end

Energy = -1893.6126649520

ESP maps of ligands

Ligand 1

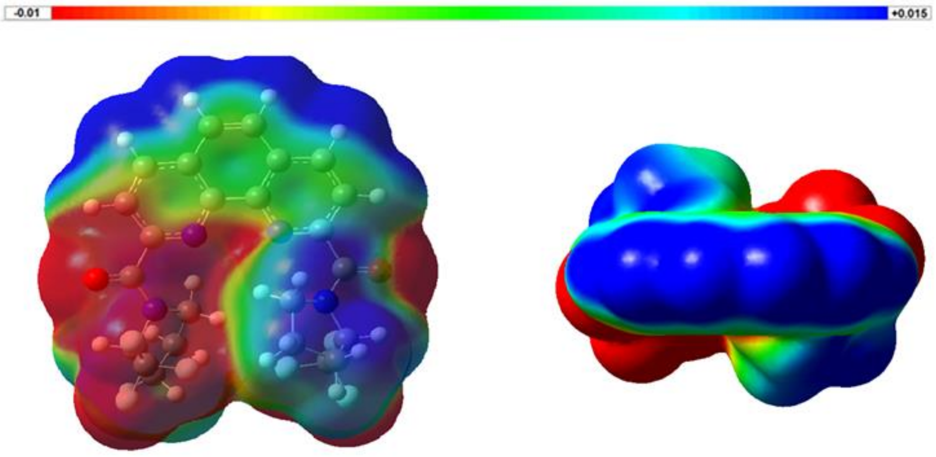

E=-1895.206201 hartree

H	2.95713305	-5.13783789	0.16621357
H	5.89797020	0.98966515	-0.20427443
N	0.85759896	-1.87049079	0.09374858
N	-1.94586802	-2.00948691	0.55539358
N	2.03957200	0.58181614	-0.10978642
N	0.40057600	2.83890700	-0.58297044
O	-1.78795910	-4.15765381	-0.19562542
O	2.10414100	4.03512812	0.36185458
C	0.27354199	-3.05540180	0.18374357
C	0.99117297	-4.27437401	0.20036757
H	0.44146198	-5.20657206	0.24475157
C	2.36684585	-4.22549772	0.14653757
C	3.02216387	-2.97500682	0.05952357
C	4.44931841	-2.84153700	0.00144457
H	5.05519199	-3.74318480	0.02475057
C	5.03949833	-1.61477578	-0.07797942
H	6.12177801	-1.52628183	-0.11622442
C	4.25223207	-0.41616583	-0.11049842
C	4.81711531	0.87947416	-0.17735544
C	3.99488378	1.98541427	-0.19591443
H	4.38028812	2.99768806	-0.21516043
C	2.59577584	1.78032124	-0.17627743
C	2.83378792	-0.50533783	-0.06741443

C	2.20396900	-1.81249678	0.02918757
C	-1.23704505	-3.12441587	0.18170857
C	1.68508697	2.98524117	-0.12331642
C	-1.52101398	-0.92160183	1.46103656
H	-1.41410208	0.01764217	0.90964758
H	-0.56059504	-1.14878583	1.91613960
C	-2.67918897	-0.84914583	2.46322751
H	-2.55809617	-1.62615275	3.22610545
H	-2.72806716	0.11881917	2.96875167
C	-3.92263317	-1.12897277	1.60104454
H	-4.30260897	-0.19657482	1.17591262
H	-4.73677683	-1.59453082	2.15975952
C	-3.40895796	-2.05719280	0.47018558
H	-3.74007607	-3.09402800	0.57153755
C	-3.88582301	-1.57847583	-0.89862943
C	-0.52234602	3.96471715	-0.45679444
H	0.04327300	4.89413500	-0.35711142
C	-1.35222602	3.83402920	0.82167459
C	-1.37828588	3.89495015	-1.73079944
C	-1.43083000	2.39230609	-2.05933738
C	-0.08112700	1.83403921	-1.56841838
H	-1.57856488	2.20368910	-3.12565136
H	-2.25059700	1.91170418	-1.52297544
H	-0.86297899	4.44853830	-2.52198148
H	-2.36361408	4.34652185	-1.59631348
H	-0.17079601	0.85027915	-1.11017239
H	0.64330000	1.75513816	-2.38672853
F	-5.23660803	-1.62098479	-0.96050745
F	-3.51488709	-0.29277283	-1.13441348
F	-3.40367508	-2.32097197	-1.90708041
F	-2.21617317	4.86927700	0.93120760
F	-2.09411502	2.69459915	0.84204555
F	-0.58204496	3.82266116	1.92221355

Ligand 2

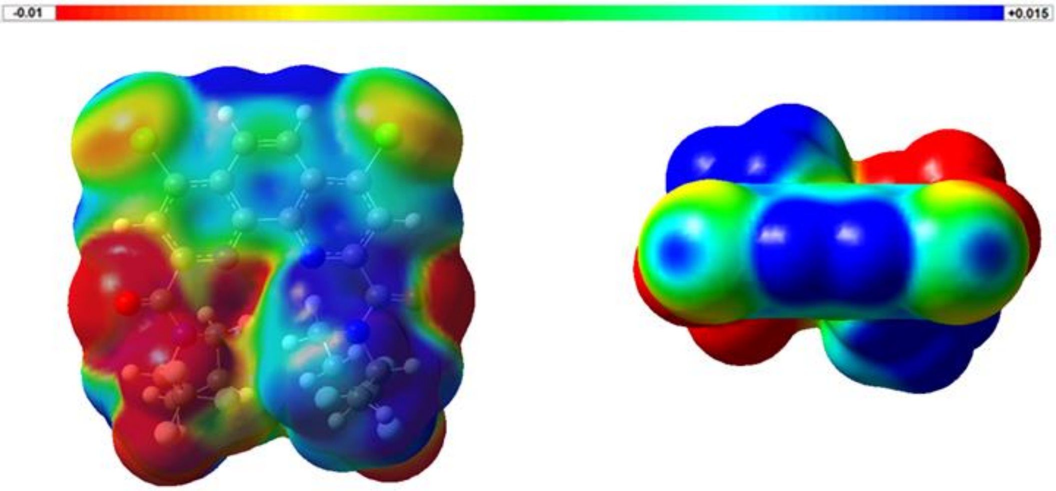

E=-2814.389905 hartree

Cl	-5.07246351	4.19425011	0.17345269
Cl	-5.68374538	-3.39481258	-0.20536931
N	-1.46763229	1.43522632	0.09714069
N	1.11376870	2.55002928	0.53962666
N	-1.68735528	-1.24852574	-0.09313630
N	0.66628873	-2.76779270	-0.54855335
O	0.19890764	4.51568317	-0.17222032
O	-0.49210933	-4.47892284	0.43161771
C	-1.33337128	2.74747419	0.18716569
C	-2.42626834	3.64232540	0.20038669
H	-2.24329948	4.70812798	0.24198268
C	-3.69482541	3.11172438	0.14277369
C	-3.89134836	1.70800722	0.05692569
C	-5.17065573	1.06824625	-0.00278931
H	-6.06133175	1.68495429	0.01485769
C	-5.28121042	-0.28926873	-0.07640930
H	-6.26007271	-0.75239873	-0.11499631
C	-4.12132120	-1.12790370	-0.10063031
C	-4.15089560	-2.54678154	-0.15694931
C	-2.98326135	-3.27633286	-0.16357131
H	-2.97685838	-4.35857582	-0.16893432
C	-1.76265430	-2.56658459	-0.14744732
C	-2.82761931	-0.53412473	-0.05864231

C	-2.70913839	0.91476828	0.03183569
C	0.05624068	3.34852648	0.18754868
C	-0.47637033	-3.36021471	-0.07799631
C	1.10779667	1.36449420	1.42315173
H	1.31174564	0.45620325	0.84873867
H	0.14001766	1.24281931	1.90256870
C	2.24699259	1.66947126	2.40247464
H	1.88889265	2.33930922	3.19187164
H	2.63599777	0.76346028	2.87410164
C	3.29674268	2.37704849	1.52835572
H	3.95510077	1.63938522	1.06296468
H	3.92263961	3.07486248	2.08764768
C	2.46764779	3.10681438	0.44056872
H	2.42246962	4.19020414	0.57959169
C	3.04950380	2.86891747	-0.95106131
C	1.93521976	-3.47969484	-0.40579230
H	1.74558473	-4.54943609	-0.29007730
C	2.65539670	-3.03653765	0.86917168
C	2.71197581	-3.12510681	-1.68335032
C	2.21102071	-1.71535575	-2.04385734
C	0.74841368	-1.68117976	-1.56160438
H	2.28442860	-1.50816572	-3.11433029
H	2.79469562	-0.95614970	-1.52103329
H	2.44120765	-3.84532976	-2.46164942
H	3.79344153	-3.17995882	-1.54144132
H	0.46799865	-0.72238374	-1.12817228
H	0.05002666	-1.89601970	-2.37816834
F	4.30304623	3.37096119	-1.02013934
F	3.13205171	1.54294527	-1.23642731
F	2.32262659	3.43800116	-1.92469633
F	3.83291554	-3.68817663	0.99609768
F	2.93647981	-1.70549476	0.86690366
F	1.92652464	-3.28301096	1.96991968
